# Waist-to-height ratio modifies the association between cardiometabolic indices and incident carotid plaque: evidence from a Chinese cohort

**DOI:** 10.3389/fnut.2026.1853867

**Published:** 2026-07-07

**Authors:** Shusheng Fang, Yan Li, Hong Chen, Jingshan Jiang, Song Leng

**Affiliations:** Health Management Center, The Second Hospital of Dalian Medical University, Dalian Medical University, Dalian, Liaoning, China

**Keywords:** cardiometabolic indices, carotid plaque, cohort study, risk stratification, waist-to-height ratio

## Abstract

**Background:**

Cardiometabolic indices and central obesity, often measured by waist-to-height ratio (WHtR), are both associated with carotid plaque (CP), but are usually analyzed separately. However, it remains unclear whether their combined effects—both additive and multiplicative—differ in relation to incident CP within the same population, and whether potential interactions exist.

**Methods:**

This retrospective cohort study was based on the Dalian Health Management Cohort. Cox proportional hazards models were used to assess the associations of cardiometabolic indices and WHtR with incident CP, and both additive and multiplicative models were applied to evaluate their joint effects and interactions. Time-dependent and time-weighted average exposure models based on repeated measurements were used to reduce regression dilution bias. Dose–response relationships were examined using restricted cubic splines. Predictive performance and clinical utility were further evaluated using time-dependent receiver operating characteristic curves, Harrell's C-index, decision curve analysis, and related metrics.

**Results:**

Among 6,984 participants, 1,198 incident CP cases occurred over a median follow-up of 2.4 years (incidence rate: 62.2 per 1,000 person-years). Multiple cardiometabolic indices were positively associated with the risk of incident CP. Multiplicative indices showed larger effect estimates than additive indices, with the highest risk observed for Castelli's Risk Index II (CRI-II)–WHtR in the top quartile (HR = 2.00, 95% CI 1.63–2.46), and similar effect sizes were observed in both sexes. Results were robust in time-dependent and time-weighted analyses, with a significant interaction between CRI-II and WHtR, and a non-linear association with CP risk. WHtR-based multiplicative composite indices demonstrated moderate discriminatory ability, with CRI-II–WHtR showing the best predictive performance (AUC = 0.770; Harrell's C-index = 0.739), along with modest clinical utility and risk stratification capacity.

**Conclusion:**

Cardiometabolic abnormalities and central obesity were jointly associated with a higher risk of incident CP, with a more pronounced association observed for the CRI-II–WHtR composite index. Further interaction analyses suggest a potential interaction between cardiometabolic abnormalities and central obesity in CP development. The predictive value of composite indices still requires further validation before clinical application.

## 1 Introduction

Cardiovascular disease (CVD) remains the leading cause of morbidity and mortality worldwide, affecting over 626 million individuals and causing 19.2 million deaths in 2023 (1, 2). Carotid plaque (CP) is characterized by the accumulation of lipids and fibrous elements in large arteries and represents a key manifestation of atherosclerosis, conferring up to a 4-fold increase in CVD risk (3–5). Despite improvements in imaging technology, the global burden of CP continues to rise due to aging populations and prevalent vascular risk factors, highlighting the critical need for early identification and risk stratification (6).

Traditional lipid measures, including total cholesterol (TC) and low-density lipoprotein cholesterol (LDL-C), are closely associated with CP (7). Beyond these, a range of emerging cardiometabolic indices—such as remnant cholesterol (RC) (8), RC-to-high-density lipoprotein cholesterol (HDL-C) ratio (RC/HDL-C) (9), non-HDL-C (9), the cholesterol–HDL-C–glucose (CHG) index (10), triglyceride (TG)–glucose (TyG) index (11), atherogenic index of plasma (AIP) (11), atherogenic coefficient (AC) (9), and Castelli's indices I and II (CRI-I, CRI-II) (9)—have been increasingly studied in relation to cardiometabolic disorders. In parallel, central obesity, commonly assessed by waist-to-height ratio (WHtR), is also associated with CP (12–14). However, previous studies have largely analyzed metabolic abnormalities and obesity separately, and whether their combined effects better reflect risk of incident CP remains unclear. Moreover, no study has systematically compared which combination of cardiometabolic indices and WHtR is most strongly associated with incident CP within the same population.

In this study, we used a large Chinese cohort to systematically evaluate both additive and multiplicative combinations of cardiometabolic indices and WHtR in relation to incident CP. We further examined potential interactions between metabolic abnormalities and central obesity, and aimed to identify which combined index showed the strongest association with incident CP. By addressing these gaps, our study provides evidence for refining early risk stratification and guiding more precise identification of individuals at higher risk of CP.

## 2 Methods

### 2.1 Study design and participants

This study utilized data from the Dalian Health Management Cohort (DHMC; Cohort number: CCC2023112102; https://cohortconsortium.com/queue/management/2669932f46174321b26fa069449e769c?source=queues), which is a large ongoing cohort study initiated in 2014 (11). The cohort consists of individuals undergoing routine health examinations at the Health Examination Center of a tertiary hospital in Dalian, China (11). The DHMC aims to assess population health and elucidate risk factors influencing health outcomes. This will ultimately provide evidence for identifying groups with a high risk of or susceptibility to developing chronic diseases and for developing targeted prevention and intervention strategies (11).

**Figure 1:**
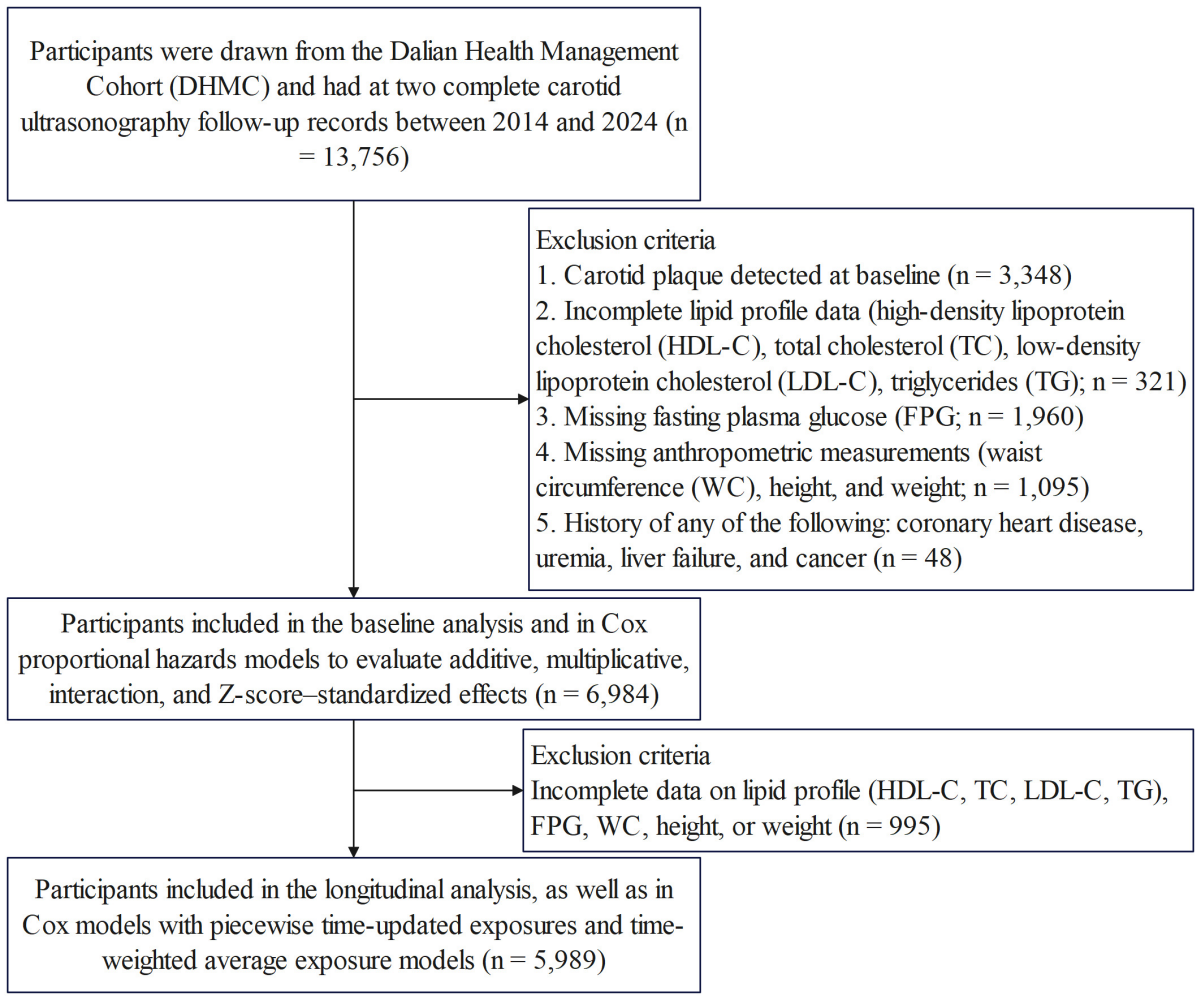
Flowchart of study participants.

From 2014 to 2024, 13,756 adults who completed at least two routine health examinations, including carotid ultrasonography, were screened for inclusion. Exclusion criteria were: (1) CP at baseline (*n* = 3,348); (2) missing lipid profile (HDL-C, TC, LDL-C, TG; *n* = 321), fasting plasma glucose (FPG; *n* = 1,960), or anthropometric measurements [waist circumference (WC), height, and weight; *n* = 1,095]; and (3) history of coronary heart disease, uremia, liver failure, or cancer (*n* = 48). After exclusions, 6,984 participants were included in the baseline analysis and entered Cox regression models to evaluate additive and multiplicative interactions and standardized effects. An additional 995 participants with missing data for calculating cardiometabolic indices (including HDL-C, TC, LDL-C, TG, FPG, WC, height, and weight) at the second examination were excluded, leaving 5,989 participants for the further longitudinal analysis, analyzed using Cox models with time-dependent and time-weighted average exposures (Figure 1). Baseline was defined as the date of the first examination, and participants were followed until CP diagnosis or the last examination, whichever occurred first.

### 2.2 Ethics statement

This study involved human participants and was conducted in accordance with the principles of the Declaration of Helsinki (15). The study was approved by the Ethics Committee of the Second Affiliated Hospital of Dalian Medical University (approval no.: KY2025-603-01). The cohort was registered in the Chinese Clinical Trial Registration System (Registration No. CHiCTR2300073363). Considering the retrospective design and the use of anonymized data, the requirement for informed consent was waived by the ethics committee (16). All patient data were handled in accordance with strict confidentiality and data protection protocols.

### 2.3 Data collection

Height and weight were measured using standardized equipment with participants barefoot and wearing light clothing. Systolic and diastolic blood pressure (SBP and DBP) were measured by trained nurses using standardized procedures. WC was measured by trained nurses at the midpoint between the lower margin of the rib cage and the iliac crest. Fasting blood samples were collected according to a standardized protocol to measure the following parameters: white blood cell count (WBC) and differential cell (lymphocytes, neutrophils, monocytes); red blood cell count (RBC), platelet counts, HDL-C, TC, LDL-C, TG, fasting plasma glucose (FPG), hemoglobin, aspartate aminotransferase (AST), alanine aminotransferase (ALT), serum uric acid, and serum creatinine concentrations. Body mass index (BMI) was calculated as weight (kg) divided by height squared (m^2^), and WHtR was calculated as WC divided by height. The formulas for the cardiometabolic indices are provided in the Supplementary methods (17). The combined cardiometabolic index–WHtR indices were derived using the following multiplicative model: cardiometabolic index–WHtR = cardiometabolic index × WHtR (Supplementary methods). Previous studies have used similar multiplicative combinations of metabolic indices and central obesity (18–20).

### 2.4 Definition of CP

CP was the study outcome and was assessed using carotid color Doppler ultrasonography. Trained sonographers examined the common carotid artery, carotid bifurcation, and internal carotid artery. CP was defined as an intima–media thickness (IMT) >1.5 mm, a focal protrusion into the arterial lumen ≥0.5 mm, or a focal thickening exceeding 50% of the surrounding IMT (21). Incident CP cases were identified using consistent ultrasound criteria during follow-up. All examinations were performed by experienced physicians and independently reviewed and confirmed by at least two physicians. Image interpretation was conducted blinded to participants' clinical and laboratory data, and standardized protocols were applied to minimize inter- and intra-observer variability.

### 2.5 Covariates

Smoking status was assessed by a single question on ever or current smoking, with responses categorized as ever or never smokers. Alcohol consumption was assessed similarly and categorized as ever or never drinkers. Medication use at baseline was defined as the use of antihypertensive, lipid-lowering, or antidiabetic drugs. Blood pressure was measured with an electronic sphygmomanometer after ≥5 min of rest. Hypertension was defined as SBP ≥ 140 mmHg, DBP ≥ 90 mmHg, current antihypertensive use, or self-reported hypertension (22). Diabetes was defined as antidiabetic medication or insulin use, a history of diabetes, FPG ≥ 7.0 mmol/L, or HbA1c ≥6.5% (23). Dyslipidemia was defined as TG > 1.7 mmol/L, TC > 5.2 mmol/L, LDL-C > 3.4 mmol/L, or HDL-C ≤ 1.0 mmol/L (24).

### 2.6 Statistical analysis

Continuous variables were summarized as medians with interquartile ranges, and categorical variables as counts and percentages. Between-group differences were evaluated using standardized mean differences (SMDs), with values > 0.1 indicating meaningful imbalance. Because no established clinical cutoffs are available for the cardiometabolic indices in relation to CP, all indices were categorized into quartiles, with the lowest quartile as the reference.

The distribution of missing data is shown in Supplementary Table S1. Key exposure variables (HDL-C, TC, LDL-C, TG, FPG, WC, height, and weight) were complete after excluding participants with missing baseline data; however, missingness remained in other covariates. Several laboratory variables exhibited limited missingness, with the highest proportions for AST (7.90%) and albumin (4.07%), whereas other variables had minimal missingness (< 1%). Outcome events and follow-up time were complete and were not imputed. Missing continuous variables were handled using multiple imputation by chained equations (MICE) with five imputed datasets and 50 iterations (25). All relevant variables were included as predictors, and the outcome variable was included to improve estimation but was not imputed. The imputed datasets were used for subsequent analyses.

Potential confounders were selected based on a directed acyclic graph (DAG) (Supplementary Figure S1), with consideration of established risk factors for both the exposure and the outcome. Variables such as age, sex, smoking status, alcohol consumption, diabetes, dyslipidemia, hypertension, platelet count, and monocyte count were included as covariates in the multivariable models (26).

Associations between cardiometabolic indices and incident CP were examined using Cox proportional hazards models. Three models were constructed: Model 1 adjusted for age and sex; Model 2 additionally adjusted for smoking status and alcohol consumption; and Model 3 further adjusted for diabetes, dyslipidemia, hypertension, platelet count, and monocyte count. Kaplan–Meier (K–M) curves and log-rank tests were used to compare cumulative incidence across quartiles. Age was modeled using restricted cubic splines (RCS) to account for non-linearity and non-proportional hazards (Supplementary Table S2), which improved model fit and proportional hazards assumptions (Supplementary Table S3). Therefore, in subsequent Cox models, age was modeled as a continuous variable using RCS. Multicollinearity was assessed using variance inflation factors (VIFs), and each cardiometabolic index was analyzed separately to avoid structural collinearity. No evidence of multicollinearity was observed (Supplementary Table S4).

To evaluate the joint effects of metabolic abnormalities and central obesity, composite indices were constructed using three approaches. First, additive indices were defined as cardiometabolic index + WHtR. Second, product-based indices were defined as cardiometabolic index × WHtR. Third, formal interaction analyses were conducted by including mean-centered variables and their interaction terms in Cox models. Sex-stratified analyses were performed for product-based indices. In addition, each exposure variable was standardized to inclusion in Cox models to further assess the robustness of the estimates.

Longitudinal exposure analyses were conducted using repeated measurements (baseline and second examination) to reduce regression dilution bias. The interval between visits was defined individually as the time from baseline to the second examination. In time-dependent Cox proportional hazards models, exposures were updated using a counting-process (start–stop) structure. Baseline values were applied from baseline to the second examination and updated thereafter. Participants who developed CP before the second examination contributed only baseline exposure values, whereas those without events were censored at their last follow-up visit. Robust standard errors were estimated by clustering on individual ID to account for within-subject correlation. The product of cardiometabolic indices and WHtR (cardiometabolic index × WHtR) was used as the primary time-varying exposure, consistent with the main analysis. In addition, an early time-weighted average (TWAvg) exposure was constructed within the first 2 years after baseline to capture early cumulative exposure. If the second examination occurred within this window, TWAvg was calculated as a duration-weighted mean of baseline and updated values; otherwise, baseline values were used. A 2-year landmark approach was applied by excluding participants who developed CP or were censored within the first 2 years, and follow-up was reset at year 2. TWAvg values were calculated separately for cardiometabolic indices and WHtR, and their product was used as the primary exposure, consistent with the main analysis. The proportional hazards assumption was assessed using Schoenfeld residuals, and no violations were observed in either the time-dependent or TWAvg models (all *P* > 0.05; Supplementary Tables S5, S6).

Dose–response relationships were assessed using RCS with knots at the 5th, 35th, 65th, and 95th percentiles. Subgroup analyses were conducted according to age, smoking status, alcohol consumption, BMI, hypertension, diabetes, and dyslipidemia.

Several sensitivity analyses were conducted to test robustness. First, additional adjustment models were fitted by further including medication use, liver and renal function markers, and other potential confounders. Second, participants who developed CP within 2 years of baseline were excluded to reduce potential reverse causation. Third, complete-case analyses were performed to assess the influence of multiple imputation.

To account for varying follow-up time and censoring, time-to-event prediction performance was evaluated using survival-specific metrics. Discrimination was assessed using time-dependent receiver operating characteristic (ROC) curves with corresponding area under the curve (AUC) at 1–5 years, as well as Harrell's concordance index (Harrell's C-index) and Uno's C-index. Model calibration was evaluated at the 3-year prediction horizon using calibration plots comparing observed and predicted risks across deciles, with smoothed curves generated by locally weighted regression. Clinical utility was assessed using decision curve analysis (DCA), with net benefit calculated across threshold probabilities of 0.01–0.50. Prediction error was evaluated using the Brier score over time, and overall performance was summarized by the integrated Brier score (IBS). Incremental predictive value was assessed using continuous net reclassification improvement (NRI) and integrated discrimination improvement (IDI) at 3 years. The baseline model included age, sex, smoking status, alcohol consumption, dyslipidemia, hypertension, diabetes, platelet count, and monocyte count, and the extended model additionally incorporated CRI-II–WHtR. Internal validation was performed using bootstrap resampling (300 iterations) to obtain optimism-corrected performance estimates. Participants were further stratified into high- and low-risk groups based on the median predicted 3-year risk, and K–M curves were used to assess risk discrimination.

Finally, exploratory mediation analysis was conducted to assess whether WHtR may partly explain the association between cardiometabolic indices and CP. All analyses were considered hypothesis-generating. Two-sided *P* < 0.05 was considered statistically significant. Analyses were performed using R version 4.5.1.

## 3 Results

### 3.1 Baseline characteristics of the study cohort

Among the 6,984 participants, 4,105 were men (58.8%). Over a median follow-up of 2.4 years, 1,198 participants (17.2%) developed CP, corresponding to an incidence rate of 62.2 per 1,000 person-years. Compared with participants without CP, participants with incident CP were older and more likely to be male. They also exhibited a higher prevalence of cardiometabolic comorbidities, particularly diabetes, dyslipidemia, and hypertension. Anthropometric measures were consistently less favorable in the CP group, including higher BMI, WC, and WHtR, accompanied by elevated SBP and DBP. In terms of biochemical profiles, participants with incident CP showed higher levels of FPG, serum uric acid, serum creatinine, TC, TG, and LDL-C, along with lower HDL-C levels. All cardiometabolic indices were consistently elevated in the CP group, including RC, RC/HDL-C, non-HDL-C, CHG, TyG, AIP, AC, CRI-I, and CRI-II. Lifestyle factors, including smoking status and alcohol consumption, did not differ significantly between groups. SMDs indicated meaningful imbalances in several cardiometabolic and anthropometric variables (Table 1). Baseline characteristics of included and excluded participants are presented in Supplementary Table S7.

**Table 1:** Baseline characteristics of participants with and without carotid plaque.

Variable	Overall (*n* = 6,984)	Non-CP (*n* = 5,786)	CP (*n* = 1,198)	SMD
Age, y, median (IQR)	46.00 (37.00–52.00)	44.00 (36.00–51.00)	51.00 (46.00–57.00)	0.824
Sex, *n* (%)				0.438
Men	4,105 (58.8)	3,199 (55.3)	906 (75.6)	
Women	2,879 (41.2)	2,587 (44.7)	292 (24.4)	
Medication use, *n* (%)	669 (9.6)	455 (7.9)	214 (17.9)	0.302
Diabetes, *n* (%)	435 (6.2)	291 (5.0)	144 (12.0)	0.252
Dyslipidemia, *n* (%)	2,353 (33.7)	1,831 (31.6)	522 (43.6)	0.248
Hypertension, *n* (%)	1,621 (23.2)	1,189 (20.5)	432 (36.1)	0.350
Smoking status, *n* (%)	450 (6.4)	361 (6.2)	89 (7.4)	0.047
Alcohol consumption, *n* (%)	447 (6.4)	371 (6.4)	76 (6.3)	0.003
Height, cm, median (IQR)	170.00 (163.00–175.25)	169.00 (163.00–175.00)	171.00 (165.00–176.00)	0.031
Weight, kg, median (IQR)	71.00 (61.00–80.00)	70.00 (60.00–80.00)	74.50 (66.00–83.00)	0.326
BMI, kg/m^2^, median (IQR)	24.46 (22.20–26.81)	24.24 (21.95–26.57)	25.43 (23.44–27.63)	0.307
WC, cm, median (IQR)	86.00 (78.00–93.00)	85.00 (77.00–92.00)	90.00 (83.00–96.00)	0.440
WHtR, median (IQR)	0.50 (0.47–0.54)	0.50 (0.46–0.54)	0.52 (0.49–0.56)	0.424
SBP, mmHg, median (IQR)	125.00 (115.00–135.00)	124.00 (114.00–134.00)	132.00 (121.00–142.00)	0.476
DBP, mmHg, median (IQR)	77.00 (70.00–85.00)	76.00 (69.00–83.00)	82.00 (74.00–88.75)	0.438
RBC, × 10^12^/L, median (IQR)	4.86 (4.52–5.20)	4.84 (4.49–5.18)	4.98 (4.68–5.27)	0.265
WBC, × 10^9^/L, median (IQR)	5.78 (4.92–6.76)	5.75 (4.91–6.74)	5.95 (5.00–6.90)	0.134
Platelet count, × 10^9^/L, median (IQR)	233.00 (202.00–269.00)	234.00 (203.00–271.00)	228.00 (197.00–258.00)	0.154
Hemoglobin, g/L, median (IQR)	148.00 (136.00–158.00)	147.00 (134.00–158.00)	152.00 (143.00–161.00)	0.334
Neutrophil count, × 10^9^/L, median (IQR)	3.34 (2.71–4.11)	3.33 (2.69–4.08)	3.44 (2.81–4.27)	0.128
Lymphocyte count, × 10^9^/L, median (IQR)	1.91 (1.59–2.27)	1.91 (1.59–2.26)	1.93 (1.60–2.32)	0.048
Monocyte count, × 10^9^/L, median (IQR)	0.31 (0.24–0.39)	0.31 (0.24–0.39)	0.32 (0.25–0.41)	0.104
ALT, U/L, median (IQR)	20.38 (14.64–30.34)	20.00 (14.20–29.74)	22.94 (16.45–33.70)	0.131
AST, U/L, median (IQR)	20.31 (17.20–24.54)	20.03 (17.02–24.22)	21.44 (18.11–25.98)	0.148
Albumin, g/L, median (IQR)	46.05 (44.50–47.71)	46.09 (44.54–47.74)	45.89 (44.32–47.49)	0.106
FBG, mmol/L, median (IQR)	5.53 (5.24–5.91)	5.50 (5.22–5.86)	5.71 (5.39–6.18)	0.294
Serum uric acid, μmol/L, median (IQR)	356.07 (289.24–423.93)	350.89 (285.14–421.24)	374.02 (312.01–440.97)	0.220
Serum creatinine, μmol/L, median (IQR)	69.20 (57.95–80.21)	68.30 (57.22–79.82)	72.53 (62.80–82.05)	0.250
TC, mmol/L, median (IQR)	4.93 (4.38–5.56)	4.88 (4.34–5.51)	5.18 (4.60–5.77)	0.276
TG, mmol/L, median (IQR)	1.47 (1.06–2.05)	1.42 (1.02–1.98)	1.67 (1.22–2.34)	0.240
HDL-C, mmol/L, median (IQR)	1.28 (1.09–1.52)	1.29 (1.10–1.54)	1.23 (1.06–1.46)	0.205
LDL-C, mmol/L, median (IQR)	2.73 (2.26–3.24)	2.69 (2.24–3.21)	2.91 (2.46–3.40)	0.265
RC, median (IQR)	31.32 (24.36–39.93)	30.55 (23.59–39.06)	34.76 (26.68–43.70)	0.254
RC/HDL-C, median (IQR)	0.61 (0.44–0.87)	0.60 (0.43–0.84)	0.71 (0.52–0.98)	0.129
Non-HDL-C, mmol/L, median (IQR)	3.60 (3.05–4.23)	3.54 (3.01–4.16)	3.88 (3.30–4.50)	0.351
CHG, median (IQR)	5.26 (5.04–5.48)	5.24 (5.02–5.45)	5.39 (5.17–5.59)	0.436
TyG, median (IQR)	8.79 (8.44–9.15)	8.75 (8.40–9.11)	8.96 (8.63–9.33)	0.399
AIP, median (IQR)	0.05 (-0.14–0.24)	0.03 (-0.16–0.23)	0.12 (-0.05–0.31)	0.327
AC, median (IQR)	2.81 (2.15–3.61)	2.74 (2.10–3.51)	3.13 (2.42–3.96)	0.254
CRI-I, median (IQR)	3.81 (3.15–4.61)	3.74 (3.10–4.51)	4.13 (3.42–4.96)	0.254
CRI-II, median (IQR)	2.13 (1.64–2.71)	2.09 (1.61–2.66)	2.37 (1.84–2.95)	0.301

AC, atherogenic coefficient; AIP, atherogenic index of plasma; ALT, alanine aminotransferase; AST, aspartate aminotransferase; BMI, body mass index; CHG, cholesterol, high-density lipoprotein, and glucose index; CRI-I, Castelli's index-I; CRI-II, Castelli's index-II; DBP, diastolic blood pressure; FPG, fasting plasma glucose; HDL-C, high-density lipoprotein cholesterol; LDL-C, low-density lipoprotein cholesterol; Non-HDL-C, non-high density lipoprotein cholesterol; RBC, red blood cell count; RC, remnant cholesterol; RC/HDL-C, remnant cholesterol and high density lipoprotein cholesterol ratio; SBP, systolic blood pressure; SMD, standardized mean difference; TC, total cholesterol; TG, triglycerides; TyG, triglyceride-glucose index; WBC, white blood cell count; WC, waist circumference; CP, carotid plaque.

### 3.2 Associations of traditional lipid parameters, WHtR, and cardiometabolic indices with incident CP

In Model 3, TC and LDL-C were significantly associated with increased CP risk, with graded increases across quartiles (TC Q4 vs. Q1: HR 1.62, 95% CI 1.36–1.93; LDL-C Q4 vs. Q1: HR 1.68, 95% CI 1.42–1.99; both *P* for trend < 0.001). In contrast, WHtR was not independently associated with CP risk after full adjustment. Several cardiometabolic indices also showed positive associations with CP, particularly CRI-II and CHG (CRI-II Q4 vs. Q1: HR 1.86, 95% CI 1.54–2.25; CHG Q4 vs. Q1: HR 1.65, 95% CI 1.33–2.06; both *P* for trend < 0.001). Similar associations were observed in continuous and per 1-SD analyses, and K–M curves showed higher cumulative CP incidence in the upper quartiles (log-rank *P* < 0.001; Table 2 and Supplementary Figure S2). Given the observed associations of cardiometabolic indices and the lack of an independent association for WHtR, we further evaluated whether their combined effects could better characterize incident CP.

**Table 2:** Association of traditional lipid parameters, WHtR, and cardiometabolic indices with incident carotid plaque.

Exposure	Levels	Events	PY	Incidence rate_1000PY	Model 1		Model 2		Model 3	
					HR (95% CI)	*P*	HR (95% CI)	*P*	HR (95% CI)	*P*
TC	Continuous				1.23 (1.16–1.31)	< 0.001	1.23 (1.16–1.31)	< 0.001	1.19 (1.12–1.27)	< 0.001
	Per 1-SD				1.21 (1.15–1.28)	< 0.001	1.21 (1.15–1.28)	< 0.001	1.18 (1.11–1.25)	< 0.001
	Q1	207	5,083.263	40.722	Reference	—	Reference	—	Reference	—
	Q2	272	4,928.746	55.186	1.24 (1.04–1.49)	0.019	1.24 (1.03–1.49)	0.020	1.26 (1.05–1.51)	0.012
	Q3	314	4,633.911	67.761	1.41 (1.18–1.68)	< 0.001	1.41 (1.19–1.69)	< 0.001	1.43 (1.20–1.70)	< 0.001
	Q4	405	4,621.076	87.642	1.72 (1.46–2.04)	< 0.001	1.72 (1.46–2.04)	< 0.001	1.62 (1.36–1.93)	< 0.001
	*P* for trend					< 0.001		< 0.001		< 0.001
TG	Continuous				1.09 (1.05–1.13)	< 0.001	1.09 (1.05–1.13)	< 0.001	1.03 (0.98–1.08)	0.301
	Per 1-SD				1.10 (1.06–1.15)	< 0.001	1.10 (1.06–1.15)	< 0.001	1.03 (0.97–1.09)	0.301
	Q1	179	4,981.280	35.935	Reference	—	Reference	—	Reference	—
	Q2	299	4,853.712	61.602	1.41 (1.17–1.70)	< 0.001	1.41 (1.17–1.70)	< 0.001	1.36 (1.13–1.64)	0.001
	Q3	327	4,744.842	68.917	1.42 (1.18–1.71)	< 0.001	1.43 (1.18–1.71)	< 0.001	1.31 (1.08–1.58)	0.005
	Q4	393	4,687.163	83.846	1.60 (1.34–1.92)	< 0.001	1.60 (1.34–1.92)	< 0.001	1.25 (1.00–1.55)	0.046
	*P* for trend					< 0.001		< 0.001		0.083
HDL-C	Continuous				0.71 (0.59–0.87)	< 0.001	0.71 (0.58–0.87)	< 0.001	0.89 (0.72–1.09)	0.255
	Per 1-SD				0.89 (0.84–0.95)	< 0.001	0.89 (0.84–0.95)	< 0.001	0.96 (0.89–1.03)	0.255
	Q1	367	4,867.788	75.394	Reference	—	Reference	—	Reference	—
	Q2	313	4,782.955	65.441	0.93 (0.80–1.08)	0.346	0.93 (0.80–1.08)	0.338	1.09 (0.93–1.29)	0.281
	Q3	286	4,818.460	59.355	0.86 (0.73–1.01)	0.063	0.86 (0.73–1.01)	0.059	1.03 (0.86–1.22)	0.757
	Q4	232	4,797.794	48.356	0.80 (0.67–0.95)	0.012	0.80 (0.67–0.95)	0.011	0.97 (0.80–1.18)	0.794
	*P* for trend					0.007		0.007		0.680
LDL-C	Continuous				1.37 (1.28–1.47)	< 0.001	1.37 (1.28–1.48)	< 0.001	1.34 (1.24–1.44)	< 0.001
	Per 1-SD				1.27 (1.20–1.34)	< 0.001	1.27 (1.20–1.34)	< 0.001	1.24 (1.18–1.32)	< 0.001
	Q1	230	5,240.379	43.890	Reference	—	Reference	—	Reference	—
	Q2	251	5,138.274	48.849	1.02 (0.85–1.22)	0.808	1.02 (0.85–1.22)	0.820	1.06 (0.89–1.27)	0.520
	Q3	326	4,628.410	70.435	1.41 (1.19–1.67)	< 0.001	1.41 (1.19–1.67)	< 0.001	1.45 (1.22–1.71)	< 0.001
	Q4	391	4,259.934	91.785	1.73 (1.47–2.04)	< 0.001	1.73 (1.47–2.04)	< 0.001	1.68 (1.42–1.99)	< 0.001
	*P* for trend					< 0.001		< 0.001		< 0.001
WHtR	Continuous				8.86 (2.98–26.36)	< 0.001	8.87 (2.98–26.41)	< 0.001	2.19 (0.66–7.22)	0.197
	Per 1-SD				1.13 (1.06–1.21)	< 0.001	1.13 (1.06–1.21)	< 0.001	1.05 (0.98–1.12)	0.197
	Q1	148	4,755.911	31.119	Reference	—	Reference	—	Reference	—
	Q2	258	4,872.748	52.948	1.10 (0.89–1.35)	0.386	1.10 (0.89–1.35)	0.390	1.05 (0.85–1.29)	0.643
	Q3	360	4,799.974	75.000	1.26 (1.03–1.54)	0.024	1.26 (1.03–1.54)	0.025	1.14 (0.93–1.40)	0.196
	Q4	432	4,838.364	89.286	1.40 (1.15–1.71)	< 0.001	1.40 (1.15–1.71)	< 0.001	1.16 (0.95–1.43)	0.153
	*P* for trend					< 0.001		< 0.001		0.100
RC	Continuous				1.00 (1.00–1.01)	0.057	1.00 (1.00–1.01)	0.057	1.00 (0.99–1.00)	0.307
	Per 1-SD				1.05 (1.00–1.11)	0.057	1.05 (1.00–1.11)	0.057	0.97 (0.91–1.03)	0.307
	Q1	225	4,585.602	49.067	Reference	—	Reference	—	Reference	—
	Q2	240	4,556.095	52.677	0.89 (0.74–1.07)	0.211	0.89 (0.74–1.07)	0.214	0.86 (0.72–1.04)	0.122
	Q3	322	4,932.759	65.278	0.92 (0.78–1.09)	0.348	0.92 (0.77–1.09)	0.335	0.84 (0.70–1.00)	0.050
	Q4	411	5,192.541	79.152	1.09 (0.93–1.29)	0.301	1.09 (0.93–1.29)	0.301	0.87 (0.73–1.05)	0.156
	*P* for trend					0.129		0.131		0.193
RC/HDL-C	Continuous				1.04 (0.99–1.09)	0.108	1.04 (0.99–1.09)	0.11	0.97 (0.89–1.06)	0.507
	Per 1-SD				1.04 (0.99–1.09)	0.108	1.04 (0.99–1.09)	0.110	0.97 (0.89–1.06)	0.507
	Q1	186	4,398.172	42.290	Reference	—	Reference	—	Reference	—
	Q2	277	4,805.467	57.643	1.07 (0.89–1.29)	0.494	1.07 (0.88–1.29)	0.499	1.01 (0.84–1.22)	0.905
	Q3	332	4,977.239	66.704	1.03 (0.86–1.24)	0.715	1.03 (0.86–1.24)	0.721	0.92 (0.76–1.11)	0.387
	Q4	403	5,086.119	79.235	1.21 (1.01–1.44)	0.040	1.21 (1.01–1.44)	0.040	0.88 (0.71–1.09)	0.239
	*P* for trend					0.042		0.041		0.145
Non-HDL-C	Continuous				1.28 (1.20–1.36)	< 0.001	1.28 (1.20–1.36)	< 0.001	1.24 (1.16–1.33)	< 0.001
	Per 1-SD				1.25 (1.18–1.32)	< 0.001	1.25 (1.18–1.32)	< 0.001	1.22 (1.15–1.30)	< 0.001
	Q1	199	5,109.680	38.946	Reference	—	Reference	—	Reference	—
	Q2	240	4,870.600	49.275	1.09 (0.90–1.31)	0.392	1.08 (0.90–1.31)	0.404	1.08 (0.90–1.31)	0.401
	Q3	345	4,781.642	72.151	1.48 (1.24–1.77)	< 0.001	1.48 (1.24–1.77)	< 0.001	1.45 (1.22–1.74)	< 0.001
	Q4	414	4,505.075	91.896	1.74 (1.47–2.06)	< 0.001	1.74 (1.47–2.06)	< 0.001	1.61 (1.34–1.93)	< 0.001
	*P* for trend					< 0.001		< 0.001		< 0.001
CHG	Continuous				1.84 (1.58–2.14)	< 0.001	1.84 (1.58–2.15)	< 0.001	1.61 (1.32–1.97)	< 0.001
	Per 1-SD				1.24 (1.18–1.31)	< 0.001	1.24 (1.18–1.31)	< 0.001	1.19 (1.11–1.27)	< 0.001
	Q1	164	4,983.029	32.912	Reference	—	Reference	—	Reference	—
	Q2	256	4,893.996	52.309	1.25 (1.02–1.52)	0.028	1.25 (1.03–1.53)	0.026	1.23 (1.01–1.50)	0.042
	Q3	328	4,897.236	66.977	1.44 (1.19–1.74)	< 0.001	1.44 (1.19–1.75)	< 0.001	1.38 (1.13–1.68)	0.002
	Q4	450	4,492.736	100.162	1.89 (1.56–2.27)	< 0.001	1.89 (1.57–2.28)	< 0.001	1.65 (1.33–2.06)	< 0.001
	*P* for trend					< 0.001		< 0.001		< 0.001
TyG	Continuous				1.36 (1.23–1.50)	< 0.001	1.36 (1.23–1.50)	< 0.001	1.18 (1.03–1.35)	0.013
	Per 1-SD				1.20 (1.13–1.27)	< 0.001	1.20 (1.13–1.27)	< 0.001	1.10 (1.02–1.19)	0.013
	Q1	162	4,988.373	32.476	Reference	—	Reference	—	Reference	—
	Q2	275	4,792.151	57.386	1.37 (1.12–1.66)	0.002	1.37 (1.12–1.66)	0.002	1.31 (1.07–1.59)	0.007
	Q3	337	4,784.762	70.432	1.48 (1.22–1.79)	< 0.001	1.48 (1.22–1.79)	< 0.001	1.36 (1.12–1.65)	0.002
	Q4	424	4,701.711	90.180	1.73 (1.43–2.08)	< 0.001	1.73 (1.43–2.08)	< 0.001	1.36 (1.09–1.71)	0.007
	*P* for trend					< 0.001		< 0.001		0.009
AIP	Continuous				1.73 (1.42–2.11)	< 0.001	1.73 (1.42–2.12)	< 0.001	1.32 (1.01–1.72)	0.040
	Per 1-SD				1.18 (1.11–1.25)	< 0.001	1.18 (1.11–1.25)	< 0.001	1.09 (1.00–1.17)	0.040
	Q1	184	4,914.416	37.441	Reference	—	Reference	—	Reference	—
	Q2	278	4,818.920	57.689	1.29 (1.07–1.56)	0.007	1.29 (1.07–1.56)	0.007	1.26 (1.05–1.52)	0.015
	Q3	342	4,777.387	71.587	1.42 (1.18–1.70)	< 0.001	1.42 (1.18–1.71)	< 0.001	1.30 (1.08–1.56)	0.007
	Q4	394	4,756.274	82.838	1.58 (1.32–1.89)	< 0.001	1.58 (1.32–1.90)	< 0.001	1.22 (0.98–1.53)	0.079
	*P* for trend					< 0.001		< 0.001		0.064
AC	Continuous				1.06 (1.04–1.08)	< 0.001	1.06 (1.04–1.08)	< 0.001	1.05 (1.02–1.07)	< 0.001
	Per 1-SD				1.09 (1.07–1.12)	< 0.001	1.09 (1.07–1.13)	< 0.001	1.07 (1.03–1.12)	< 0.001
	Q1	193	4,933.417	39.121	Reference	—	Reference	—	Reference	—
	Q2	251	4,952.340	50.683	1.08 (0.90–1.31)	0.403	1.09 (0.90–1.31)	0.392	1.07 (0.89–1.30)	0.467
	Q3	335	4,836.783	69.261	1.43 (1.19–1.71)	< 0.001	1.43 (1.19–1.71)	< 0.001	1.38 (1.15–1.67)	< 0.001
	Q4	419	4,544.457	92.200	1.74 (1.46–2.07)	< 0.001	1.74 (1.46–2.08)	< 0.001	1.62 (1.32–1.99)	< 0.001
	*P* for trend					< 0.001		< 0.001		< 0.001
CRI-I	Continuous				1.06 (1.04–1.08)	< 0.001	1.06 (1.04–1.08)	< 0.001	1.05 (1.02–1.07)	< 0.001
	Per 1-SD				1.09 (1.07–1.12)	< 0.001	1.09 (1.07–1.13)	< 0.001	1.07 (1.03–1.12)	< 0.001
	Q1	193	4,933.417	39.121	Reference	—	Reference	—	Reference	—
	Q2	251	4,952.340	50.683	1.08 (0.90–1.31)	0.403	1.09 (0.90–1.31)	0.392	1.07 (0.89–1.30)	0.467
	Q3	335	4,836.783	69.261	1.43 (1.19–1.71)	< 0.001	1.43 (1.19–1.71)	< 0.001	1.38 (1.15–1.67)	< 0.001
	Q4	419	4,544.457	92.200	1.74 (1.46–2.07)	< 0.001	1.74 (1.46–2.08)	< 0.001	1.62 (1.32–1.99)	< 0.001
	*P* for trend					< 0.001		< 0.001		< 0.001
CRI-II	Continuous				1.27 (1.21–1.33)	< 0.001	1.27 (1.21–1.33)	< 0.001	1.24 (1.17–1.31)	< 0.001
	Per 1-SD				1.22 (1.17–1.27)	< 0.001	1.22 (1.17–1.27)	< 0.001	1.20 (1.14–1.26)	< 0.001
	Q1	197	5,076.285	38.808	Reference	—	Reference	—	Reference	—
	Q2	272	5,036.474	54.006	1.17 (0.97–1.40)	0.101	1.17 (0.97–1.41)	0.097	1.17 (0.98–1.41)	0.089
	Q3	314	4,853.996	64.689	1.39 (1.16–1.67)	< 0.001	1.40 (1.17–1.67)	< 0.001	1.38 (1.15–1.66)	< 0.001
	Q4	415	4,300.242	96.506	1.94 (1.63–2.32)	< 0.001	1.95 (1.64–2.33)	< 0.001	1.86 (1.54–2.25)	< 0.001
	*P* for trend					< 0.001		< 0.001		< 0.001

PY, person-years; Incidence rates_1000PY are expressed per 1,000 PY. Age was modeled using restricted cubic splines with 4 knots. Model 1 is adjusted for age and sex; Model 2 is adjusted for age, sex, smoking status, and alcohol consumption; and Model 3 is adjusted for age, sex, smoking status, alcohol consumption, diabetes, dyslipidemia, hypertension, platelet count, and monocyte count. AC, atherogenic coefficient; AIP, atherogenic index of plasma; CHG, cholesterol, high-density lipoprotein, and glucose index; CI, confidence interval; CRI-I, Castelli's index-I; CRI-II, Castelli's index-II; HDL-C, high-density lipoprotein cholesterol; HR, hazard ratio; LDL-C, low-density lipoprotein cholesterol; Non-HDL-C, non-high density lipoprotein cholesterol; RC, remnant cholesterol; RC/HDL-C, remnant cholesterol and high-density lipoprotein cholesterol ratio; TC, total cholesterol; TG, triglycerides; TyG, triglyceride-glucose index; WHtR, waist-to-height ratio.

### 3.3 Product-based and additive associations of cardiometabolic indices with incident CP

**Figure 2:**
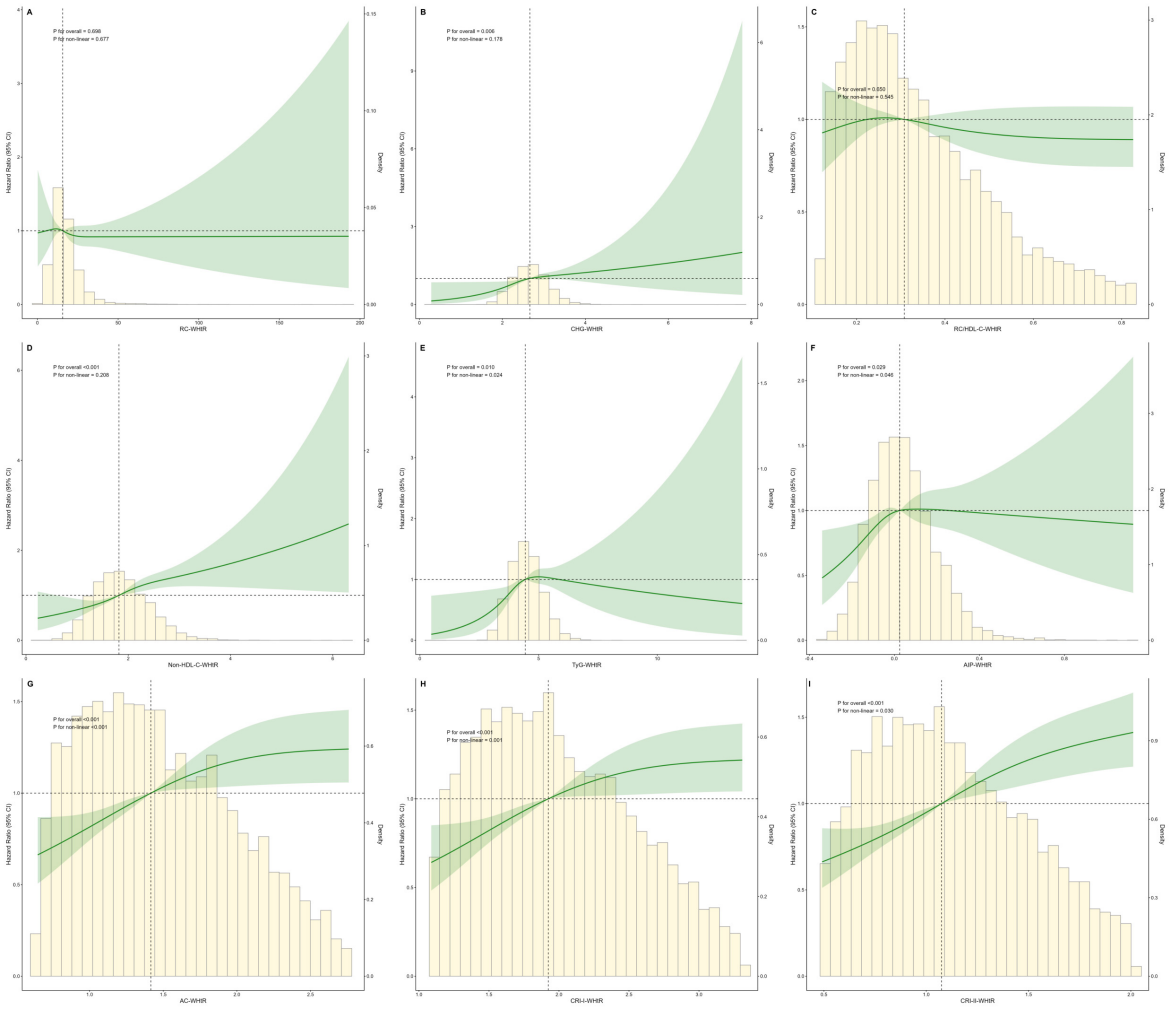
Dose–response relationships between cardiometabolic index–WHtR composites and incident carotid plaque. Models were adjusted for age, sex, smoking status, alcohol consumption, diabetes, dyslipidemia, hypertension, platelet count, and monocyte count. Age was modeled using restricted cubic splines with 4 knots. **(A)** RC–WHtR; **(B)** RC/HDL-C–WHtR **(C)** Non-HDL-C–WHtR; **(D)** CHG–WHtR; **(E)** TyG–WHtR; **(F)** AIP–WHtR; **(G)** AC–WHtR; **(H)** CRI-I–WHtR; **(I)** CRI-II–WHtR. Multiplicative composite indices are constructed by multiplying each cardiometabolic index and WHtR. AC, atherogenic coefficient; AIP, atherogenic index of plasma; CHG, cholesterol, high-density lipoprotein, and glucose index; CI, confidence interval; CRI-I, Castelli's index-I; CRI-II, Castelli's index-II; HR, hazard ratio; Non-HDL-C, non-high density lipoprotein cholesterol; RC, remnant cholesterol; RC/HDL-C, remnant cholesterol and high density lipoprotein cholesterol ratio; TyG, triglyceride-glucose index; WHtR, waist-to-height ratio.

In Model 3, the product-based composite indices showed significant associations with incident CP, with graded increases in risk across quartiles. Among these indices, CRI-II–WHtR and non-HDL-C–WHtR were associated with higher CP risk in the highest quartile compared with the lowest quartile (CRI-II–WHtR: HR 2.00, 95% CI 1.63–2.46; non-HDL-C–WHtR: HR 1.76, 95% CI 1.44–2.15; both *P* for trend < 0.001), with similar associations observed in continuous and per 1-SD analyses (Table 3). In supplementary analyses, additive composite models (index + WHtR) also showed positive associations with incident CP. In Model 3, CRI-II + WHtR and non-HDL-C + WHtR were associated with increased CP risk in Q4 vs. Q1 (CRI-II + WHtR: HR 1.72, 95% CI 1.38–2.14; non-HDL-C + WHtR: HR 1.61, 95% CI 1.30–1.99; both *P* for trend < 0.001; Supplementary Table S8). Product-based indices showed larger effect estimates than the corresponding additive composites. Sex-stratified analyses showed similar associations. In males, non-HDL-C–WHtR and CRI-II–WHtR were associated with incident CP (Q4 vs. Q1: HR 1.68, 95% CI 1.36–2.06; HR 1.65, 95% CI 1.34–2.05; both *P* for trend < 0.001; Supplementary Table S9). In females, the corresponding HRs were 1.93 (95% CI 1.25–2.99) and 1.70 (95% CI 1.16–2.50), both *P* for trend < 0.001, respectively (Supplementary Table S10). RCS analyses indicated non-linear associations for TyG–WHtR, AIP–WHtR, AC–WHtR, CRI-I–WHtR, and CRI-II–WHtR, whereas CHG–WHtR and non-HDL-C–WHtR showed approximately linear associations (Figure 2). We then assessed interactions with WHtR and conducted subgroup analyses to explore potential effect modification.

**Table 3:** Association of multiplicative cardiometabolic–WHtR composite indices with incident carotid plaque.

Exposure	Levels	Events	PY	Incidence rate_1000PY	Model 1		Model 2		Model 3	
					HR (95% CI)	*P*	HR (95% CI)	*P*	HR (95% CI)	*P*
RC–WHtR	Continuous				1.01 (1.00–1.01)	0.011	1.01 (1.00–1.01)	0.011	1.00 (0.99–1.00)	0.488
	Per 1-SD				1.07 (1.02–1.13)	0.011	1.07 (1.02–1.13)	0.011	0.98 (0.92–1.04)	0.488
	Q1	178	4,355.638	40.867	Reference	—	Reference	—	Reference	—
	Q2	254	4,719.432	53.82	1.01 (0.84–1.23)	0.893	1.01 (0.83–1.23)	0.903	0.97 (0.80–1.17)	0.738
	Q3	331	5,016.556	65.982	1.02 (0.85–1.23)	0.830	1.02 (0.85–1.23)	0.843	0.91 (0.76–1.10)	0.354
	Q4	435	5,175.371	84.052	1.21 (1.01–1.44)	0.040	1.20 (1.01–1.44)	0.041	0.95 (0.78–1.16)	0.600
	*P* for trend					0.017		0.018		0.550
RC/HDL-C–WHtR	Continuous				1.08 (0.99–1.17)	0.068	1.08 (0.99–1.17)	0.068	0.95 (0.82–1.11)	0.545
	Per 1-SD				1.04 (1.00–1.09)	0.068	1.04 (1.00–1.09)	0.068	0.98 (0.90–1.06)	0.545
	Q1	165	4,397.494	37.521	Reference	—	Reference	—	Reference	—
	Q2	285	4,845.08	58.823	1.15 (0.95–1.40)	0.155	1.15 (0.95–1.40)	0.155	1.10 (0.90–1.33)	0.358
	Q3	334	4,966.549	67.25	1.09 (0.90–1.32)	0.378	1.09 (0.90–1.32)	0.383	0.95 (0.78–1.16)	0.649
	Q4	414	5,057.874	81.853	1.29 (1.07–1.55)	0.008	1.29 (1.07–1.55)	0.008	0.93 (0.75–1.17)	0.549
	*P* for trend					0.013		0.013		0.259
Non-HDL-C–WHtR	Continuous				1.55 (1.40–1.71)	< 0.001	1.55 (1.40–1.71)	< 0.001	1.44 (1.28–1.62)	< 0.001
	Per 1-SD				1.27 (1.20–1.34)	< 0.001	1.27 (1.21–1.35)	< 0.001	1.22 (1.15–1.30)	< 0.001
	Q1	162	4,947.165	32.746	Reference	—	Reference	—	Reference	—
	Q2	244	4,961.411	49.18	1.15 (0.94–1.40)	0.171	1.15 (0.94–1.40)	0.175	1.14 (0.93–1.39)	0.199
	Q3	350	4,848.806	72.183	1.48 (1.22–1.78)	< 0.001	1.48 (1.22–1.78)	< 0.001	1.42 (1.17–1.72)	< 0.001
	Q4	442	4,509.616	98.013	1.91 (1.59–2.29)	< 0.001	1.91 (1.59–2.29)	< 0.001	1.76 (1.44–2.15)	< 0.001
	*P* for trend					< 0.001		< 0.001		< 0.001
CHG–WHtR	Continuous				1.70 (1.47–1.97)	< 0.001	1.71 (1.47–1.97)	< 0.001	1.37 (1.15–1.64)	< 0.001
	Per 1-SD				1.25 (1.18–1.33)	< 0.001	1.25 (1.18–1.33)	< 0.001	1.14 (1.06–1.23)	< 0.001
	Q1	132	4,783.908	27.593	Reference	—	Reference	—	Reference	—
	Q2	271	5,016.098	54.026	1.29 (1.04–1.59)	0.020	1.29 (1.04–1.59)	0.021	1.23 (1.00–1.53)	0.055
	Q3	342	4,815.265	71.024	1.42 (1.15–1.76)	0.001	1.43 (1.15–1.76)	0.001	1.30 (1.05–1.62)	0.017
	Q4	453	4,651.727	97.383	1.85 (1.51–2.29)	< 0.001	1.85 (1.50–2.29)	< 0.001	1.48 (1.18–1.86)	< 0.001
	*P* for trend					< 0.001		< 0.001		< 0.001
TyG–WHtR	Continuous				1.33 (1.22–1.46)	< 0.001	1.33 (1.22–1.46)	< 0.001	1.15 (1.03–1.28)	0.014
	Per 1-SD				1.22 (1.15–1.30)	< 0.001	1.22 (1.15–1.30)	< 0.001	1.10 (1.02–1.19)	0.014
	Q1	121	4,813.550	25.137	Reference	—	Reference	—	Reference	—
	Q2	271	4,888.054	55.441	1.49 (1.20–1.86)	< 0.001	1.49 (1.20–1.86)	< 0.001	1.44 (1.15–1.79)	0.001
	Q3	365	4,846.662	75.31	1.63 (1.31–2.02)	< 0.001	1.63 (1.31–2.02)	< 0.001	1.49 (1.19–1.85)	< 0.001
	Q4	441	4,718.731	93.457	1.97 (1.59–2.44)	< 0.001	1.97 (1.59–2.44)	< 0.001	1.56 (1.23–1.97)	< 0.001
	*P* for trend					< 0.001		< 0.001		0.002
AIP–WHtR	Continuous				2.68 (1.85–3.90)	< 0.001	2.69 (1.85–3.92)	< 0.001	1.52 (0.93–2.51)	0.098
	Per 1-SD				1.16 (1.10–1.23)	< 0.001	1.16 (1.10–1.23)	< 0.001	1.07 (0.99–1.15)	0.098
	Q1	191	4,932.912	38.720	Reference	—	Reference	—	Reference	—
	Q2	269	4,797.621	56.069	1.28 (1.06–1.54)	0.009	1.28 (1.06–1.54)	0.009	1.26 (1.04–1.51)	0.017
	Q3	332	4,795.549	69.231	1.35 (1.13–1.62)	0.001	1.36 (1.13–1.63)	< 0.001	1.26 (1.05–1.52)	0.015
	Q4	406	4,740.915	85.637	1.58 (1.33–1.89)	< 0.001	1.59 (1.33–1.90)	< 0.001	1.25 (1.00–1.56)	0.047
	*P* for trend					< 0.001		< 0.001		0.048
AC–WHtR	Continuous				1.12 (1.08–1.15)	< 0.001	1.12 (1.08–1.15)	< 0.001	1.09 (1.04–1.14)	< 0.001
	Per 1-SD				1.10 (1.07–1.13)	< 0.001	1.10 (1.07–1.13)	< 0.001	1.08 (1.03–1.12)	< 0.001
	Q1	163	4,911.139	33.190	Reference	—	Reference	—	Reference	—
	Q2	275	4,934.674	55.728	1.29 (1.06–1.57)	0.012	1.29 (1.06–1.57)	0.011	1.25 (1.03–1.53)	0.024
	Q3	335	4,883.086	68.604	1.43 (1.18–1.73)	< 0.001	1.43 (1.18–1.73)	< 0.001	1.36 (1.12–1.67)	0.002
	Q4	425	4,538.099	93.652	1.89 (1.56–2.28)	< 0.001	1.90 (1.57–2.29)	< 0.001	1.68 (1.35–2.09)	< 0.001
	*P* for trend					< 0.001		< 0.001		< 0.001
CRI-I–WHtR	Continuous				1.12 (1.08–1.15)	< 0.001	1.12 (1.08–1.15)	< 0.001	1.09 (1.04–1.14)	< 0.001
	Per 1-SD				1.11 (1.07–1.14)	< 0.001	1.11 (1.07–1.14)	< 0.001	1.08 (1.04–1.12)	< 0.001
	Q1	161	4,873.872	33.033	Reference	—	Reference	—	Reference	—
	Q2	270	4,947.911	54.568	1.23 (1.01–1.50)	0.038	1.24 (1.01–1.51)	0.036	1.20 (0.98–1.46)	0.078
	Q3	332	4,905.278	67.682	1.35 (1.11–1.64)	0.003	1.35 (1.11–1.64)	0.003	1.29 (1.05–1.58)	0.015
	Q4	435	4,539.936	95.816	1.89 (1.56–2.28)	< 0.001	1.89 (1.56–2.29)	< 0.001	1.67 (1.34–2.09)	< 0.001
	*P* for trend					< 0.001		< 0.001		< 0.001
CRI-II–WHtR	Continuous				1.54 (1.41–1.68)	< 0.001	1.54 (1.41–1.69)	< 0.001	1.46 (1.30–1.62)	< 0.001
	Per 1-SD				1.24 (1.19–1.30)	< 0.001	1.24 (1.19–1.30)	< 0.001	1.21 (1.14–1.27)	< 0.001
	Q1	165	5,036.563	32.760	Reference	—	Reference	—	Reference	—
	Q2	290	5,022.393	57.741	1.40 (1.15–1.70)	< 0.001	1.40 (1.15–1.70)	< 0.001	1.37 (1.13–1.67)	0.001
	Q3	313	4,842.695	64.633	1.46 (1.20–1.77)	< 0.001	1.46 (1.21–1.77)	< 0.001	1.44 (1.18–1.75)	< 0.001
	Q4	430	4,365.346	98.503	2.16 (1.79–2.60)	< 0.001	2.17 (1.80–2.62)	< 0.001	2.00 (1.63–2.46)	< 0.001
	*P* for trend					< 0.001		< 0.001		< 0.001

PY, person-years; Incidence rates_1000PY are expressed per 1,000 PY. Age was modeled using restricted cubic splines with 4 knots. Model 1 is adjusted for age and sex; Model 2 is adjusted for age, sex, smoking status, and alcohol consumption; and Model 3 is adjusted for age, sex, smoking status, alcohol consumption, diabetes, dyslipidemia, hypertension, platelet count, and monocyte count. Multiplicative composite indices are constructed by multiplying each cardiometabolic index and WHtR. AC, atherogenic coefficient; AIP, atherogenic index of plasma; CHG, cholesterol, high-density lipoprotein, and glucose index; CI, confidence interval; CRI-I, Castelli's index-I; CRI-II, Castelli's index-II; HR, hazard ratio; Non-HDL-C, non-high density lipoprotein cholesterol; RC, remnant cholesterol; RC/HDL-C, remnant cholesterol and high density lipoprotein cholesterol ratio; TyG, triglyceride-glucose index; WHtR, waist-to-height ratio.

### 3.4 Interaction and subgroup analyses

**Figure 3:**
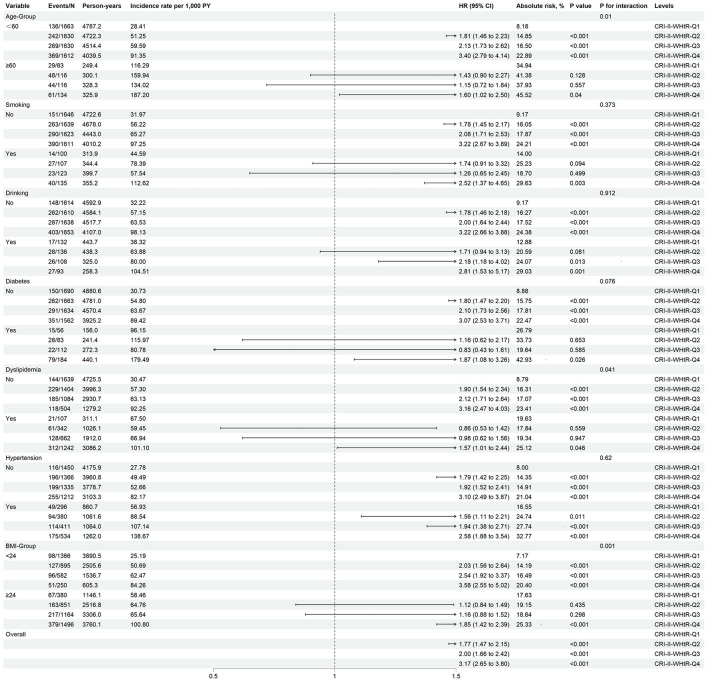
Subgroup analyses of the association between CRI-II–WHtR and incident carotid plaque. PY, person-years; CRI-II–WHtR is constructed by multiplying CRI-II and WHtR. CRI-II, Castelli's index-II; HR, hazard ratio; WHtR, waist-to-height ratio.

In Model 3, significant interactions with WHtR were observed for TyG, AIP, and CRI-II, with *P* for interaction values of 0.016, 0.020, and 0.019, respectively (Supplementary Table S11). Given its pronounced association, further subgroup analyses of CRI-II–WHtR showed significant effect modification by age, dyslipidemia, and BMI category, with *P* for interaction values of 0.010, 0.041, and 0.001, respectively. No significant interactions were observed across smoking status, alcohol consumption, diabetes, or hypertension strata. Detailed results, including event numbers, person-years, incidence rates, subgroup-specific HRs, absolute risks, and *P* values for interaction, are presented in Figure 3.

### 3.5 Time-dependent and time-weighted exposure analyses

Time-dependent Cox models using updated exposures showed associations largely consistent with the baseline findings, although effect estimates were modestly attenuated after full adjustment. Among the WHtR-based product indices, CRI-II–WHtR and non-HDL-C–WHtR remained significantly associated with incident CP, with higher risks in Q4 vs. Q1 (CRI-II–WHtR: HR 1.72, 95% CI 1.38–2.15; non-HDL-C–WHtR: HR 1.58, 95% CI 1.29–1.94; both *P* for trend < 0.001; Supplementary Table S12). To further capture early cumulative exposure, TWAvg indices were examined and showed similar associations, with numerically larger effect estimates than the time-dependent models. The highest quartiles of TWAvg CRI-II–WHtR and TWAvg non-HDL-C–WHtR were associated with increased CP risk (HR 1.91, 95% CI 1.55–2.37; HR 1.71, 95% CI 1.39–2.12; both *P* for trend < 0.001; Supplementary Table S13). Overall, these findings support the consistency of CRI-II–WHtR and non-HDL-C–WHtR across baseline, updated, and early cumulative exposure analyses.

### 3.6 Predictive performance and incremental value

**Figure 4:**
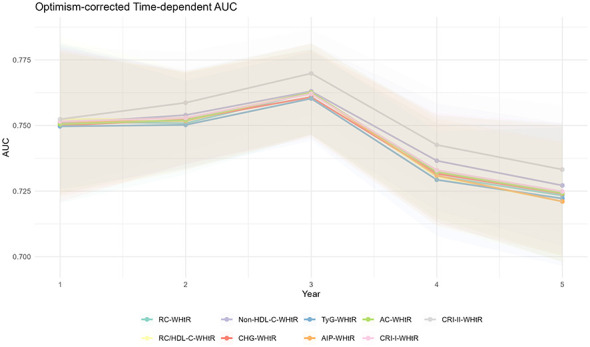
Time-dependent receiver operating characteristic curves of WHtR-based product indices for carotid plaque prediction. Models were adjusted for age, sex, smoking status, alcohol consumption, diabetes, dyslipidemia, hypertension, platelet count, and monocyte count. Multiplicative composite indices are constructed by multiplying each cardiometabolic index and WHtR. AC, atherogenic coefficient; AIP, atherogenic index of plasma; CHG, cholesterol, high-density lipoprotein, and glucose index; CRI-I, Castelli's index-I; CRI-II, Castelli's index-II; Non-HDL-C, non-high density lipoprotein cholesterol; RC, remnant cholesterol; RC/HDL-C, remnant cholesterol and high density lipoprotein cholesterol ratio; TyG, triglyceride-glucose index; WHtR, waist-to-height ratio.

**Figure 5:**
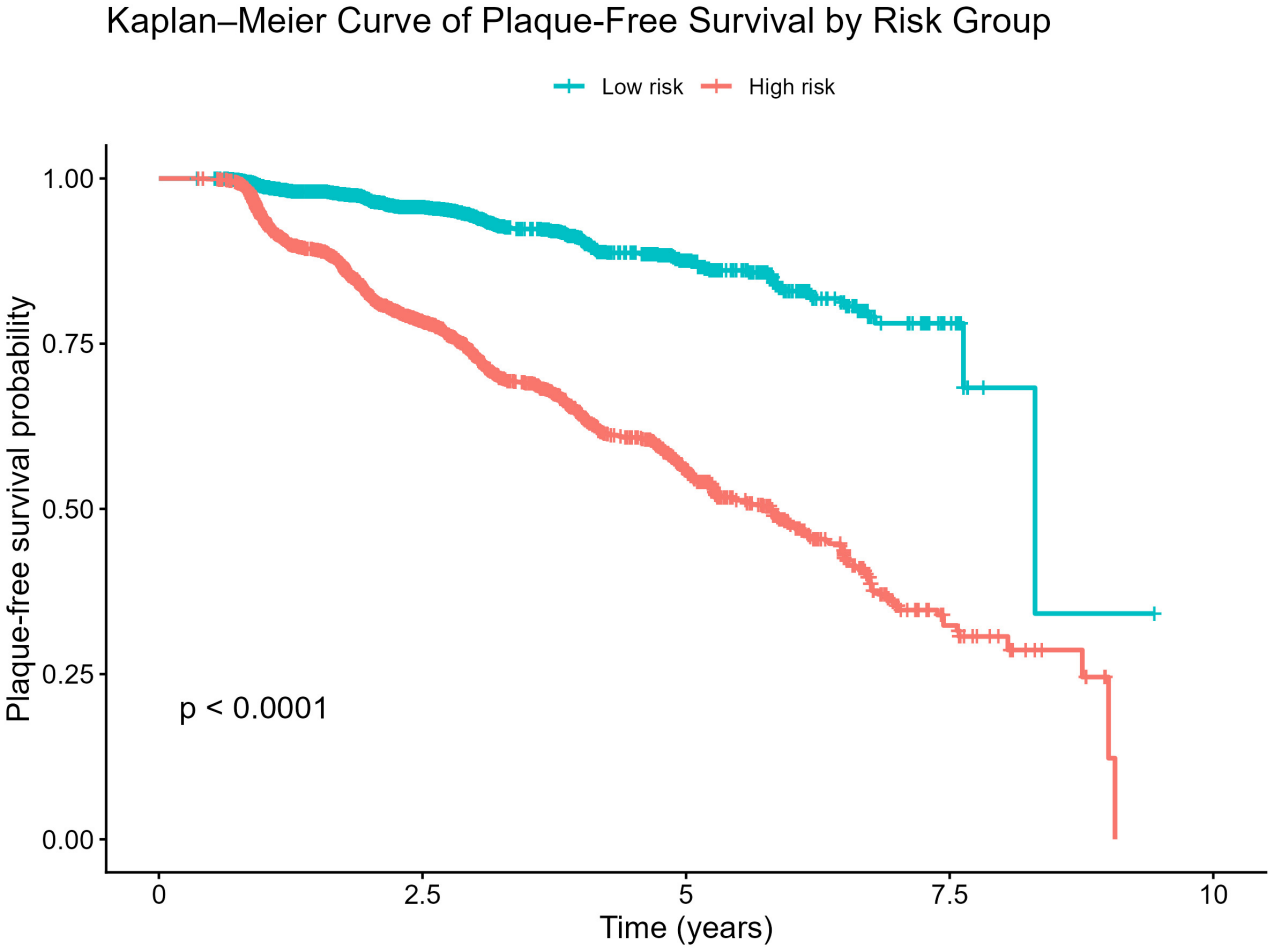
Kaplan–Meier curves for incident carotid plaque stratified by predicted 3-year risk based on CRI-II–WHtR. CRI-II–WHtR is constructed by multiplying CRI-II and WHtR. CRI-II, Castelli's index-II; WHtR, waist-to-height ratio.

Time-dependent ROC analyses showed moderate discrimination for incident CP across all WHtR-based product indices over 5 years. Optimism-corrected AUCs ranged from 0.750–0.752 at 1 year, peaked at 3 years (0.760–0.770), and declined to 0.721–0.733 at 5 years, with CRI-II–WHtR showing the highest AUC (0.770, 95% CI 0.754–0.787) at 3 years (Supplementary Table S14; Figure 4). This pattern was confirmed by C-index analyses, in which Harrell's C-index ranged from 0.733 to 0.739, with the highest value for CRI-II–WHtR (0.739, 95% CI 0.726–0.753), whereas Uno's C-index was 0.673 (95% CI 0.669–0.685) (Supplementary Table S15). Model performance was broadly comparable across indices beyond discrimination. Calibration curves at 3 years showed good agreement between predicted and observed risks, and DCA indicated similar net clinical benefit across threshold probabilities, without clear dominance of a single model (Supplementary Figures S3, S4). Time-dependent Brier scores increased gradually during follow-up, while IBS values were nearly identical across indices (0.098–0.099), suggesting comparable overall prediction error (Supplementary Figure S5; Supplementary Table S15). The incremental value of CRI-II–WHtR was further assessed by adding it to the baseline model. This addition yielded statistically significant but modest improvement in 3-year reclassification, mainly among non-cases (NRI for non-cases: 0.119, 95% CI 0.059–0.150), whereas improvement among cases was minimal (NRI for cases: 0.005, 95% CI 0.002–0.010). The IDI was also significant but small (0.003, 95% CI 0.001–0.005). Given the tendency of category-free NRI to overestimate improvement, these results should be interpreted cautiously. Finally, K–M curves showed significantly lower plaque-free survival in the high-risk CRI-II–WHtR group than in the low-risk group (log-rank *P* < 0.0001; Figure 5), supporting its ability to stratify CP risk.

### 3.7 Sensitivity analyses

Sensitivity analyses supported the robustness of the primary findings. Further adjustment for medication use, liver and renal function markers, and other potential confounders did not materially alter the estimates, with CRI-II–WHtR consistently showing pronounced associations. Excluding participants who developed CP within 2 years produced similar results, suggesting a limited influence of reverse causation. Complete-case analyses were also consistent with the primary analyses (Supplementary Tables S16–S18).

### 3.8 Exploratory mediation analysis

Exploratory mediation analyses were performed to examine the relationships among cardiometabolic indices, WHtR, and incident CP. WHtR accounted for 20.23%−63.58% of the observed association between cardiometabolic indices and CP. These findings suggest that central adiposity may contribute to the association between cardiometabolic abnormalities and CP, but should be interpreted as exploratory rather than causal evidence (Supplementary Figure S6).

## 4 Discussion

This retrospective cohort study systematically compared the joint effects of multiple cardiometabolic indices and WHtR within the same population, addressing the lack of unified comparisons of their associations with CP in previous studies. The primary exposure was defined as WHtR-based multiplicative composite indices, with CRI-II–WHtR as the key index of interest. The primary analysis compared multiplicative, additive, and interaction-based approaches using Cox proportional hazards models for incident CP. In these models, associations were also expressed per 1-standard deviation (1-SD) increase in each exposure to assess the robustness of the results and ensure comparability across indices. Multiplicative integration consistently showed stronger associations than additive approaches. Interaction analyses statistically suggested a combined effect between metabolic abnormalities and central obesity, which has not been adequately evaluated in previous studies. Among the indices, the CRI-II–WHtR multiplicative index showed the more pronounced association with CP, with consistent findings across both sexes and remained robust after standardization. Secondary analyses included the application of time-dependent and time-weighted models to reduce regression dilution bias. In addition, dose–response analyses, sensitivity analyses, and predictive performance assessments (including discrimination, calibration, DCA, and K–M curves) were conducted to evaluate the robustness of the findings. Subgroup and mediation analyses were conducted as exploratory analyses, and their results should be interpreted with caution. Although CRI-II–WHtR provided only modest improvement in model discrimination (category-free NRI: non-cases 0.119, cases 0.005; IDI: 0.003), differences across risk strata should be interpreted cautiously. Overall, these findings provide additional epidemiological evidence supporting WHtR-based multiplicative composite indices, particularly CRI-II–WHtR, for early risk characterization of CP; however, their clinical utility requires further validation in independent populations.

When constructing product-based composite indices, this study did not standardize the original variables, a strategy that may be methodologically and clinically justified. Retaining the original scales preserves the biological and physical meaning of the indices. Product-type indices capture the combined cumulative effects of multiple dimensions of metabolic abnormalities, whereas standardization may weaken the intuitive representation of actual risk magnitude. Previous studies developing composite indices such as CHG-BMI have likewise integrated variables on their original scales to comprehensively characterize glucolipotoxicity (27). From a statistical perspective, product terms are commonly used to capture interaction effects between variables, and their interpretation depends on the scale and distribution of the original variables. Standardization or centering prior to modeling may alter the variable structure and redistribute sources of variation, potentially affecting the interpretation of interaction effects. Therefore, products of metabolic and obesity-related indices (e.g., AIP-WHtR, AIP-BMI) are often constructed using raw variables to accurately reflect combined effects (18). Standardization may complicate the comparability of exposures across time points, particularly in time-dependent or cumulative exposure models. In contrast, composite indices based on the original scale may provide more consistent interpretation during follow-up (9). In addition, preserving original units enhances clinical interpretability and applicability. For example, composite indices integrating AIP with frailty status have demonstrated strong practical value in clinical risk assessment (28).

Previous studies on TyG, AIP, and obesity-related indices in relation to cardiovascular outcomes have primarily focused on their independent or combined effects, with limited systematic examination of interaction effects. In this study, we simultaneously evaluated multiple metabolic indices and their interactions with WHtR. The results indicated that only TyG, AIP, and CRI-II exhibited significant interactions. This finding highlights the importance of considering interaction effects, which have been less frequently examined in prior research, and provides additional support for the use of composite indices constructed on the original scale. In contrast, no interaction effect was observed for CHG. This discrepancy suggests heterogeneity among metabolic indices in capturing glucolipid metabolic disturbances and their coupling with fat distribution. As product terms are scale-dependent, standardization may influence the interpretation of interaction effects. Importantly, to verify the robustness of our findings, standardized analyses were performed across all models. The results remained consistent with the primary analysis, supporting the robustness of the findings.

Moreover, RCS revealed non-linear relationships between several cardiometabolic indices and CP risk. These indices reflect the degree of composite metabolic dysfunction, and as they increased from low to moderate levels, CP risk rose sharply, broadly consistent with known pathophysiological mechanisms of insulin resistance–related metabolic disturbances. For certain indices, such as CRI-II–WHtR, the risk curves plateaued or slightly declined at very high levels, indicating a potential saturation effect. Excluding participants with prevalent CP at baseline may have influenced the observed risk at extreme levels. Subgroup analyses indicated significant effect modification by age, dyslipidemia, and BMI, suggesting that the impact of combined metabolic abnormalities and central obesity may be more pronounced in certain high-risk subgroups. In contrast, the associations remained consistent across strata of smoking status, alcohol consumption, diabetes, and hypertension, supporting the robustness of the findings.

Abdominal obesity may promote the accumulation of CP (29). As a simple index of central obesity, WHtR has been widely used for cardiometabolic risk assessment (30), and our findings further support its role when integrated with metabolic indices. Asghari et al. (31) showed that WHtR has been reported to outperform BMI and the waist-to-hip ratio for predicting IMT. As these WHtR-based composite indices are readily obtainable. Therefore, these indices may provide additional epidemiological information for risk characterization of CP.

The development of CP involves a series of intricate processes, which are often associated with the accumulation of cholesterol and cholesteryl ester within the arterial intima. These lipids are usually derived from atherogenic apolipoprotein B (apoB)-containing lipoproteins, including low-density lipoprotein (LDL) and TG-rich lipoproteins (TRLs) (32). LDL is an CP-inducing particle encircled by a signature apoB component (33). HDL-C plays a key role in reverse cholesterol transport, which is the process by which excess cholesterol is removed from the bloodstream, thereby preventing the buildup of arterial plaques (34). From a pathophysiological perspective, apoB-containing lipoproteins, principally LDL, are a key source of cholesterol deposition in the arterial wall and foam-cell formation (35). These particles induce inflammatory responses and adhesion molecule expression in vascular endothelial cells, thereby promoting monocyte adhesion and migration (36). Furthermore, LDL amplifies local inflammation and processes related to plaque instability via immunoinflammatory signaling axes, such as the CD40/CD40L axis (37). Moreover, TRLs, such as very-low-density lipoproteins, TG, chylomicrons, and their remnants, can penetrate the arterial intima and deliver cholesterol, thereby triggering inflammation and promoting CP progression (38).

Based on the aforementioned pathophysiological mechanisms, the significant association of CRI-II–WHtR with incident CP observed in this study, along with its moderate predictive ability, may reflect the balance between atherogenic cholesterol burden and protective HDL-mediated reverse cholesterol transport. An elevated ratio may be associated with enhanced lipid deposition and foam cell formation within the arteries. WHtR, as an indicator of central obesity, is closely associated with visceral fat accumulation, insulin resistance, and chronic low-grade inflammation, which may contribute to endothelial dysfunction and promote the arterial retention of apoB-containing lipoproteins, which may be related to CP development (39–41). The multiplicative integration of these two measures may provide additional information on the combined effect between lipid abnormalities and central obesity than either index alone. Although exploratory subgroup analyses suggested that the association might be slightly stronger in individuals aged < 60 years, with normal BMI or dyslipidemia, these findings should be interpreted with caution, as they were not the primary focus of the study. Similarly, mediation analysis suggested that WHtR may partially mediate the relationship between cardiometabolic indices and CP; however, these findings are exploratory and should not be interpreted as causal evidence. Despite conventional discrimination metrics indicating limited improvement, CRI-II–WHtR consistently demonstrated relatively consistent performance. Its constituent measures can be obtained through routine physical examinations, representing a simple and readily obtainable composite measure. Overall, CRI-II–WHtR may provide additional epidemiological information for risk characterization of CP; however, its clinical relevance requires further validation in independent populations.

Some limitations of the study should be acknowledged. First, given the observational design, causality between cardiometabolic indices and CP was not established. Therefore, prospective studies and external validations are needed to confirm these associations. Second, although multiple potential confounders were adjusted for, residual confounding from unmeasured or inadequately controlled variables may still influence the observed associations. In addition, all reported estimates represent adjusted associations, and the inclusion of certain covariates may have introduced potential overadjustment. Secondary analyses and exploratory analyses may increase the risk of chance findings. Third, because the study population was limited to participants from China, may limit the generalizability of the findings. Accordingly, validation in large-scale, multicenter, multiethnic cohorts is warranted. Fourth, the mediation analysis warrants cautious interpretation. WHtR and cardiometabolic indices were measured within the same observational framework, and the temporal ordering among exposure, mediator, and outcome could not be clearly established, thereby limiting causal inference. Finally, the median follow-up duration was relatively short for a chronic process such as CP development. Although a sensitivity analysis excluding events within the first 2 years was performed, the presence of pre-existing subclinical plaques at baseline cannot be completely ruled out. Therefore, longer-term follow-up studies are needed to better capture the progression of CP.

In conclusion, this study suggests that multiple cardiometabolic indices are significantly associated with incident CP, with WHtR-based multiplicative composite indices showing larger effect estimates than additive models, indicating a potential combined effect between metabolic abnormalities and central obesity. These associations were consistent across time-dependent, time-weighted, and sex-stratified analyses. Moreover, a significant interaction between CRI-II and WHtR was observed, with a nonlinear relationship, further supporting the biological plausibility for their joint assessment. Regarding predictive performance, CRI-II–WHtR showed only modest improvement in model discrimination. Although differences across risk strata were observed, these findings should be interpreted cautiously. Overall, as a simple and readily obtainable composite index, CRI-II–WHtR provides additional epidemiological evidence for the association between cardiometabolic abnormalities and CP. However, external validation in independent populations is required before any potential clinical application.

## Data Availability

The raw data supporting the conclusions of this article are not publicly available to protect participant confidentiality and privacy. Requests to access the datasets should be directed to the corresponding author.
