# Supplementary material for: Waist-to-height ratio modifies the association between cardiometabolic indices and incident carotid plaque: evidence from a Chinese cohort
Source: Front Nutr. 2026 Jul 7;13:1853867. doi: 10.3389/fnut.2026.1853867 (PMC13385716; doi:10.3389/fnut.2026.1853867)
Supplement: Supplementary file 1 [file Data_Sheet_1.docx]

**Supplementary Materials**

**Supplementary Tables**

Table S1. The percentage of missing data in the present study

Table S2. Proportional hazards assumption test based on Schoenfeld residuals for Cox models incorporating restricted cubic splines for age

Table S3. Proportional hazards assumption test based on Schoenfeld residuals in Cox models with age as a continuous covariate

Table S4. Variance inflation factors of all predictors in each fully adjusted model

Table S5. Proportional hazards assumption test based on Schoenfeld residuals for time-dependent Cox models with product exposure variables

Table S6. Proportional hazards assumption test based on Schoenfeld residuals for Cox models with time-weighted average product exposure variables within a 2-year landmark framework

Table S7. Baseline characteristics of included and excluded participants

Table S8. Associations of additive cardiometabolic–WHtR composite indices with incident carotid plaque

Table S9. Associations between product-based cardiometabolic index–WHtR composites and incident carotid plaque in males

Table S10. Associations between product-based cardiometabolic index–WHtR composites and incident carotid plaque in females

Table S11. Associations of cardiometabolic indices with incident carotid plaque and their interactions with WHtR

Table S12. Associations of updated WHtR-based product indices with incident carotid plaque in time-dependent Cox models

Table S13. Associations of 2-year time-weighted average WHtR-based product indices with incident carotid plaque

Table S14. Time-dependent area under the curve of WHtR-based product indices for predicting carotid plaque

Table S15. Harrell’s C-index, Uno’s C-index, and integrated Brier score of WHtR-based product indices

Table S16. Associations of cardiometabolic index–WHtR product terms with incident carotid plaque after additional adjustment for clinical covariates

Table S17. Associations of cardiometabolic index–WHtR product terms with incident carotid plaque after excluding events within the first 2 years

Table S18. Associations of cardiometabolic index–WHtR product terms with incident carotid plaque in complete-case analysis

**Supplementary Figures**

Figure S1. Directed acyclic graph illustrating the assumed relationships between cardiometabolic indices and carotid plaque

Figure S2. Kaplan–Meier plots of cumulative incidence of carotid plaque: (A) RC; (B) RC/HDL-C; (C) Non-HDL-C; (D) CHG; (E) TyG; (F) AIP; (G) AC; (H) CRI-I; (I) CRI-II

Figure S3. Calibration curves of WHtR-based product indices for 3-year carotid plaque risk prediction

Figure S4. Decision curve analysis of WHtR-based product indices for 3-year carotid plaque risk prediction

Figure S5. Time-dependent Brier score curves of WHtR-based product indices

Figure S6. Exploratory analysis of WHtR in relation to cardiometabolic indices and incident carotid plaque

**Supplementary Methods**

The formulas for calculating cardiometabolic indices and their corresponding WHtR-derived composite indices are as follows:

WHtR = WC (cm) / Height (cm)

RC (mmol/L) = TC (mmol/L) − HDL-C (mmol/L) − LDL-C (mmol/L)

RC–WHtR = RC × WHtR

Non-HDL-C (mmol/L) = TC (mmol/L) − HDL-C (mmol/L)

Non-HDL-C–WHtR = Non-HDL-C × WHtR

RC/HDL-C = RC (mmol/L) / HDL-C (mmol/L)

RC/HDL-C–WHtR = RC/HDL-C × WHtR

CHG = ln [ (TC (mg/dL) × FPG (mg/dL)) / (2 × HDL-C (mg/dL)) ]

CHG–WHtR = CHG × WHtR

TyG = ln [ (TG (mg/dL) × FPG (mg/dL)) / 2 ]

TyG–WHtR = TyG × WHtR

AIP = log10 [ TG (mmol/L) / HDL-C (mmol/L) ]

AIP–WHtR = AIP × WHtR

AC = Non-HDL-C (mmol/L) / HDL-C (mmol/L)

AC–WHtR = AC × WHtR

CRI-I = TC (mmol/L) / HDL-C (mmol/L)

CRI-I–WHtR = CRI-I × WHtR

CRI-II = LDL-C (mmol/L) / HDL-C (mmol/L)

CRI-II–WHtR = CRI-II × WHtR

Note: All lipid parameters were measured in mmol/L and fasting plasma glucose (FPG) in mmol/L. For indices originally defined using mg/dL units (e.g., TyG and CHG), values were converted using the following standard conversion factors prior to calculation:

Cholesterol (TC, HDL-C, LDL-C): mg/dL = mmol/L × 38.67

Triglycerides (TG): mg/dL = mmol/L × 88.57

Glucose (FPG): mg/dL = mmol/L × 18

Abbreviations: AC, atherogenic coefficient; AIP, atherogenic index of plasma; CHG, cholesterol, high-density lipoprotein, and glucose index; CRI-I, Castelli’s index-I; CRI-II, Castelli’s index-II; FPG, fasting plasma glucose; HDL-C, high-density lipoprotein cholesterol; LDL-C, low-density lipoprotein cholesterol; Non-HDL-C, non-high-density lipoprotein cholesterol; RC, remnant cholesterol; RC/HDL-C, remnant cholesterol-to-high-density lipoprotein cholesterol ratio; TC, total cholesterol; TG, triglycerides; TyG, triglyceride-glucose index; WC, waist circumference; WHtR, waist-to-height ratio

Table S1. The percentage of missing data in the present study

| Variable | Total | Missing | Present | MissPct (%) |
| --- | --- | --- | --- | --- |
| AST | 6984 | 552 | 6432 | 7.90 |
| Albumin | 6984 | 284 | 6700 | 4.07 |
| Creatinine | 6984 | 38 | 6946 | 0.54 |
| Uricacid | 6984 | 12 | 6972 | 0.17 |
| ALT | 6984 | 6 | 6978 | 0.09 |
| SBP | 6984 | 4 | 6980 | 0.06 |
| DBP | 6984 | 4 | 6980 | 0.06 |
| RBC | 6984 | 4 | 6980 | 0.06 |
| WBC | 6984 | 4 | 6980 | 0.06 |
| Platelet count | 6984 | 4 | 6980 | 0.06 |
| Hemoglobin | 6984 | 4 | 6980 | 0.06 |
| Neutrophil | 6984 | 4 | 6980 | 0.06 |
| Lymphocyte | 6984 | 4 | 6980 | 0.06 |
| Monocyte count | 6984 | 4 | 6980 | 0.06 |
| Status | 6984 | 0 | 6984 | 0 |
| Time | 6984 | 0 | 6984 | 0 |
| Sex | 6984 | 0 | 6984 | 0 |
| Medication use | 6984 | 0 | 6984 | 0 |
| Diabetes | 6984 | 0 | 6984 | 0 |
| Dyslipidemia | 6984 | 0 | 6984 | 0 |
| Hypertension | 6984 | 0 | 6984 | 0 |
| Smoking status | 6984 | 0 | 6984 | 0 |
| Alcohol consumption | 6984 | 0 | 6984 | 0 |
| BMI | 6984 | 0 | 6984 | 0 |
| Height | 6984 | 0 | 6984 | 0 |
| Weight | 6984 | 0 | 6984 | 0 |
| WC | 6984 | 0 | 6984 | 0 |
| Age | 6984 | 0 | 6984 | 0 |
| FPG | 6984 | 0 | 6984 | 0 |
| HDL-C | 6984 | 0 | 6984 | 0 |
| TC | 6984 | 0 | 6984 | 0 |
| TG | 6984 | 0 | 6984 | 0 |
| LDL-C | 6984 | 0 | 6984 | 0 |

*Note:* This table summarizes the proportion of missing data for all variables included in the analysis. Variables with missing values were handled using multiple imputation by chained equations (MICE), as described in the Methods. Outcome variables and key exposure variables had no missing data.

Abbreviations: ALT, alanine aminotransferase; AST, aspartate aminotransferase; BMI, body mass index; DBP, diastolic blood pressure; FPG, fasting plasma glucose; HDL-C, high-density lipoprotein cholesterol; LDL-C, low-density lipoprotein cholesterol; RBC, red blood cell count; SBP, systolic blood pressure; TC, total cholesterol; TG, triglycerides; WBC, white blood cell count; WC, waist circumference

Table S2. Proportional hazards assumption test based on Schoenfeld residuals for Cox models incorporating restricted cubic splines for age

| Exposure | Analysis | Model | Term | Chisq | DF | *P* |
| --- | --- | --- | --- | --- | --- | --- |
| WHtR | Continuous | Model 3 | GLOBAL | 22.254 | 13 | 0.052 |
|  | Quartile | Model 3 | GLOBAL | 24.835 | 15 | 0.052 |
| RC | Continuous | Model 3 | GLOBAL | 21.775 | 13 | 0.059 |
|  | Quartile | Model 3 | GLOBAL | 24.107 | 15 | 0.063 |
| RC/HDL-C | Continuous | Model 3 | GLOBAL | 21.604 | 13 | 0.062 |
|  | Quartile | Model 3 | GLOBAL | 22.648 | 15 | 0.092 |
| Non-HDL-C | Continuous | Model 3 | GLOBAL | 22.377 | 13 | 0.050 |
|  | Quartile | Model 3 | GLOBAL | 23.705 | 15 | 0.070 |
| CHG | Continuous | Model 3 | GLOBAL | 22.905 | 13 | 0.043 |
|  | Quartile | Model 3 | GLOBAL | 23.885 | 15 | 0.067 |
| TyG | Continuous | Model 3 | GLOBAL | 21.158 | 13 | 0.070 |
|  | Quartile | Model 3 | GLOBAL | 21.244 | 15 | 0.129 |
| AIP | Continuous | Model 3 | GLOBAL | 21.456 | 13 | 0.064 |
|  | Quartile | Model 3 | GLOBAL | 22.098 | 15 | 0.105 |
| AC | Continuous | Model 3 | GLOBAL | 22.523 | 13 | 0.048 |
|  | Quartile | Model 3 | GLOBAL | 23.629 | 15 | 0.072 |
| CRI-I | Continuous | Model 3 | GLOBAL | 22.523 | 13 | 0.048 |
|  | Quartile | Model 3 | GLOBAL | 23.629 | 15 | 0.072 |
| CRI-II | Continuous | Model 3 | GLOBAL | 22.344 | 13 | 0.053 |
|  | Quartile | Model 3 | GLOBAL | 23.766 | 15 | 0.069 |

*Notes:* Note: Model 3 is adjusted for age (modeled using restricted cubic splines with 4 knots), sex, smoking status, alcohol consumption, diabetes, dyslipidemia, hypertension, platelet count, and monocyte count. All models were fitted using baseline exposure variables. The GLOBAL test refers to the Schoenfeld residual-based test for the proportional hazards assumption.

Abbreviations: AC, atherogenic coefficient; AIP, atherogenic index of plasma; CHG, cholesterol, high-density lipoprotein, and glucose index; Chisq, chi-square statistic; CRI-I, Castelli’s index-I; CRI-II, Castelli’s index-II; DF, degrees of freedom; HDL-C, high-density lipoprotein cholesterol; LDL-C, low-density lipoprotein cholesterol; Non-HDL-C, non-high-density lipoprotein cholesterol; RC, remnant cholesterol; RC/HDL-C, remnant cholesterol-to-high-density lipoprotein cholesterol ratio; TC, total cholesterol; TG, triglycerides; TyG, triglyceride-glucose index; WHtR, waist-to-height ratio

Table S3. Proportional hazards assumption test based on Schoenfeld residuals in Cox models with age as a continuous covariate

| Exposure | Analysis | Model | Term | Chisq | DF | *P* |
| --- | --- | --- | --- | --- | --- | --- |
| WHtR | Continuous | Model 3 | Expo_cont | 0.413 | 1 | 0.520 |
|  |  | Model 3 | Age | 10.300 | 1 | 0.001 |
|  |  | Model 3 | GLOBAL | 22.355 | 10 | 0.013 |
|  | Quartile | Model 3 | Expo_q | 2.103 | 3 | 0.551 |
|  |  | Model 3 | Age | 10.371 | 1 | 0.001 |
|  |  | Model 3 | GLOBAL | 24.884 | 12 | 0.015 |
| RC | Continuous | Model 3 | Expo_cont | 0.081 | 1 | 0.776 |
|  |  | Model 3 | Age | 10.564 | 1 | 0.001 |
|  |  | Model 3 | GLOBAL | 21.495 | 10 | 0.018 |
|  | Quartile | Model 3 | Expo_q | 6.287 | 3 | 0.098 |
|  |  | Model 3 | Age | 10.482 | 1 | 0.001 |
|  |  | Model 3 | GLOBAL | 24.662 | 12 | 0.017 |
| RC/HDL-C | Continuous | Model 3 | Expo_cont | 0.031 | 1 | 0.860 |
|  |  | Model 3 | Age | 10.488 | 1 | 0.001 |
|  |  | Model 3 | GLOBAL | 21.330 | 10 | 0.019 |
|  | Quartile | Model 3 | Expo_q | 4.713 | 3 | 0.194 |
|  |  | Model 3 | Age | 10.311 | 1 | 0.001 |
|  |  | Model 3 | GLOBAL | 22.995 | 12 | 0.028 |
| Non-HDL-C | Continuous | Model 3 | Expo_cont | 0.016 | 1 | 0.899 |
|  |  | Model 3 | Age | 12.333 | 1 | <0.001 |
|  |  | Model 3 | GLOBAL | 22.324 | 10 | 0.014 |
|  | Quartile | Model 3 | Expo_q | 0.717 | 3 | 0.869 |
|  |  | Model 3 | Age | 11.856 | 1 | <0.001 |
|  |  | Model 3 | GLOBAL | 23.093 | 12 | 0.027 |
| CHG | Continuous | Model 3 | Expo_cont | 0.180 | 1 | 0.672 |
|  |  | Model 3 | Age | 12.424 | 1 | <0.001 |
|  |  | Model 3 | GLOBAL | 23.469 | 10 | 0.009 |
|  | Quartile | Model 3 | Expo_q | 1.490 | 3 | 0.685 |
|  |  | Model 3 | Age | 11.910 | 1 | <0.001 |
|  |  | Model 3 | GLOBAL | 23.962 | 12 | 0.021 |
| TyG | Continuous | Model 3 | Expo_cont | 0.009 | 1 | 0.923 |
|  |  | Model 3 | Age | 10.391 | 1 | 0.001 |
|  |  | Model 3 | GLOBAL | 20.787 | 10 | 0.023 |
|  | Quartile | Model 3 | Expo_q | 0.655 | 3 | 0.884 |
|  |  | Model 3 | Age | 9.409 | 1 | 0.002 |
|  |  | Model 3 | GLOBAL | 20.158 | 12 | 0.064 |
| AIP | Continuous | Model 3 | Expo_cont | 0.004 | 1 | 0.950 |
|  |  | Model 3 | Age | 10.704 | 1 | 0.001 |
|  |  | Model 3 | GLOBAL | 21.261 | 10 | 0.019 |
|  | Quartile | Model 3 | Expo_q | 2.363 | 3 | 0.501 |
|  |  | Model 3 | Age | 10.715 | 1 | 0.001 |
|  |  | Model 3 | GLOBAL | 22.496 | 12 | 0.032 |
| AC | Continuous | Model 3 | Expo_cont | 0.583 | 1 | 0.445 |
|  |  | Model 3 | Age | 11.324 | 1 | <0.001 |
|  |  | Model 3 | GLOBAL | 22.650 | 10 | 0.012 |
|  | Quartile | Model 3 | Expo_q | 0.794 | 3 | 0.851 |
|  |  | Model 3 | Age | 13.171 | 1 | <0.001 |
|  |  | Model 3 | GLOBAL | 24.586 | 12 | 0.017 |
| CRI-I | Continuous | Model 3 | Expo_cont | 0.583 | 1 | 0.445 |
|  |  | Model 3 | Age | 11.324 | 1 | <0.001 |
|  |  | Model 3 | GLOBAL | 22.650 | 10 | 0.012 |
|  | Quartile | Model 3 | Expo_q | 0.794 | 3 | 0.851 |
|  |  | Model 3 | Age | 13.171 | 1 | <0.001 |
|  |  | Model 3 | GLOBAL | 24.586 | 12 | 0.017 |
| CRI-II | Continuous | Model 3 | Expo_cont | 0.477 | 1 | 0.490 |
|  |  | Model 3 | Age | 13.664 | 1 | <0.001 |
|  |  | Model 3 | GLOBAL | 26.248 | 10 | 0.003 |
|  | Quartile | Model 3 | Expo_q | 0.510 | 3 | 0.917 |
|  |  | Model 3 | Age | 14.619 | 1 | <0.001 |
|  |  | Model 3 | GLOBAL | 25.711 | 12 | 0.012 |

*Notes:* Model 3 is adjusted for age (entered as a continuous variable), sex, smoking status, alcohol consumption, diabetes, dyslipidemia, hypertension, platelet count, and monocyte count. This table presents proportional hazards assumption tests for models in which age was included as a linear term. The results indicate that age violated the proportional hazards assumption, supporting the use of restricted cubic splines in subsequent models. The GLOBAL test refers to the Schoenfeld residual-based test for the proportional hazards assumption.

Abbreviations: AC, atherogenic coefficient; AIP, atherogenic index of plasma; CHG, cholesterol, high-density lipoprotein, and glucose index; CRI-I, Castelli’s index-I; CRI-II, Castelli’s index-II; DF, degrees of freedom; exposure (continuous), continuous exposure variable; exposure (quartiles), categorical exposure variable by quartiles; GLOBAL, global test for proportional hazards assumption; Non-HDL-C, non-high-density lipoprotein cholesterol; RC, remnant cholesterol; RC/HDL-C, remnant cholesterol-to-high-density lipoprotein cholesterol ratio; TyG, triglyceride-glucose index; WHtR, waist-to-height ratio

Table S4. Variance inflation factors of all predictors in each fully adjusted model

| Outcome | Exposure model | Variable | VIF |
| --- | --- | --- | --- |
| CP | RC | RC | 1.363 |
| CP | RC | Age | 1.339 |
| CP | RC | Sex | 2.705 |
| CP | RC | Smoking status | 1.058 |
| CP | RC | Alcohol consumption | 1.057 |
| CP | RC | Hypertension | 1.358 |
| CP | RC | Diabetes | 1.214 |
| CP | RC | Dyslipidemia | 1.418 |
| CP | RC | Medication use | 1.510 |
| CP | RC | Platelet count | 1.191 |
| CP | RC | ALT | 1.236 |
| CP | RC | Monocyte count | 1.146 |
| CP | RC | Serum uric acid | 2.030 |
| CP | RC | Serum creatinine | 2.337 |
| CP | RC | Albumin | 1.281 |
| CP | RC | WHtR | 1.506 |
| CP | RC/HDL-C | RC/HDL-C | 1.132 |
| CP | RC/HDL-C | Age | 1.326 |
| CP | RC/HDL-C | Sex | 2.709 |
| CP | RC/HDL-C | Smoking status | 1.058 |
| CP | RC/HDL-C | Alcohol consumption | 1.057 |
| CP | RC/HDL-C | Hypertension | 1.357 |
| CP | RC/HDL-C | Diabetes | 1.211 |
| CP | RC/HDL-C | Dyslipidemia | 1.280 |
| CP | RC/HDL-C | Medication use | 1.510 |
| CP | RC/HDL-C | Platelet count | 1.191 |
| CP | RC/HDL-C | ALT | 1.232 |
| CP | RC/HDL-C | Monocyte count | 1.145 |
| CP | RC/HDL-C | Serum uric acid | 2.023 |
| CP | RC/HDL-C | Serum creatinine | 2.331 |
| CP | RC/HDL-C | Albumin | 1.262 |
| CP | RC/HDL-C | WHtR | 1.505 |
| CP | Non-HDL-C | Non-HDL-C | 1.406 |
| CP | Non-HDL-C | Age | 1.369 |
| CP | Non-HDL-C | Sex | 2.713 |
| CP | Non-HDL-C | Smoking status | 1.058 |
| CP | Non-HDL-C | Alcohol consumption | 1.057 |
| CP | Non-HDL-C | Hypertension | 1.357 |
| CP | Non-HDL-C | Diabetes | 1.210 |
| CP | Non-HDL-C | Dyslipidemia | 1.446 |
| CP | Non-HDL-C | Medication use | 1.512 |
| CP | Non-HDL-C | Platelet count | 1.200 |
| CP | Non-HDL-C | ALT | 1.237 |
| CP | Non-HDL-C | Monocyte count | 1.145 |
| CP | Non-HDL-C | Serum uric acid | 2.032 |
| CP | Non-HDL-C | Serum creatinine | 2.332 |
| CP | Non-HDL-C | Albumin | 1.271 |
| CP | Non-HDL-C | WHtR | 1.508 |
| CP | CHG | CHG | 2.132 |
| CP | CHG | Age | 1.335 |
| CP | CHG | Sex | 2.758 |
| CP | CHG | Smoking status | 1.058 |
| CP | CHG | Alcohol consumption | 1.057 |
| CP | CHG | Hypertension | 1.357 |
| CP | CHG | Diabetes | 1.348 |
| CP | CHG | Dyslipidemia | 1.537 |
| CP | CHG | Medication use | 1.510 |
| CP | CHG | Platelet count | 1.196 |
| CP | CHG | ALT | 1.238 |
| CP | CHG | Monocyte count | 1.146 |
| CP | CHG | Serum uric acid | 2.034 |
| CP | CHG | Serum creatinine | 2.333 |
| CP | CHG | Albumin | 1.264 |
| CP | CHG | WHtR | 1.576 |
| CP | TyG | TyG | 2.000 |
| CP | TyG | Age | 1.363 |
| CP | TyG | Sex | 2.713 |
| CP | TyG | Smoking status | 1.059 |
| CP | TyG | Alcohol consumption | 1.057 |
| CP | TyG | Hypertension | 1.358 |
| CP | TyG | Diabetes | 1.284 |
| CP | TyG | Dyslipidemia | 1.596 |
| CP | TyG | Medication use | 1.510 |
| CP | TyG | Platelet count | 1.196 |
| CP | TyG | ALT | 1.241 |
| CP | TyG | Monocyte count | 1.145 |
| CP | TyG | Serum uric acid | 2.061 |
| CP | TyG | Serum creatinine | 2.336 |
| CP | TyG | Albumin | 1.267 |
| CP | TyG | WHtR | 1.529 |
| CP | AIP | AIP | 2.037 |
| CP | AIP | Age | 1.328 |
| CP | AIP | Sex | 2.733 |
| CP | AIP | Smoking status | 1.059 |
| CP | AIP | Alcohol consumption | 1.057 |
| CP | AIP | Hypertension | 1.357 |
| CP | AIP | Diabetes | 1.215 |
| CP | AIP | Dyslipidemia | 1.667 |
| CP | AIP | Medication use | 1.510 |
| CP | AIP | Platelet count | 1.194 |
| CP | AIP | ALT | 1.237 |
| CP | AIP | Monocyte count | 1.146 |
| CP | AIP | Serum uric acid | 2.067 |
| CP | AIP | Serum creatinine | 2.332 |
| CP | AIP | Albumin | 1.259 |
| CP | AIP | WHtR | 1.557 |
| CP | AC | AC | 1.385 |
| CP | AC | Age | 1.326 |
| CP | AC | Sex | 2.715 |
| CP | AC | Smoking status | 1.058 |
| CP | AC | Alcohol consumption | 1.057 |
| CP | AC | Hypertension | 1.357 |
| CP | AC | Diabetes | 1.210 |
| CP | AC | Dyslipidemia | 1.397 |
| CP | AC | Medication use | 1.511 |
| CP | AC | Platelet count | 1.192 |
| CP | AC | ALT | 1.234 |
| CP | AC | Monocyte count | 1.146 |
| CP | AC | Serum uric acid | 2.028 |
| CP | AC | Serum creatinine | 2.331 |
| CP | AC | Albumin | 1.260 |
| CP | AC | WHtR | 1.520 |
| CP | CRI-I | CRI-I | 1.385 |
| CP | CRI-I | Age | 1.326 |
| CP | CRI-I | Sex | 2.715 |
| CP | CRI-I | Smoking status | 1.058 |
| CP | CRI-I | Alcohol consumption | 1.057 |
| CP | CRI-I | Hypertension | 1.357 |
| CP | CRI-I | Diabetes | 1.210 |
| CP | CRI-I | Dyslipidemia | 1.397 |
| CP | CRI-I | Medication use | 1.511 |
| CP | CRI-I | Platelet count | 1.192 |
| CP | CRI-I | ALT | 1.234 |
| CP | CRI-I | Monocyte count | 1.146 |
| CP | CRI-I | Serum uric acid | 2.028 |
| CP | CRI-I | Serum creatinine | 2.331 |
| CP | CRI-I | Albumin | 1.260 |
| CP | CRI-I | WHtR | 1.520 |
| CP | CRI-II | CRI-II | 1.540 |
| CP | CRI-II | Age | 1.326 |
| CP | CRI-II | Sex | 2.717 |
| CP | CRI-II | Smoking status | 1.058 |
| CP | CRI-II | Alcohol consumption | 1.058 |
| CP | CRI-II | Hypertension | 1.357 |
| CP | CRI-II | Diabetes | 1.210 |
| CP | CRI-II | Dyslipidemia | 1.443 |
| CP | CRI-II | Medication use | 1.511 |
| CP | CRI-II | Platelet count | 1.198 |
| CP | CRI-II | ALT | 1.234 |
| CP | CRI-II | Monocyte count | 1.147 |
| CP | CRI-II | Serum uric acid | 2.032 |
| CP | CRI-II | Serum creatinine | 2.335 |
| CP | CRI-II | Albumin | 1.258 |
| CP | CRI-II | WHtR | 1.542 |

*Note:* Variance inflation factors (VIFs) were calculated for all predictors included in each fully adjusted model. Each cardiometabolic index was entered into the model separately along with covariates to avoid structural collinearity arising from shared components among lipid-related variables. All VIF values were below the conventional threshold, indicating no evidence of substantial multicollinearity.

Abbreviations: AC, atherogenic coefficient; AIP, atherogenic index of plasma; ALT, alanine aminotransferase; CHG, cholesterol, high-density lipoprotein, and glucose index; CRI-I, Castelli’s index-I; CRI-II, Castelli’s index-II; Non-HDL-C, non-high-density lipoprotein cholesterol; RC, remnant cholesterol; RC/HDL-C, remnant cholesterol-to-high-density lipoprotein cholesterol ratio; TyG, triglyceride-glucose index; VIF, variance inflation factor; WHtR, waist-to-height ratio

Table S5. Proportional hazards assumption test based on Schoenfeld residuals for time-dependent Cox models with product exposure variables

| Exposure | Model | Analysis | Term | Chisq | DF | *P* |
| --- | --- | --- | --- | --- | --- | --- |
| RC–WHtR | Model3 | Continuous | GLOBAL | 11.997 | 12 | 0.446 |
| RC–WHtR | Model3 | Quartile | GLOBAL | 11.588 | 14 | 0.639 |
| RC-HDL-C–WHtR | Model3 | Continuous | GLOBAL | 11.587 | 12 | 0.479 |
| RC-HDL-C–WHtR | Model3 | Quartile | GLOBAL | 14.282 | 14 | 0.429 |
| Non-HDL-C–WHtR | Model3 | Continuous | GLOBAL | 13.846 | 12 | 0.311 |
| Non-HDL-C–WHtR | Model3 | Quartile | GLOBAL | 15.753 | 14 | 0.329 |
| CHG–WHtR | Model3 | Continuous | GLOBAL | 13.017 | 12 | 0.368 |
| CHG–WHtR | Model3 | Quartile | GLOBAL | 13.531 | 14 | 0.485 |
| TyG–WHtR | Model3 | Continuous | GLOBAL | 11.573 | 12 | 0.481 |
| TyG–WHtR | Model3 | Quartile | GLOBAL | 12.588 | 14 | 0.559 |
| AIP–WHtR | Model3 | Continuous | GLOBAL | 11.427 | 12 | 0.493 |
| AIP–WHtR | Model3 | Quartile | GLOBAL | 14.992 | 14 | 0.379 |
| AC–WHtR | Model3 | Continuous | GLOBAL | 11.946 | 12 | 0.450 |
| AC–WHtR | Model3 | Quartile | GLOBAL | 16.219 | 14 | 0.300 |
| CRI-I–WHtR | Model3 | Continuous | GLOBAL | 11.969 | 12 | 0.448 |
| CRI-I–WHtR | Model3 | Quartile | GLOBAL | 17.951 | 14 | 0.209 |
| CRI-II–WHtR | Model3 | Continuous | GLOBAL | 12.705 | 12 | 0.391 |
| CRI-II–WHtR | Model3 | Quartile | GLOBAL | 18.894 | 14 | 0.169 |

*Note:* Model 3 is adjusted for age (modeled using restricted cubic splines with 4 knots), sex, smoking status, alcohol consumption, diabetes, dyslipidemia, hypertension, platelet count, and monocyte count. All exposure variables were constructed as time-varying product terms of cardiometabolic indices and WHtR using a counting process (start–stop) approach, with follow-up time split at the second measurement. The GLOBAL test refers to the Schoenfeld residual-based test for the proportional hazards assumption.

Abbreviations: AC, atherogenic coefficient; AIP, atherogenic index of plasma; CHG, cholesterol, high-density lipoprotein, and glucose index; Chisq, chi-square statistic; CRI-I, Castelli’s index-I; CRI-II, Castelli’s index-II; DF, degrees of freedom; HDL-C, high-density lipoprotein cholesterol; LDL-C, low-density lipoprotein cholesterol; Non-HDL-C, non-high-density lipoprotein cholesterol; RC, remnant cholesterol; RC/HDL-C, remnant cholesterol-to-high-density lipoprotein cholesterol ratio; TC, total cholesterol; TG, triglycerides; TyG, triglyceride-glucose index; WHtR, waist-to-height ratio

Table S6. Proportional hazards assumption test based on Schoenfeld residuals for Cox models with time-weighted average product exposure variables within a 2-year landmark framework

| Exposure | Model | Analysis | Term | Chisq | DF | *P* |
| --- | --- | --- | --- | --- | --- | --- |
| TWAvgRC–WHtR | Model3 | Continuous | GLOBAL | 11.818 | 12 | 0.460 |
| TWAvgRC–WHtR | Model3 | Quartile | GLOBAL | 13.362 | 14 | 0.498 |
| TWAvgRC/HDL-C–WHtR | Model3 | Continuous | GLOBAL | 11.736 | 12 | 0.467 |
| TWAvgRC/HDL-C–WHtR | Model3 | Quartile | GLOBAL | 12.664 | 14 | 0.553 |
| TWAvgNon-HDL-C–WHtR | Model3 | Continuous | GLOBAL | 13.453 | 12 | 0.337 |
| TWAvgNon-HDL-C–WHtR | Model3 | Quartile | GLOBAL | 13.322 | 14 | 0.501 |
| TWAvgCHG–WHtR | Model3 | Continuous | GLOBAL | 13.054 | 12 | 0.365 |
| TWAvgCHG–WHtR | Model3 | Quartile | GLOBAL | 13.230 | 14 | 0.509 |
| TWAvgTyG–WHtR | Model3 | Continuous | GLOBAL | 12.161 | 12 | 0.433 |
| TWAvgTyG–WHtR | Model3 | Quartile | GLOBAL | 13.313 | 14 | 0.502 |
| TWAvgAIP–WHtR | Model3 | Continuous | GLOBAL | 11.821 | 12 | 0.460 |
| TWAvgAIP–WHtR | Model3 | Quartile | GLOBAL | 13.485 | 14 | 0.489 |
| TWAvgAC–WHtR | Model3 | Continuous | GLOBAL | 12.510 | 12 | 0.406 |
| TWAvgAC–WHtR | Model3 | Quartile | GLOBAL | 12.538 | 14 | 0.563 |
| TWAvgCRI-I–WHtR | Model3 | Continuous | GLOBAL | 12.568 | 12 | 0.401 |
| TWAvgCRI-I–WHtR | Model3 | Quartile | GLOBAL | 12.901 | 14 | 0.534 |
| TWAvgCRI-II–WHtR | Model3 | Continuous | GLOBAL | 14.071 | 12 | 0.296 |
| TWAvgCRI-II–WHtR | Model3 | Quartile | GLOBAL | 14.094 | 14 | 0.443 |

*Note:* Model 3 is adjusted for age (modeled using restricted cubic splines with 4 knots), sex, smoking status, alcohol consumption, diabetes, dyslipidemia, hypertension, platelet count, and monocyte count. All exposure variables were constructed as product terms of time-weighted average cardiometabolic indices and WHtR within a 2-year landmark framework. The GLOBAL test refers to the Schoenfeld residual-based test for the proportional hazards assumption.

Abbreviations: AC, atherogenic coefficient; AIP, atherogenic index of plasma; CHG, cholesterol, high-density lipoprotein, and glucose index; Chisq, chi-square statistic; CRI-I, Castelli’s index-I; CRI-II, Castelli’s index-II; DF, degrees of freedom; HDL-C, high-density lipoprotein cholesterol; LDL-C, low-density lipoprotein cholesterol; Non-HDL-C, non-high-density lipoprotein cholesterol; RC, remnant cholesterol; RC/HDL-C, remnant cholesterol-to-high-density lipoprotein cholesterol ratio; TC, total cholesterol; TG, triglycerides; TyG, triglyceride-glucose index; WHtR, waist-to-height ratio

Table S7. Baseline characteristics of included and excluded participants

| Variable | Overall (n=10360) | Excluded (n=3376) | Included (n=6984) | SMD |
| --- | --- | --- | --- | --- |
| Age, y, median (IQR) | 46.00 (38.00–52.00) | 46.00 (38.00–51.00) | 46.00 (37.00–52.00) | 0.016 |
| Follow-up time, y, median (IQR) | 2.36 (1.21–3.13) | 2.21 (1.07–2.75) | 2.47 (1.53–3.82) | 0.421 |
| Height, cm, median (IQR) | 171.00 (164.00–176.00) | 172.00 (168.00–177.00) | 170.00 (163.00–175.25) | 0.088 |
| Weight, kg, median (IQR) | 72.00 (62.00–81.00) | 75.00 (66.00–84.00) | 71.00 (61.00–80.00) | 0.315 |
| WC, cm, median (IQR) | 87.00 (79.00–94.00) | 90.00 (83.00–96.00) | 86.00 (78.00–93.00) | 0.399 |
| BMI, kg/m², median (IQR) | 24.61 (22.31–27.06) | 25.00 (22.59–27.62) | 24.46 (22.21–26.81) | 0.060 |
| SBP, mmHg, median (IQR) | 126.00 (115.00–136.00) | 128.00 (117.00–138.00) | 125.00 (115.00–135.00) | 0.152 |
| DBP, mmHg, median (IQR) | 78.00 (70.00–86.00) | 80.00 (73.00–88.00) | 77.00 (70.00–85.00) | 0.280 |
| RBC, ×10¹²/L, median (IQR) | 4.85 (4.51–5.19) | 4.79 (4.45–5.12) | 4.86 (4.52–5.20) | 0.151 |
| WBC, ×10⁹/L, median (IQR) | 5.79 (4.92–6.79) | 5.81 (4.88–6.99) | 5.78 (4.92–6.76) | 0.069 |
| Platelet count, ×10⁹/L, median (IQR) | 234.00 (202.00–269.00) | 238.00 (203.00–274.00) | 233.00 (202.00–269.00) | 0.082 |
| Hemoglobin, g/L, median (IQR) | 148.00 (135.00–158.00) | 145.00 (133.00–156.00) | 148.00 (136.00–158.00) | 0.131 |
| Neutrophil, ×10⁹/L, median (IQR) | 3.34 (2.70–4.12) | 3.31 (2.66–4.14) | 3.34 (2.71–4.11) | 0.027 |
| Lymphocyte, ×10⁹/L, median (IQR) | 1.91 (1.59–2.27) | 1.91 (1.59–2.31) | 1.91 (1.59–2.27) | 0.036 |
| Monocyte, ×10⁹/L, median (IQR) | 0.32 (0.24–0.40) | 0.35 (0.27–0.45) | 0.31 (0.24–0.39) | 0.348 |
| ALT, U/L, median (IQR) | 20.32 (14.57–30.24) | 19.70 (13.98–29.54) | 20.38 (14.64–30.34) | 0.008 |
| AST, U/L, median (IQR) | 20.25 (17.16–24.58) | 19.96 (16.97–24.71) | 20.31 (17.20–24.54) | 0.038 |
| Albumin, g/L, median (IQR) | 46.00 (44.40–47.65) | 45.51 (43.86–47.17) | 46.05 (44.50–47.71) | 0.244 |
| FBG, mmol/L, median (IQR) | 5.52 (5.23–5.90) | 5.47 (5.19–5.87) | 5.53 (5.24–5.91) | 0.034 |
| Serum uric acid, μmol/L, median (IQR) | 355.88 (289.92–423.12) | 354.25 (293.41–419.60) | 356.07 (289.24–423.93) | 0.037 |
| Serum creatinine, μmol/L, median (IQR) | 68.90 (57.84–80.00) | 66.80 (56.98–77.97) | 69.20 (57.95–80.21) | 0.110 |
| TC, mmol/L, median (IQR) | 4.95 (4.40–5.57) | 4.99 (4.42–5.61) | 4.93 (4.38–5.56) | 0.043 |
| TG, mmol/L, median (IQR) | 1.53 (1.10–2.14) | 1.68 (1.20–2.39) | 1.47 (1.06–2.05) | 0.219 |
| HDL-C, mmol/L, median (IQR) | 1.26 (1.07–1.50) | 1.21 (1.03–1.44) | 1.28 (1.09–1.52) | 0.208 |
| LDL-C, mmol/L, median (IQR) | 2.79 (2.31–3.30) | 2.93 (2.44–3.43) | 2.73 (2.26–3.24) | 0.209 |
| CP status, n (%) | 1845 (17.8) | 647 (19.2) | 1198 (17.2) | 0.052 |
| Sex, n (%) | 3673 (35.5) | 794 (23.5) | 2879 (41.2) | 0.385 |
| Medication use, n (%) | 771 (7.4) | 102 (3.0) | 669 (9.6) | 0.272 |
| Diabetes, n (%) | 505 (4.9) | 70 (2.1) | 435 (6.2) | 0.209 |
| Dyslipidemia, n (%) | 3709 (35.8) | 1356 (40.2) | 2353 (33.7) | 0.134 |
| Hypertension, n (%) | 2487 (24.0) | 866 (25.7) | 1621 (23.2) | 0.057 |
| Smoking status, n (%) | 1106 (10.7) | 641 (19.0) | 465 (6.7) | 0.375 |
| Alcohol consumption, n (%) | 1033 (10.0) | 564 (16.7) | 469 (6.7) | 0.315 |

Abbreviation: ALT, alanine aminotransferase; AST, aspartate aminotransferase; BMI, body mass index; DBP, diastolic blood pressure; FPG, fasting plasma glucose; HDL-C, high-density lipoprotein cholesterol; LDL-C, low-density lipoprotein cholesterol; RBC, red blood cell count; SBP, systolic blood pressure; SMD, standardized mean difference; TC, total cholesterol; TG, triglycerides; TyG, triglyceride-glucose index; WBC, white blood cell count; WC, waist circumference

Table S8. Associations of additive cardiometabolic–WHtR composite indices with incident carotid plaque

| Exposure | Levels | Events | PY | Incidence Rate_1000PY | Model 1 | | Model 2 | | Model 3 | |
| --- | --- | --- | --- | --- | --- | --- | --- | --- | --- | --- |
|  |  |  |  |  | HR (95%CI) | *P* | HR (95%CI) | *P* | HR (95%CI) | *P* |
| RC + WHtR | Continuous |  |  |  | 1.08 (1.04-1.12) | <0.001 | 1.08 (1.04-1.12) | <0.001 | 1.01 (0.97-1.06) | 0.668 |
|  | Per 1-SD |  |  |  | 1.13 (1.06-1.19) | <0.001 | 1.13 (1.06-1.19) | <0.001 | 1.02 (0.95-1.09) | 0.668 |
|  | Q1 | 134 | 4551.342 | 29.442 | Reference | — | Reference | — | Reference | — |
|  | Q2 | 268 | 4742.803 | 56.507 | 1.19 (0.96-1.47) | 0.114 | 1.19 (0.96-1.47) | 0.117 | 1.14 (0.92-1.41) | 0.224 |
|  | Q3 | 361 | 4895.292 | 73.744 | 1.29 (1.05-1.58) | 0.017 | 1.29 (1.05-1.58) | 0.017 | 1.14 (0.92-1.40) | 0.240 |
|  | Q4 | 435 | 5077.560 | 85.671 | 1.42 (1.16-1.74) | <0.001 | 1.42 (1.15-1.74) | <0.001 | 1.10 (0.88-1.37) | 0.409 |
|  | *P* for trend |  |  |  |  | <0.001 |  | <0.001 |  | 0.684 |
| RC/HDL-C + WHtR | Continuous |  |  |  | 1.06 (1.03-1.09) | <0.001 | 1.06 (1.03-1.09) | <0.001 | 1.02 (0.97-1.06) | 0.445 |
|  | Per 1-SD |  |  |  | 1.09 (1.05-1.14) | <0.001 | 1.09 (1.05-1.14) | <0.001 | 1.03 (0.96-1.10) | 0.445 |
|  | Q1 | 132 | 4652.537 | 28.372 | Reference | — | Reference | — | Reference | — |
|  | Q2 | 275 | 4825.888 | 56.984 | 1.31 (1.06-1.62) | 0.012 | 1.31 (1.06-1.62) | 0.012 | 1.26 (1.02-1.56) | 0.031 |
|  | Q3 | 363 | 4867.489 | 74.576 | 1.36 (1.10-1.68) | 0.004 | 1.36 (1.10-1.68) | 0.004 | 1.21 (0.97-1.49) | 0.086 |
|  | Q4 | 428 | 4921.083 | 86.973 | 1.57 (1.28-1.94) | <0.001 | 1.57 (1.28-1.94) | <0.001 | 1.24 (0.99-1.55) | 0.063 |
|  | *P* for trend |  |  |  |  | <0.001 |  | <0.001 |  | 0.232 |
| Non-HDL-C + WHtR | Continuous |  |  |  | 1.18 (1.13-1.22) | <0.001 | 1.18 (1.13-1.22) | <0.001 | 1.14 (1.09-1.19) | <0.001 |
|  | Per 1-SD |  |  |  | 1.29 (1.22-1.37) | <0.001 | 1.29 (1.22-1.37) | <0.001 | 1.22 (1.14-1.31) | <0.001 |
|  | Q1 | 142 | 4887.672 | 29.053 | Reference | — | Reference | — | Reference | — |
|  | Q2 | 260 | 4939.556 | 52.636 | 1.20 (0.98-1.48) | 0.080 | 1.20 (0.98-1.48) | 0.083 | 1.17 (0.95-1.45) | 0.131 |
|  | Q3 | 353 | 4831.780 | 73.058 | 1.46 (1.19-1.78) | <0.001 | 1.46 (1.19-1.78) | <0.001 | 1.36 (1.11-1.67) | 0.003 |
|  | Q4 | 443 | 4607.989 | 96.137 | 1.85 (1.52-2.25) | <0.001 | 1.85 (1.52-2.25) | <0.001 | 1.61 (1.30-1.99) | <0.001 |
|  | *P* for trend |  |  |  |  | <0.001 |  | <0.001 |  | <0.001 |
| CHG + WHtR | Continuous |  |  |  | 1.16 (1.11-1.20) | <0.001 | 1.16 (1.11-1.20) | <0.001 | 1.10 (1.05-1.15) | <0.001 |
|  | Per 1-SD |  |  |  | 1.28 (1.20-1.36) | <0.001 | 1.28 (1.20-1.37) | <0.001 | 1.18 (1.09-1.28) | <0.001 |
|  | Q1 | 131 | 4813.312 | 27.216 | Reference | — | Reference | — | Reference | — |
|  | Q2 | 264 | 4988.844 | 52.918 | 1.34 (1.08-1.66) | 0.007 | 1.34 (1.08-1.66) | 0.007 | 1.29 (1.04-1.60) | 0.021 |
|  | Q3 | 349 | 4894.241 | 71.308 | 1.49 (1.21-1.84) | <0.001 | 1.49 (1.21-1.85) | <0.001 | 1.38 (1.11-1.71) | 0.004 |
|  | Q4 | 454 | 4570.600 | 99.330 | 2.00 (1.62-2.46) | <0.001 | 2.00 (1.63-2.47) | <0.001 | 1.62 (1.28-2.05) | <0.001 |
|  | *P* for trend |  |  |  |  | <0.001 |  | <0.001 |  | <0.001 |
| TyG + WHtR | Continuous |  |  |  | 1.14 (1.09-1.18) | <0.001 | 1.14 (1.09-1.18) | <0.001 | 1.07 (1.02-1.12) | 0.005 |
|  | Per 1-SD |  |  |  | 1.24 (1.16-1.32) | <0.001 | 1.24 (1.16-1.32) | <0.001 | 1.12 (1.03-1.21) | 0.005 |
|  | Q1 | 122 | 4834.744 | 25.234 | Reference | — | Reference | — | Reference | — |
|  | Q2 | 267 | 4850.923 | 55.041 | 1.50 (1.20-1.86) | <0.001 | 1.50 (1.20-1.86) | <0.001 | 1.46 (1.17-1.82) | <0.001 |
|  | Q3 | 359 | 4876.041 | 73.625 | 1.63 (1.32-2.03) | <0.001 | 1.64 (1.32-2.03) | <0.001 | 1.51 (1.21-1.87) | <0.001 |
|  | Q4 | 450 | 4705.289 | 95.637 | 2.02 (1.64-2.50) | <0.001 | 2.03 (1.64-2.51) | <0.001 | 1.63 (1.28-2.06) | <0.001 |
|  | *P* for trend |  |  |  |  | <0.001 |  | <0.001 |  | <0.001 |
| AIP + WHtR | Continuous |  |  |  | 1.12 (1.08-1.17) | <0.001 | 1.12 (1.08-1.17) | <0.001 | 1.06 (1.01-1.11) | 0.018 |
|  | Per 1-SD |  |  |  | 1.22 (1.14-1.30) | <0.001 | 1.22 (1.14-1.30) | <0.001 | 1.10 (1.02-1.20) | 0.018 |
|  | Q1 | 125 | 4801.507 | 26.033 | Reference | — | Reference | — | Reference | — |
|  | Q2 | 304 | 4861.073 | 62.538 | 1.74 (1.41-2.15) | <0.001 | 1.74 (1.41-2.15) | <0.001 | 1.69 (1.37-2.09) | <0.001 |
|  | Q3 | 343 | 4865.534 | 70.496 | 1.57 (1.27-1.94) | <0.001 | 1.57 (1.27-1.94) | <0.001 | 1.41 (1.13-1.76) | 0.002 |
|  | Q4 | 426 | 4738.884 | 89.895 | 2.02 (1.63-2.49) | <0.001 | 2.02 (1.64-2.49) | <0.001 | 1.57 (1.24-1.99) | <0.001 |
|  | *P* for trend |  |  |  |  | <0.001 |  | <0.001 |  | 0.018 |
| AC + WHtR | Continuous |  |  |  | 1.09 (1.07-1.12) | <0.001 | 1.09 (1.07-1.12) | <0.001 | 1.06 (1.03-1.10) | <0.001 |
|  | Per 1-SD |  |  |  | 1.15 (1.11-1.20) | <0.001 | 1.15 (1.11-1.20) | <0.001 | 1.11 (1.05-1.17) | <0.001 |
|  | Q1 | 141 | 4812.486 | 29.299 | Reference | — | Reference | — | Reference | — |
|  | Q2 | 273 | 4904.738 | 55.660 | 1.30 (1.06-1.60) | 0.012 | 1.30 (1.06-1.60) | 0.013 | 1.25 (1.01-1.54) | 0.036 |
|  | Q3 | 358 | 4883.244 | 73.312 | 1.42 (1.16-1.75) | <0.001 | 1.43 (1.16-1.75) | <0.001 | 1.30 (1.05-1.60) | 0.015 |
|  | Q4 | 426 | 4666.529 | 91.288 | 1.75 (1.43-2.14) | <0.001 | 1.75 (1.43-2.14) | <0.001 | 1.41 (1.13-1.76) | 0.003 |
|  | *P* for trend |  |  |  |  | <0.001 |  | <0.001 |  | 0.005 |
| CRI-I + WHtR | Continuous |  |  |  | 1.09 (1.07-1.12) | <0.001 | 1.09 (1.07-1.12) | <0.001 | 1.06 (1.03-1.10) | <0.001 |
|  | Per 1-SD |  |  |  | 1.15 (1.11-1.20) | <0.001 | 1.15 (1.11-1.20) | <0.001 | 1.11 (1.05-1.17) | <0.001 |
|  | Q1 | 141 | 4812.486 | 29.299 | Reference | — | Reference | — | Reference | — |
|  | Q2 | 273 | 4904.738 | 55.660 | 1.30 (1.06-1.60) | 0.012 | 1.30 (1.06-1.60) | 0.013 | 1.25 (1.01-1.54) | 0.036 |
|  | Q3 | 358 | 4883.244 | 73.312 | 1.42 (1.16-1.75) | <0.001 | 1.43 (1.16-1.75) | <0.001 | 1.30 (1.05-1.60) | 0.015 |
|  | Q4 | 426 | 4666.529 | 91.288 | 1.75 (1.43-2.14) | <0.001 | 1.75 (1.43-2.14) | <0.001 | 1.41 (1.13-1.76) | 0.003 |
|  | *P* for trend |  |  |  |  | <0.001 |  | <0.001 |  | 0.005 |
| CRI-II + WHtR | Continuous |  |  |  | 1.16 (1.12-1.20) | <0.001 | 1.16 (1.12-1.20) | <0.001 | 1.13 (1.08-1.17) | <0.001 |
|  | Per 1-SD |  |  |  | 1.28 (1.21-1.36) | <0.001 | 1.29 (1.21-1.36) | <0.001 | 1.22 (1.14-1.30) | <0.001 |
|  | Q1 | 143 | 4916.766 | 29.084 | Reference | — | Reference | — | Reference | — |
|  | Q2 | 272 | 4965.502 | 54.778 | 1.37 (1.11-1.68) | 0.003 | 1.37 (1.11-1.68) | 0.003 | 1.29 (1.05-1.59) | 0.015 |
|  | Q3 | 356 | 4896.474 | 72.705 | 1.51 (1.23-1.85) | <0.001 | 1.51 (1.24-1.85) | <0.001 | 1.41 (1.15-1.74) | 0.001 |
|  | Q4 | 427 | 4488.255 | 95.137 | 2.02 (1.65-2.47) | <0.001 | 2.03 (1.66-2.48) | <0.001 | 1.72 (1.38-2.14) | <0.001 |
|  | *P* for trend |  |  |  |  | <0.001 |  | <0.001 |  | <0.001 |

*Notes:* PY, person-years; Incidence rates_1000PY are expressed per 1,000 PY. Age was modeled using restricted cubic splines with 4 knots. Model 1 is adjusted for age and sex; Model 2 is adjusted for age, sex, smoking status, and alcohol consumption; and Model 3 is adjusted for age, sex, smoking status, alcohol consumption, diabetes, dyslipidemia, hypertension, platelet count, and monocyte count. Additive composite indices are constructed by summing each cardiometabolic index and WHtR.

Abbreviations: AC, atherogenic coefficient; AIP, atherogenic index of plasma; CHG, cholesterol, high-density lipoprotein, and glucose index; CI, confidence interval; CRI-I, Castelli’s index-I; CRI-II, Castelli’s index-II; HR, hazard ratio; Non-HDL-C, non-high density lipoprotein cholesterol; RC, remnant cholesterol; RC/HDL-C, remnant cholesterol and high-density lipoprotein cholesterol ratio; TyG, triglyceride-glucose index; WHtR, waist-to-height ratio

Table S9. Associations between product-based cardiometabolic index–WHtR composites and incident carotid plaque in males

| Exposure | Levels | Events | PY | Incidence Rate_1000PY | Model 1 |  | Model 2 |  | Model 3 |  |
| --- | --- | --- | --- | --- | --- | --- | --- | --- | --- | --- |
|  |  |  |  |  | HR (95%CI) | *P* | HR (95%CI) | *P* | HR (95%CI) | *P* |
| RC–WHtR | Continuous |  |  |  | 1.01 (1.00-1.01) | 0.076 | 1.01 (1.00-1.01) | 0.080 | 1.00 (0.99-1.00) | 0.322 |
|  | Per 1-SD |  |  |  | 1.06 (0.99-1.13) | 0.076 | 1.06 (0.99-1.13) | 0.080 | 0.96 (0.89-1.04) | 0.322 |
|  | Q1 | 160 | 2533.287 | 63.159 | Reference | — | Reference | — | Reference | — |
|  | Q2 | 235 | 2818.314 | 83.383 | 1.10 (0.90-1.35) | 0.352 | 1.10 (0.90-1.35) | 0.355 | 1.03 (0.84-1.26) | 0.812 |
|  | Q3 | 233 | 2953.683 | 78.885 | 0.99 (0.81-1.22) | 0.956 | 0.99 (0.81-1.22) | 0.954 | 0.86 (0.70-1.07) | 0.171 |
|  | Q4 | 278 | 3023.295 | 91.953 | 1.23 (1.01-1.49) | 0.041 | 1.22 (1.01-1.49) | 0.043 | 0.95 (0.76-1.18) | 0.625 |
|  | *P* for trend |  |  |  |  | 0.086 |  | 0.088 |  | 0.324 |
| RC/HDL-C–WHtR | Continuous |  |  |  | 1.06 (0.96-1.17) | 0.265 | 1.06 (0.96-1.17) | 0.257 | 0.93 (0.78-1.10) | 0.378 |
|  | Per 1-SD |  |  |  | 1.04 (0.97-1.11) | 0.265 | 1.04 (0.97-1.11) | 0.257 | 0.95 (0.85-1.06) | 0.378 |
|  | Q1 | 175 | 2577.975 | 67.883 | Reference | — | Reference | — | Reference | — |
|  | Q2 | 232 | 2825.546 | 82.108 | 1.06 (0.87-1.29) | 0.578 | 1.06 (0.87-1.29) | 0.565 | 0.99 (0.81-1.21) | 0.917 |
|  | Q3 | 235 | 3009.718 | 78.080 | 1.04 (0.85-1.26) | 0.714 | 1.04 (0.86-1.27) | 0.685 | 0.88 (0.71-1.08) | 0.229 |
|  | Q4 | 264 | 2915.340 | 90.555 | 1.24 (1.02-1.50) | 0.028 | 1.24 (1.03-1.51) | 0.027 | 0.89 (0.70-1.13) | 0.340 |
|  | *P* for trend |  |  |  |  | 0.035 |  | 0.032 |  | 0.226 |
| Non-HDL-C–WHtR | Continuous |  |  |  | 1.54 (1.37-1.74) | <0.001 | 1.55 (1.37-1.74) | <0.001 | 1.43 (1.25-1.63) | <0.001 |
|  | Per 1-SD |  |  |  | 1.26 (1.18-1.34) | <0.001 | 1.26 (1.18-1.34) | <0.001 | 1.21 (1.12-1.30) | <0.001 |
|  | Q1 | 170 | 2971.611 | 57.208 | Reference | — | Reference | — | Reference | — |
|  | Q2 | 194 | 2821.661 | 68.754 | 1.12 (0.91-1.38) | 0.272 | 1.12 (0.91-1.38) | 0.278 | 1.10 (0.90-1.36) | 0.358 |
|  | Q3 | 241 | 2881.250 | 83.644 | 1.36 (1.11-1.65) | 0.002 | 1.36 (1.11-1.65) | 0.002 | 1.29 (1.05-1.58) | 0.013 |
|  | Q4 | 301 | 2654.057 | 113.411 | 1.84 (1.53-2.23) | <0.001 | 1.84 (1.53-2.23) | <0.001 | 1.68 (1.36-2.06) | <0.001 |
|  | *P* for trend |  |  |  |  | <0.001 |  | <0.001 |  | <0.001 |
| CHG–WHtR | Continuous |  |  |  | 1.66 (1.39-1.98) | <0.001 | 1.66 (1.39-1.99) | <0.001 | 1.31 (1.05-1.62) | 0.015 |
|  | Per 1-SD |  |  |  | 1.21 (1.13-1.29) | <0.001 | 1.21 (1.13-1.29) | <0.001 | 1.11 (1.02-1.20) | 0.015 |
|  | Q1 | 155 | 2909.471 | 53.274 | Reference | — | Reference | — | Reference | — |
|  | Q2 | 215 | 2907.177 | 73.955 | 1.23 (1.00-1.51) | 0.054 | 1.23 (1.00-1.52) | 0.049 | 1.18 (0.96-1.46) | 0.112 |
|  | Q3 | 255 | 2770.764 | 92.032 | 1.49 (1.22-1.82) | <0.001 | 1.49 (1.22-1.82) | <0.001 | 1.32 (1.07-1.63) | 0.009 |
|  | Q4 | 281 | 2741.167 | 102.511 | 1.63 (1.34-1.98) | <0.001 | 1.64 (1.34-1.99) | <0.001 | 1.29 (1.03-1.62) | 0.024 |
|  | *P* for trend |  |  |  |  | <0.001 |  | <0.001 |  | 0.019 |
| TyG–WHtR | Continuous |  |  |  | 1.31 (1.17-1.46) | <0.001 | 1.31 (1.17-1.46) | <0.001 | 1.10 (0.96-1.26) | 0.155 |
|  | Per 1-SD |  |  |  | 1.18 (1.10-1.26) | <0.001 | 1.18 (1.10-1.26) | <0.001 | 1.06 (0.98-1.15) | 0.155 |
|  | Q1 | 151 | 2853.734 | 52.913 | Reference | — | Reference | — | Reference | — |
|  | Q2 | 222 | 2891.216 | 76.784 | 1.17 (0.95-1.45) | 0.129 | 1.17 (0.95-1.44) | 0.132 | 1.10 (0.90-1.36) | 0.356 |
|  | Q3 | 261 | 2825.341 | 92.378 | 1.40 (1.15-1.72) | 0.001 | 1.40 (1.15-1.72) | <0.001 | 1.23 (0.99-1.51) | 0.056 |
|  | Q4 | 272 | 2758.287 | 98.612 | 1.51 (1.24-1.85) | <0.001 | 1.51 (1.24-1.85) | <0.001 | 1.15 (0.91-1.44) | 0.239 |
|  | *P* for trend |  |  |  |  | <0.001 |  | <0.001 |  | 0.184 |
| AIP–WHtR | Continuous |  |  |  | 2.30 (1.51-3.50) | <0.001 | 2.31 (1.52-3.52) | <0.001 | 1.24 (0.70-2.20) | 0.462 |
|  | Per 1-SD |  |  |  | 1.14 (1.07-1.21) | <0.001 | 1.14 (1.07-1.21) | <0.001 | 1.03 (0.95-1.13) | 0.462 |
|  | Q1 | 185 | 2904.584 | 63.692 | Reference | — | Reference | — | Reference | — |
|  | Q2 | 235 | 2840.437 | 82.734 | 1.30 (1.07-1.57) | 0.009 | 1.30 (1.07-1.58) | 0.007 | 1.25 (1.03-1.52) | 0.025 |
|  | Q3 | 239 | 2828.578 | 84.495 | 1.32 (1.09-1.60) | 0.004 | 1.33 (1.10-1.61) | 0.004 | 1.18 (0.96-1.45) | 0.117 |
|  | Q4 | 247 | 2754.981 | 89.656 | 1.46 (1.21-1.77) | <0.001 | 1.47 (1.21-1.78) | <0.001 | 1.08 (0.84-1.40) | 0.536 |
|  | *P* for trend |  |  |  |  | <0.001 |  | <0.001 |  | 0.455 |
| AC–WHtR | Continuous |  |  |  | 1.10 (1.06-1.14) | <0.001 | 1.10 (1.06-1.15) | <0.001 | 1.07 (1.01-1.13) | 0.015 |
|  | Per 1-SD |  |  |  | 1.10 (1.06-1.14) | <0.001 | 1.10 (1.06-1.14) | <0.001 | 1.07 (1.01-1.13) | 0.015 |
|  | Q1 | 183 | 2935.802 | 62.334 | Reference | — | Reference | — | Reference | — |
|  | Q2 | 221 | 2835.925 | 77.929 | 1.21 (1.00-1.48) | 0.054 | 1.22 (1.00-1.48) | 0.050 | 1.21 (0.99-1.47) | 0.064 |
|  | Q3 | 226 | 2906.538 | 77.756 | 1.31 (1.07-1.59) | 0.008 | 1.31 (1.08-1.59) | 0.007 | 1.25 (1.01-1.53) | 0.038 |
|  | Q4 | 276 | 2650.314 | 104.139 | 1.71 (1.42-2.07) | <0.001 | 1.72 (1.43-2.08) | <0.001 | 1.53 (1.22-1.93) | <0.001 |
|  | *P* for trend |  |  |  |  | <0.001 |  | <0.001 |  | <0.001 |
| CRI-I–WHtR | Continuous |  |  |  | 1.10 (1.06-1.14) | <0.001 | 1.10 (1.06-1.15) | <0.001 | 1.07 (1.01-1.13) | 0.014 |
|  | Per 1-SD |  |  |  | 1.10 (1.06-1.15) | <0.001 | 1.10 (1.06-1.15) | <0.001 | 1.07 (1.01-1.13) | 0.014 |
|  | Q1 | 177 | 2928.876 | 60.433 | Reference | — | Reference | — | Reference | — |
|  | Q2 | 223 | 2843.318 | 78.429 | 1.21 (0.99-1.48) | 0.057 | 1.22 (1.00-1.48) | 0.053 | 1.20 (0.98-1.47) | 0.071 |
|  | Q3 | 232 | 2904.647 | 79.872 | 1.35 (1.11-1.64) | 0.003 | 1.35 (1.11-1.65) | 0.002 | 1.28 (1.04-1.57) | 0.021 |
|  | Q4 | 274 | 2651.738 | 103.328 | 1.71 (1.41-2.06) | <0.001 | 1.71 (1.42-2.07) | <0.001 | 1.51 (1.20-1.91) | <0.001 |
|  | *P* for trend |  |  |  |  | <0.001 |  | <0.001 |  | <0.001 |
| CRI-II–WHtR | Continuous |  |  |  | 1.47 (1.33-1.63) | <0.001 | 1.48 (1.34-1.64) | <0.001 | 1.39 (1.23-1.58) | <0.001 |
|  | Per 1-SD |  |  |  | 1.22 (1.15-1.28) | <0.001 | 1.22 (1.16-1.28) | <0.001 | 1.18 (1.11-1.26) | <0.001 |
|  | Q1 | 184 | 3010.543 | 61.119 | Reference | — | Reference | — | Reference | — |
|  | Q2 | 224 | 2943.115 | 76.110 | 1.24 (1.02-1.51) | 0.028 | 1.25 (1.03-1.52) | 0.026 | 1.25 (1.03-1.52) | 0.027 |
|  | Q3 | 230 | 2840.207 | 80.980 | 1.42 (1.17-1.72) | <0.001 | 1.43 (1.17-1.73) | <0.001 | 1.38 (1.13-1.69) | 0.001 |
|  | Q4 | 268 | 2534.714 | 105.732 | 1.80 (1.49-2.17) | <0.001 | 1.81 (1.50-2.19) | <0.001 | 1.65 (1.34-2.05) | <0.001 |
|  | *P* for trend |  |  |  |  | <0.001 |  | <0.001 |  | <0.001 |

*Notes:* PY, person-years; Incidence rates_1000PY are expressed per 1,000 PY. Age was modeled using restricted cubic splines with 4 knots. Model 1 is adjusted for age and sex; Model 2 is adjusted for age, sex, smoking status, and alcohol consumption; and Model 3 is adjusted for age, sex, smoking status, alcohol consumption, diabetes, dyslipidemia, hypertension, platelet count, and monocyte count. Multiplicative composite indices are constructed by multiplying each cardiometabolic index and WHtR.

Abbreviation: AC, atherogenic coefficient; AIP, atherogenic index of plasma; CHG, cholesterol, high-density lipoprotein, and glucose index; CI, confidence interval; CRI-I, Castelli’s index-I; CRI-II, Castelli’s index-II; HR, hazard ratio; Non-HDL-C, non-high density lipoprotein cholesterol; RC, remnant cholesterol; RC/HDL-C, remnant cholesterol and high density lipoprotein cholesterol ratio; TyG, triglyceride-glucose index; WHtR, waist-to-height ratio

Table S10. Associations between product-based cardiometabolic index–WHtR composites and incident carotid plaque in females

| Exposure | Levels | Events | PY | Incidence Rate_1000PY | Model 1 | | Model 2 | | Model 3 | |
| --- | --- | --- | --- | --- | --- | --- | --- | --- | --- | --- |
|  |  |  |  |  | HR (95%CI) | *P* | HR (95%CI) | *P* | HR (95%CI) | *P* |
| RC–WHtR | Continuous |  |  |  | 1.01 (1.00-1.02) | 0.082 | 1.01 (1.00-1.03) | 0.078 | 1.00 (0.99-1.02) | 0.686 |
|  | Per 1-SD |  |  |  | 1.09 (0.99-1.20) | 0.082 | 1.09 (0.99-1.20) | 0.078 | 1.03 (0.91-1.16) | 0.686 |
|  | Q1 | 41 | 1846.344 | 22.206 | Reference | — | Reference | — | Reference | — |
|  | Q2 | 50 | 1860.769 | 26.871 | 1.00 (0.66-1.52) | 0.982 | 1.00 (0.66-1.52) | 0.985 | 1.00 (0.66-1.52) | 0.995 |
|  | Q3 | 83 | 2046.001 | 40.567 | 1.21 (0.82-1.77) | 0.332 | 1.20 (0.82-1.75) | 0.361 | 1.16 (0.79-1.71) | 0.447 |
|  | Q4 | 118 | 2185.305 | 53.997 | 1.17 (0.80-1.71) | 0.407 | 1.18 (0.81-1.72) | 0.395 | 0.98 (0.65-1.48) | 0.927 |
|  | *P* for trend |  |  |  |  | 0.291 |  | 0.282 |  | 0.964 |
| RC/HDL-C–WHtR | Continuous |  |  |  | 1.67 (1.11-2.49) | 0.013 | 1.67 (1.12-2.49) | 0.013 | 1.33 (0.80-2.21) | 0.274 |
|  | Per 1-SD |  |  |  | 1.12 (1.02-1.22) | 0.013 | 1.12 (1.02-1.22) | 0.013 | 1.06 (0.95-1.18) | 0.274 |
|  | Q1 | 37 | 1808.005 | 20.465 | Reference | — | Reference | — | Reference | — |
|  | Q2 | 62 | 1996.648 | 31.052 | 1.19 (0.79-1.79) | 0.414 | 1.17 (0.77-1.76) | 0.460 | 1.20 (0.79-1.81) | 0.394 |
|  | Q3 | 81 | 2060.851 | 39.304 | 1.43 (0.96-2.12) | 0.075 | 1.42 (0.96-2.11) | 0.080 | 1.40 (0.94-2.08) | 0.096 |
|  | Q4 | 112 | 2072.914 | 54.030 | 1.52 (1.03-2.23) | 0.034 | 1.51 (1.03-2.22) | 0.035 | 1.30 (0.86-1.99) | 0.215 |
|  | *P* for trend |  |  |  |  | 0.019 |  | 0.017 |  | 0.184 |
| Non-HDL-C–WHtR | Continuous |  |  |  | 1.53 (1.27-1.85) | <0.001 | 1.55 (1.28-1.87) | <0.001 | 1.50 (1.19-1.89) | <0.001 |
|  | Per 1-SD |  |  |  | 1.26 (1.14-1.40) | <0.001 | 1.27 (1.15-1.41) | <0.001 | 1.25 (1.10-1.42) | <0.001 |
|  | Q1 | 34 | 2030.625 | 16.744 | Reference | — | Reference | — | Reference | — |
|  | Q2 | 55 | 1993.215 | 27.594 | 1.27 (0.83-1.97) | 0.274 | 1.27 (0.82-1.96) | 0.277 | 1.27 (0.82-1.96) | 0.281 |
|  | Q3 | 80 | 2021.441 | 39.576 | 1.57 (1.04-2.37) | 0.031 | 1.57 (1.04-2.37) | 0.033 | 1.57 (1.04-2.38) | 0.033 |
|  | Q4 | 123 | 1893.138 | 64.972 | 1.97 (1.32-2.96) | <0.001 | 1.99 (1.33-2.99) | <0.001 | 1.93 (1.25-2.99) | 0.003 |
|  | *P* for trend |  |  |  |  | <0.001 |  | <0.001 |  | 0.002 |
| CHG–WHtR | Continuous |  |  |  | 1.76 (1.37-2.27) | <0.001 | 1.77 (1.37-2.28) | <0.001 | 1.51 (1.12-2.04) | 0.008 |
|  | Per 1-SD |  |  |  | 1.25 (1.13-1.38) | <0.001 | 1.25 (1.13-1.38) | <0.001 | 1.18 (1.04-1.32) | 0.008 |
|  | Q1 | 36 | 1977.885 | 18.201 | Reference | — | Reference | — | Reference | — |
|  | Q2 | 55 | 1993.989 | 27.583 | 1.32 (0.86-2.03) | 0.198 | 1.31 (0.85-2.00) | 0.220 | 1.34 (0.87-2.05) | 0.181 |
|  | Q3 | 82 | 2012.046 | 40.755 | 1.58 (1.05-2.36) | 0.027 | 1.57 (1.05-2.35) | 0.029 | 1.53 (1.02-2.30) | 0.042 |
|  | Q4 | 119 | 1954.499 | 60.885 | 1.94 (1.30-2.88) | 0.001 | 1.92 (1.29-2.85) | 0.001 | 1.67 (1.10-2.54) | 0.017 |
|  | *P* for trend |  |  |  |  | <0.001 |  | <0.001 |  | 0.017 |
| TyG–WHtR | Continuous |  |  |  | 1.37 (1.18-1.60) | <0.001 | 1.37 (1.18-1.60) | <0.001 | 1.24 (1.03-1.49) | 0.025 |
|  | Per 1-SD |  |  |  | 1.23 (1.11-1.35) | <0.001 | 1.22 (1.11-1.35) | <0.001 | 1.15 (1.02-1.29) | 0.025 |
|  | Q1 | 39 | 1990.892 | 19.589 | Reference | — | Reference | — | Reference | — |
|  | Q2 | 46 | 1978.999 | 23.244 | 1.03 (0.67-1.59) | 0.885 | 1.03 (0.67-1.59) | 0.899 | 1.06 (0.68-1.63) | 0.803 |
|  | Q3 | 83 | 2008.805 | 41.318 | 1.46 (0.98-2.16) | 0.060 | 1.45 (0.98-2.15) | 0.066 | 1.42 (0.95-2.11) | 0.083 |
|  | Q4 | 124 | 1959.722 | 63.274 | 1.85 (1.26-2.70) | 0.002 | 1.83 (1.25-2.68) | 0.002 | 1.64 (1.09-2.47) | 0.017 |
|  | *P* for trend |  |  |  |  | <0.001 |  | <0.001 |  | 0.005 |
| AIP–WHtR | Continuous |  |  |  | 4.36 (1.83-10.39) | <0.001 | 4.30 (1.80-10.24) | <0.001 | 2.88 (1.00-8.27) | 0.050 |
|  | Per 1-SD |  |  |  | 1.20 (1.08-1.34) | <0.001 | 1.20 (1.08-1.34) | <0.001 | 1.14 (1.00-1.30) | 0.050 |
|  | Q1 | 46 | 2061.595 | 22.313 | Reference | — | Reference | — | Reference | — |
|  | Q2 | 59 | 1959.036 | 30.117 | 1.22 (0.83-1.81) | 0.305 | 1.23 (0.83-1.81) | 0.303 | 1.22 (0.83-1.80) | 0.318 |
|  | Q3 | 91 | 1976.406 | 46.043 | 1.68 (1.17-2.40) | 0.005 | 1.67 (1.17-2.39) | 0.005 | 1.66 (1.16-2.39) | 0.006 |
|  | Q4 | 96 | 1941.381 | 49.449 | 1.57 (1.09-2.24) | 0.014 | 1.56 (1.09-2.24) | 0.014 | 1.27 (0.85-1.90) | 0.239 |
|  | *P* for trend |  |  |  |  | 0.005 |  | 0.005 |  | 0.095 |
| AC–WHtR | Continuous |  |  |  | 1.55 (1.30-1.85) | <0.001 | 1.56 (1.31-1.86) | <0.001 | 1.50 (1.21-1.85) | <0.001 |
|  | Per 1-SD |  |  |  | 1.28 (1.16-1.41) | <0.001 | 1.28 (1.16-1.41) | <0.001 | 1.25 (1.11-1.41) | <0.001 |
|  | Q1 | 41 | 1976.925 | 20.739 | Reference | — | Reference | — | Reference | — |
|  | Q2 | 62 | 2088.747 | 29.683 | 1.11 (0.75-1.66) | 0.602 | 1.11 (0.74-1.65) | 0.623 | 1.10 (0.74-1.64) | 0.634 |
|  | Q3 | 76 | 1984.161 | 38.303 | 1.39 (0.95-2.04) | 0.093 | 1.39 (0.95-2.05) | 0.091 | 1.39 (0.94-2.06) | 0.096 |
|  | Q4 | 113 | 1888.585 | 59.833 | 1.78 (1.23-2.58) | 0.002 | 1.80 (1.24-2.60) | 0.002 | 1.68 (1.11-2.54) | 0.013 |
|  | *P* for trend |  |  |  |  | <0.001 |  | <0.001 |  | 0.005 |
| CRI-I–WHtR | Continuous |  |  |  | 1.52 (1.29-1.80) | <0.001 | 1.53 (1.29-1.81) | <0.001 | 1.46 (1.19-1.79) | <0.001 |
|  | Per 1-SD |  |  |  | 1.28 (1.16-1.42) | <0.001 | 1.29 (1.16-1.42) | <0.001 | 1.25 (1.11-1.41) | <0.001 |
|  | Q1 | 40 | 1961.951 | 20.388 | Reference | — | Reference | — | Reference | — |
|  | Q2 | 60 | 2087.152 | 28.747 | 1.05 (0.70-1.58) | 0.804 | 1.04 (0.70-1.57) | 0.835 | 1.02 (0.68-1.52) | 0.942 |
|  | Q3 | 79 | 1982.831 | 39.842 | 1.41 (0.96-2.08) | 0.080 | 1.40 (0.95-2.06) | 0.088 | 1.37 (0.92-2.02) | 0.119 |
|  | Q4 | 113 | 1906.485 | 59.271 | 1.74 (1.20-2.53) | 0.004 | 1.75 (1.20-2.54) | 0.003 | 1.59 (1.05-2.42) | 0.028 |
|  | *P* for trend |  |  |  |  | <0.001 |  | <0.001 |  | 0.007 |
| CRI-II–WHtR | Continuous |  |  |  | 2.00 (1.55-2.58) | <0.001 | 2.02 (1.56-2.60) | <0.001 | 1.92 (1.43-2.59) | <0.001 |
|  | Per 1-SD |  |  |  | 1.33 (1.20-1.47) | <0.001 | 1.33 (1.20-1.48) | <0.001 | 1.31 (1.16-1.48) | <0.001 |
|  | Q1 | 46 | 2044.607 | 22.498 | Reference | — | Reference | — | Reference | — |
|  | Q2 | 59 | 2094.516 | 28.169 | 0.99 (0.67-1.46) | 0.948 | 0.98 (0.66-1.45) | 0.922 | 0.99 (0.67-1.46) | 0.965 |
|  | Q3 | 79 | 1979.171 | 39.916 | 1.30 (0.90-1.89) | 0.159 | 1.31 (0.90-1.89) | 0.156 | 1.30 (0.89-1.89) | 0.175 |
|  | Q4 | 108 | 1820.124 | 59.337 | 1.77 (1.24-2.53) | 0.002 | 1.79 (1.25-2.55) | 0.001 | 1.70 (1.16-2.50) | 0.007 |
|  | *P* for trend |  |  |  |  | <0.001 |  | <0.001 |  | 0.002 |

*Notes:* PY, person-years; Incidence rates_1000PY are expressed per 1,000 PY. Age was modeled using restricted cubic splines with 4 knots. Model 1 is adjusted for age and sex; Model 2 is adjusted for age, sex, smoking status, and alcohol consumption; and Model 3 is adjusted for age, sex, smoking status, alcohol consumption, diabetes, dyslipidemia, hypertension, platelet count, and monocyte count. Multiplicative composite indices are constructed by multiplying each cardiometabolic index and WHtR.

Abbreviation: AC, atherogenic coefficient; AIP, atherogenic index of plasma; CHG, cholesterol, high-density lipoprotein, and glucose index; CI, confidence interval; CRI-I, Castelli’s index-I; CRI-II, Castelli’s index-II; HR, hazard ratio; Non-HDL-C, non-high density lipoprotein cholesterol; RC, remnant cholesterol; RC/HDL-C, remnant cholesterol and high density lipoprotein cholesterol ratio; TyG, triglyceride-glucose index; WHtR, waist-to-height ratio

Table S11. Associations of cardiometabolic indices with incident carotid plaque and their interactions with WHtR

| Exposure | Levels | Model 1 | | Model 2 | | Model 3 | |
| --- | --- | --- | --- | --- | --- | --- | --- |
|  |  | HR (95 CI%) | *P* | HR (95 CI%) | *P* | HR (95 CI%) | *P* |
| RC | Continuous | 1.00 (1.00-1.01) | 0.112 | 1.00 (1.00-1.01) | 0.114 | 1.00 (1.00-1.00) | 0.762 |
|  | Per 1-SD | 1.05 (0.99-1.12) | 0.112 | 1.05 (0.99-1.12) | 0.114 | 0.99 (0.92-1.07) | 0.762 |
|  | P for interaction |  | 0.898 |  | 0.899 |  | 0.583 |
| RC/HDL-C | Continuous | 1.03 (0.98-1.08) | 0.236 | 1.03 (0.98-1.08) | 0.235 | 1.00 (0.92-1.08) | 0.951 |
|  | Per 1-SD | 1.03 (0.98-1.09) | 0.236 | 1.03 (0.98-1.09) | 0.235 | 1.00 (0.92-1.08) | 0.951 |
|  | P for interaction |  | 0.764 |  | 0.760 |  | 0.301 |
| Non-HDL-C | Continuous | 1.28 (1.20-1.36) | <0.001 | 1.28 (1.20-1.36) | <0.001 | 1.27 (1.18-1.36) | <0.001 |
|  | Per 1-SD | 1.25 (1.18-1.32) | <0.001 | 1.25 (1.18-1.32) | <0.001 | 1.24 (1.16-1.32) | <0.001 |
|  | P for interaction |  | 0.243 |  | 0.249 |  | 0.151 |
| CHG | Continuous | 1.84 (1.54-2.19) | <0.001 | 1.84 (1.54-2.20) | <0.001 | 1.67 (1.35-2.07) | <0.001 |
|  | Per 1-SD | 1.24 (1.17-1.32) | <0.001 | 1.24 (1.17-1.33) | <0.001 | 1.20 (1.11-1.30) | <0.001 |
|  | P for interaction |  | 0.388 |  | 0.398 |  | 0.239 |
| TyG | Continuous | 1.37 (1.22-1.53) | <0.001 | 1.37 (1.22-1.53) | <0.001 | 1.22 (1.06-1.40) | 0.005 |
|  | Per 1-SD | 1.20 (1.12-1.28) | <0.001 | 1.20 (1.12-1.28) | <0.001 | 1.12 (1.04-1.22) | 0.005 |
|  | P for interaction |  | 0.061 |  | 0.054 |  | 0.016 |
| AIP | Continuous | 1.69 (1.35-2.12) | <0.001 | 1.69 (1.35-2.12) | <0.001 | 1.35 (1.03-1.79) | 0.033 |
|  | Per 1-SD | 1.17 (1.09-1.25) | <0.001 | 1.17 (1.09-1.25) | <0.001 | 1.09 (1.01-1.19) | 0.033 |
|  | P for interaction |  | 0.058 |  | 0.062 |  | 0.020 |
| AC | Continuous | 1.05 (1.03-1.07) | <0.001 | 1.05 (1.03-1.07) | <0.001 | 1.04 (1.02-1.07) | <0.001 |
|  | Per 1-SD | 1.08 (1.05-1.12) | <0.001 | 1.08 (1.05-1.12) | <0.001 | 1.07 (1.03-1.11) | <0.001 |
|  | P for interaction |  | 0.548 |  | 0.572 |  | 0.673 |
| CRI-I | Continuous | 1.05 (1.03-1.07) | <0.001 | 1.05 (1.03-1.07) | <0.001 | 1.04 (1.02-1.07) | <0.001 |
|  | Per 1-SD | 1.08 (1.05-1.12) | <0.001 | 1.08 (1.05-1.12) | <0.001 | 1.07 (1.03-1.11) | <0.001 |
|  | P for interaction |  | 0.167 |  | 0.109 |  | 0.083 |
| CRI-II | Continuous | 1.24 (1.18-1.30) | <0.001 | 1.24 (1.18-1.30) | <0.001 | 1.23 (1.16-1.30) | <0.001 |
|  | Per 1-SD | 1.20 (1.15-1.25) | <0.001 | 1.20 (1.15-1.25) | <0.001 | 1.19 (1.14-1.25) | <0.001 |
|  | P for interaction |  | 0.057 |  | 0.060 |  | 0.019 |

*Notes:* Model 1 is adjusted for age and sex; Model 2 is adjusted for age, sex, smoking status, and alcohol consumption; and Model 3 is adjusted for age, sex, smoking status, alcohol consumption, diabetes, dyslipidemia, hypertension, platelet count, and monocyte count. Age was modeled using restricted cubic splines with 4 knots. HRs for cardiometabolic indices represent conditional effects at the mean level of WHtR due to mean-centering.

Abbreviations: AC, atherogenic coefficient; AIP, atherogenic index of plasma; CHG, cholesterol, high-density lipoprotein, and glucose index; CI, confidence interval; CRI-I, Castelli’s index-I; CRI-II, Castelli’s index-II; HR, hazard ratio; Non-HDL-C, non-high density lipoprotein cholesterol; RC, remnant cholesterol; RC/HDL-C, remnant cholesterol and high-density lipoprotein cholesterol ratio; TyG, triglyceride-glucose index; WHtR, waist-to-height ratio

Table S12. Associations of updated WHtR-based product indices with incident carotid plaque in time-dependent Cox models

| Exposure | Levels | Events | PY | Incidence Rate_1000PY | Model 1 | | Model 2 | | Model 3 | |
| --- | --- | --- | --- | --- | --- | --- | --- | --- | --- | --- |
|  |  |  |  |  | HR (95%CI) | *P* | HR (95%CI) | *P* | HR (95%CI) | *P* |
| RC–WHtR | Continuous |  |  |  | 1.01 (1.00-1.01) | 0.001 | 1.01 (1.00-1.01) | 0.001 | 1.00 (1.00-1.01) | 0.643 |
|  | Per 1-SD |  |  |  | 1.08 (1.03-1.14) | 0.001 | 1.08 (1.03-1.14) | 0.001 | 1.01 (0.96-1.08) | 0.643 |
|  | Q1 | 178 | 4369.390 | 40.740 | Reference | — | Reference | — | Reference | — |
|  | Q2 | 242 | 4341.742 | 55.740 | 1.07 (0.88-1.29) | 0.507 | 1.07 (0.88-1.30) | 0.501 | 1.03 (0.85-1.25) | 0.763 |
|  | Q3 | 290 | 3966.616 | 73.110 | 1.28 (1.06-1.55) | 0.010 | 1.28 (1.06-1.55) | 0.010 | 1.18 (0.98-1.43) | 0.084 |
|  | Q4 | 341 | 4098.836 | 83.190 | 1.32 (1.10-1.59) | 0.003 | 1.32 (1.10-1.59) | 0.003 | 1.10 (0.90-1.34) | 0.374 |
|  | *P* for trend |  |  |  |  | <0.001 |  | <0.001 |  | 0.218 |
| RC-HDL-C–WHtR | Continuous |  |  |  | 1.01 (0.99-1.04) | 0.273 | 1.02 (0.99-1.04) | 0.241 | 0.97 (0.88-1.06) | 0.470 |
|  | Per 1-SD |  |  |  | 1.01 (0.99-1.04) | 0.273 | 1.02 (0.99-1.04) | 0.241 | 0.97 (0.89-1.06) | 0.470 |
|  | Q1 | 173 | 4426.913 | 39.080 | Reference | — | Reference | — | Reference | — |
|  | Q2 | 241 | 4166.774 | 57.840 | 1.16 (0.95-1.41) | 0.148 | 1.16 (0.95-1.41) | 0.149 | 1.11 (0.91-1.35) | 0.317 |
|  | Q3 | 297 | 4038.830 | 73.540 | 1.30 (1.07-1.58) | 0.008 | 1.30 (1.07-1.58) | 0.007 | 1.17 (0.96-1.43) | 0.121 |
|  | Q4 | 340 | 4144.066 | 82.050 | 1.39 (1.15-1.69) | <0.001 | 1.39 (1.15-1.68) | <0.001 | 1.11 (0.89-1.37) | 0.360 |
|  | *P* for trend |  |  |  |  | <0.001 |  | <0.001 |  | 0.352 |
| Non-HDL-C–WHtR | Continuous |  |  |  | 1.52 (1.37-1.69) | <0.001 | 1.52 (1.37-1.69) | <0.001 | 1.42 (1.26-1.59) | <0.001 |
|  | Per 1-SD |  |  |  | 1.26 (1.19-1.34) | <0.001 | 1.26 (1.19-1.33) | <0.001 | 1.21 (1.13-1.29) | <0.001 |
|  | Q1 | 143 | 4047.631 | 35.330 | Reference | — | Reference | — | Reference | — |
|  | Q2 | 208 | 4260.461 | 48.820 | 1.12 (0.91-1.38) | 0.299 | 1.12 (0.91-1.38) | 0.298 | 1.10 (0.89-1.36) | 0.383 |
|  | Q3 | 302 | 4221.853 | 71.530 | 1.42 (1.16-1.73) | <0.001 | 1.42 (1.16-1.73) | <0.001 | 1.36 (1.10-1.66) | 0.004 |
|  | Q4 | 398 | 4246.638 | 93.720 | 1.74 (1.43-2.11) | <0.001 | 1.74 (1.43-2.11) | <0.001 | 1.58 (1.29-1.94) | <0.001 |
|  | *P* for trend |  |  |  |  | <0.001 |  | <0.001 |  | <0.001 |
| CHG–WHtR | Continuous |  |  |  | 1.66 (1.45-1.91) | <0.001 | 1.66 (1.45-1.91) | <0.001 | 1.37 (1.15-1.62) | <0.001 |
|  | Per 1-SD |  |  |  | 1.24 (1.17-1.31) | <0.001 | 1.24 (1.17-1.31) | <0.001 | 1.14 (1.06-1.22) | <0.001 |
|  | Q1 | 114 | 4030.989 | 28.280 | Reference | — | Reference | — | Reference | — |
|  | Q2 | 236 | 4366.488 | 54.050 | 1.34 (1.07-1.69) | 0.011 | 1.35 (1.07-1.69) | 0.010 | 1.30 (1.04-1.64) | 0.023 |
|  | Q3 | 284 | 4071.854 | 69.750 | 1.46 (1.16-1.84) | 0.001 | 1.46 (1.16-1.84) | 0.001 | 1.33 (1.05-1.69) | 0.017 |
|  | Q4 | 417 | 4307.252 | 96.810 | 1.87 (1.50-2.34) | <0.001 | 1.87 (1.50-2.34) | <0.001 | 1.50 (1.18-1.91) | 0.001 |
|  | *P* for trend |  |  |  |  | <0.001 |  | <0.001 |  | 0.003 |
| TyG–WHtR | Continuous |  |  |  | 1.32 (1.21-1.44) | <0.001 | 1.32 (1.21-1.44) | <0.001 | 1.14 (1.02-1.27) | 0.017 |
|  | Per 1-SD |  |  |  | 1.21 (1.14-1.28) | <0.001 | 1.21 (1.14-1.28) | <0.001 | 1.09 (1.02-1.17) | 0.017 |
|  | Q1 | 113 | 4187.966 | 26.980 | Reference | — | Reference | — | Reference | — |
|  | Q2 | 245 | 4222.976 | 58.020 | 1.46 (1.17-1.83) | <0.001 | 1.46 (1.17-1.83) | <0.001 | 1.40 (1.12-1.75) | 0.003 |
|  | Q3 | 304 | 4144.698 | 73.350 | 1.54 (1.23-1.92) | <0.001 | 1.54 (1.23-1.92) | <0.001 | 1.38 (1.09-1.74) | 0.007 |
|  | Q4 | 389 | 4220.944 | 92.160 | 1.84 (1.48-2.30) | <0.001 | 1.84 (1.48-2.29) | <0.001 | 1.43 (1.13-1.83) | 0.004 |
|  | *P* for trend |  |  |  |  | <0.001 |  | <0.001 |  | 0.025 |
| AIP–WHtR | Continuous |  |  |  | 2.14 (1.46-3.14) | <0.001 | 2.14 (1.46-3.14) | <0.001 | 1.16 (0.72-1.86) | 0.542 |
|  | Per 1-SD |  |  |  | 1.12 (1.06-1.19) | <0.001 | 1.12 (1.06-1.19) | <0.001 | 1.02 (0.95-1.10) | 0.542 |
|  | Q1 | 178 | 4319.309 | 41.210 | Reference | — | Reference | — | Reference | — |
|  | Q2 | 245 | 4240.357 | 57.780 | 1.19 (0.98-1.44) | 0.078 | 1.19 (0.98-1.44) | 0.075 | 1.15 (0.95-1.40) | 0.147 |
|  | Q3 | 287 | 4093.016 | 70.120 | 1.31 (1.08-1.58) | 0.005 | 1.31 (1.08-1.58) | 0.005 | 1.20 (0.99-1.45) | 0.065 |
|  | Q4 | 341 | 4123.902 | 82.690 | 1.46 (1.21-1.76) | <0.001 | 1.46 (1.21-1.76) | <0.001 | 1.15 (0.93-1.43) | 0.196 |
|  | *P* for trend |  |  |  |  | <0.001 |  | <0.001 |  | 0.194 |
| AC–WHtR | Continuous |  |  |  | 1.02 (1.01-1.04) | 0.008 | 1.02 (1.01-1.04) | 0.005 | 1.02 (1.00-1.03) | 0.056 |
|  | Per 1-SD |  |  |  | 1.03 (1.01-1.06) | 0.008 | 1.04 (1.01-1.06) | 0.005 | 1.02 (1.00-1.05) | 0.056 |
|  | Q1 | 135 | 4042.035 | 33.400 | Reference | — | Reference | — | Reference | — |
|  | Q2 | 244 | 4267.065 | 57.180 | 1.34 (1.09-1.66) | 0.006 | 1.34 (1.09-1.65) | 0.007 | 1.33 (1.07-1.64) | 0.009 |
|  | Q3 | 290 | 4171.803 | 69.510 | 1.48 (1.20-1.82) | <0.001 | 1.48 (1.20-1.82) | <0.001 | 1.39 (1.12-1.73) | 0.003 |
|  | Q4 | 382 | 4295.681 | 88.930 | 1.80 (1.46-2.21) | <0.001 | 1.79 (1.46-2.20) | <0.001 | 1.56 (1.24-1.96) | <0.001 |
|  | *P* for trend |  |  |  |  | <0.001 |  | <0.001 |  | <0.001 |
| CRI-I-WHtR | Continuous |  |  |  | 1.02 (1.01-1.04) | 0.007 | 1.02 (1.01-1.04) | 0.005 | 1.02 (1.00-1.03) | 0.049 |
|  | Per 1-SD |  |  |  | 1.04 (1.01-1.06) | 0.007 | 1.04 (1.01-1.06) | 0.005 | 1.03 (1.00-1.05) | 0.049 |
|  | Q1 | 132 | 4053.536 | 32.560 | Reference | — | Reference | — | Reference | — |
|  | Q2 | 239 | 4245.319 | 56.300 | 1.33 (1.07-1.64) | 0.010 | 1.32 (1.07-1.64) | 0.010 | 1.30 (1.05-1.61) | 0.015 |
|  | Q3 | 292 | 4155.382 | 70.270 | 1.48 (1.20-1.82) | <0.001 | 1.48 (1.20-1.82) | <0.001 | 1.39 (1.12-1.73) | 0.003 |
|  | Q4 | 388 | 4322.347 | 89.770 | 1.80 (1.47-2.22) | <0.001 | 1.80 (1.46-2.22) | <0.001 | 1.55 (1.23-1.95) | <0.001 |
|  | *P* for trend |  |  |  |  | <0.001 |  | <0.001 |  | <0.001 |
| CRI-II–WHtR | Continuous |  |  |  | 1.07 (1.03-1.12) | <0.001 | 1.08 (1.03-1.12) | <0.001 | 1.07 (1.02-1.11) | 0.002 |
|  | Per 1-SD |  |  |  | 1.05 (1.02-1.08) | <0.001 | 1.05 (1.02-1.08) | <0.001 | 1.05 (1.02-1.08) | 0.002 |
|  | Q1 | 134 | 3941.129 | 34.000 | Reference | — | Reference | — | Reference | — |
|  | Q2 | 239 | 4243.680 | 56.320 | 1.32 (1.07-1.63) | 0.010 | 1.32 (1.07-1.63) | 0.011 | 1.30 (1.05-1.61) | 0.015 |
|  | Q3 | 277 | 4281.642 | 64.690 | 1.42 (1.15-1.75) | 0.001 | 1.42 (1.15-1.75) | 0.001 | 1.35 (1.09-1.68) | 0.006 |
|  | Q4 | 401 | 4310.131 | 93.040 | 1.92 (1.57-2.35) | <0.001 | 1.91 (1.56-2.34) | <0.001 | 1.72 (1.38-2.15) | <0.001 |
|  | *P* for trend |  |  |  |  | <0.001 |  | <0.001 |  | <0.001 |

*Notes:* PY, person-years; Incidence rates_1000PY are expressed per 1,000 PY. Age was modeled using restricted cubic splines with 4 knots. Model 1 is adjusted for age and sex; Model 2 is adjusted for age, sex, smoking status, and alcohol consumption; and Model 3 is adjusted for age, sex, smoking status, alcohol consumption, diabetes, dyslipidemia, hypertension, platelet count, and monocyte count. Multiplicative composite indices are constructed by multiplying each cardiometabolic index and WHtR.

Abbreviation: AC, atherogenic coefficient; AIP, atherogenic index of plasma; CHG, cholesterol, high-density lipoprotein, and glucose index; CI, confidence interval; CRI-I, Castelli’s index-I; CRI-II, Castelli’s index-II; HR, hazard ratio; Non-HDL-C, non-high density lipoprotein cholesterol; RC, remnant cholesterol; RC/HDL-C, remnant cholesterol and high density lipoprotein cholesterol ratio; TyG, triglyceride-glucose index; WHtR, waist-to-height ratio

Table S13. Associations of 2-year time-weighted average WHtR-based product indices with incident carotid plaque

| Exposure | Levels | Events | PY | Incidence Rate_1000PY | Model 1 | | Model 2 | | Model 3 | |
| --- | --- | --- | --- | --- | --- | --- | --- | --- | --- | --- |
|  |  |  |  |  | HR (95%CI) | *P* | HR (95%CI) | *P* | HR (95%CI) | *P* |
| TWAvgRC–WHtR | Continuous |  |  |  | 1.01 (1.00-1.01) | 0.047 | 1.01 (1.00-1.01) | 0.047 | 1.00 (0.99-1.00) | 0.287 |
|  | Per 1-SD |  |  |  | 1.06 (1.00-1.12) | 0.047 | 1.06 (1.00-1.12) | 0.047 | 0.96 (0.90-1.03) | 0.287 |
|  | Q1 | 152 | 3792.506 | 40.080 | Reference | — | Reference | — | Reference | — |
|  | Q2 | 235 | 4127.470 | 56.940 | 1.06 (0.86-1.30) | 0.574 | 1.06 (0.86-1.30) | 0.605 | 0.99 (0.81-1.22) | 0.950 |
|  | Q3 | 291 | 4365.142 | 66.660 | 1.02 (0.83-1.24) | 0.882 | 1.01 (0.83-1.24) | 0.902 | 0.90 (0.74-1.11) | 0.337 |
|  | Q4 | 373 | 4491.466 | 83.050 | 1.18 (0.97-1.43) | 0.090 | 1.18 (0.97-1.43) | 0.098 | 0.91 (0.73-1.13) | 0.399 |
|  | *P* for trend |  |  |  |  | 0.087 |  | 0.091 |  | 0.270 |
| TWAvgRC/HDL-C–WHtR | Continuous |  |  |  | 1.02 (0.94-1.11) | 0.573 | 1.02 (0.94-1.10) | 0.656 | 0.90 (0.75-1.08) | 0.269 |
|  | Per 1-SD |  |  |  | 1.02 (0.96-1.09) | 0.573 | 1.01 (0.95-1.08) | 0.656 | 0.92 (0.79-1.07) | 0.269 |
|  | Q1 | 138 | 3888.724 | 35.490 | Reference | — | Reference | — | Reference | — |
|  | Q2 | 242 | 4213.218 | 57.440 | 1.20 (0.97-1.48) | 0.097 | 1.19 (0.97-1.47) | 0.102 | 1.12 (0.91-1.39) | 0.284 |
|  | Q3 | 323 | 4267.036 | 75.700 | 1.31 (1.07-1.61) | 0.009 | 1.30 (1.06-1.60) | 0.012 | 1.14 (0.92-1.41) | 0.224 |
|  | Q4 | 348 | 4407.606 | 78.950 | 1.34 (1.09-1.65) | 0.005 | 1.34 (1.09-1.65) | 0.005 | 0.98 (0.77-1.24) | 0.849 |
|  | *P* for trend |  |  |  |  | 0.005 |  | 0.005 |  | 0.715 |
| TWAvgNon-HDL-C–WHtR | Continuous |  |  |  | 1.58 (1.41-1.77) | <0.001 | 1.59 (1.42-1.78) | <0.001 | 1.49 (1.31-1.70) | <0.001 |
|  | Per 1-SD |  |  |  | 1.27 (1.20-1.35) | <0.001 | 1.28 (1.20-1.36) | <0.001 | 1.23 (1.15-1.32) | <0.001 |
|  | Q1 | 149 | 4351.264 | 34.240 | Reference | — | Reference | — | Reference | — |
|  | Q2 | 217 | 4341.124 | 49.990 | 1.12 (0.90-1.38) | 0.305 | 1.12 (0.90-1.38) | 0.306 | 1.10 (0.89-1.36) | 0.362 |
|  | Q3 | 302 | 4115.993 | 73.370 | 1.47 (1.20-1.79) | <0.001 | 1.46 (1.19-1.78) | <0.001 | 1.39 (1.14-1.71) | 0.001 |
|  | Q4 | 383 | 3968.203 | 96.520 | 1.84 (1.52-2.23) | <0.001 | 1.85 (1.52-2.24) | <0.001 | 1.71 (1.39-2.12) | <0.001 |
|  | *P* for trend |  |  |  |  | <0.001 |  | <0.001 |  | <0.001 |
| TWAvgCHG–WHtR | Continuous |  |  |  | 1.76 (1.50-2.07) | <0.001 | 1.77 (1.50-2.08) | <0.001 | 1.41 (1.16-1.71) | <0.001 |
|  | Per 1-SD |  |  |  | 1.26 (1.18-1.34) | <0.001 | 1.26 (1.18-1.35) | <0.001 | 1.15 (1.06-1.24) | <0.001 |
|  | Q1 | 111 | 4208.799 | 26.370 | Reference | — | Reference | — | Reference | — |
|  | Q2 | 248 | 4307.924 | 57.570 | 1.53 (1.22-1.92) | <0.001 | 1.50 (1.20-1.89) | <0.001 | 1.48 (1.17-1.86) | <0.001 |
|  | Q3 | 295 | 4198.825 | 70.260 | 1.52 (1.21-1.91) | <0.001 | 1.51 (1.20-1.90) | <0.001 | 1.40 (1.11-1.77) | 0.005 |
|  | Q4 | 397 | 4061.035 | 97.760 | 2.09 (1.67-2.62) | <0.001 | 2.08 (1.66-2.61) | <0.001 | 1.69 (1.32-2.16) | <0.001 |
|  | *P* for trend |  |  |  |  | <0.001 |  | <0.001 |  | <0.001 |
| TWAvgTyG–WHtR | Continuous |  |  |  | 1.33 (1.20-1.47) | <0.001 | 1.33 (1.20-1.48) | <0.001 | 1.12 (0.99-1.27) | 0.073 |
|  | Per 1-SD |  |  |  | 1.21 (1.13-1.29) | <0.001 | 1.21 (1.13-1.29) | <0.001 | 1.08 (0.99-1.17) | 0.073 |
|  | Q1 | 111 | 4187.685 | 26.510 | Reference | — | Reference | — | Reference | — |
|  | Q2 | 235 | 4243.201 | 55.380 | 1.45 (1.15-1.82) | 0.002 | 1.43 (1.14-1.80) | 0.002 | 1.41 (1.12-1.78) | 0.003 |
|  | Q3 | 317 | 4169.035 | 76.040 | 1.61 (1.29-2.02) | <0.001 | 1.60 (1.27-2.00) | <0.001 | 1.45 (1.15-1.82) | 0.002 |
|  | Q4 | 388 | 4176.661 | 92.900 | 1.89 (1.51-2.36) | <0.001 | 1.88 (1.51-2.36) | <0.001 | 1.49 (1.16-1.90) | 0.001 |
|  | *P* for trend |  |  |  |  | <0.001 |  | <0.001 |  | 0.009 |
| TWAvgAIP–WHtR | Continuous |  |  |  | 2.23 (1.47-3.40) | <0.001 | 2.23 (1.47-3.40) | <0.001 | 1.07 (0.62-1.86) | 0.802 |
|  | Per 1-SD |  |  |  | 1.13 (1.06-1.20) | <0.001 | 1.13 (1.06-1.20) | <0.001 | 1.01 (0.93-1.10) | 0.802 |
|  | Q1 | 170 | 4291.531 | 39.610 | Reference | — | Reference | — | Reference | — |
|  | Q2 | 249 | 4181.238 | 59.550 | 1.30 (1.07-1.58) | 0.009 | 1.28 (1.05-1.56) | 0.014 | 1.26 (1.03-1.53) | 0.023 |
|  | Q3 | 289 | 4097.073 | 70.540 | 1.32 (1.09-1.60) | 0.005 | 1.32 (1.09-1.60) | 0.005 | 1.20 (0.99-1.47) | 0.070 |
|  | Q4 | 343 | 4206.741 | 81.540 | 1.50 (1.24-1.82) | <0.001 | 1.50 (1.24-1.82) | <0.001 | 1.16 (0.92-1.46) | 0.210 |
|  | *P* for trend |  |  |  |  | <0.001 |  | <0.001 |  | 0.274 |
| TWAvgAC–WHtR | Continuous |  |  |  | 1.05 (1.02-1.07) | <0.001 | 1.04 (1.02-1.06) | <0.001 | 1.03 (1.00-1.06) | 0.084 |
|  | Per 1-SD |  |  |  | 1.06 (1.03-1.09) | <0.001 | 1.05 (1.02-1.08) | <0.001 | 1.04 (1.00-1.08) | 0.084 |
|  | Q1 | 154 | 4324.123 | 35.610 | Reference | — | Reference | — | Reference | — |
|  | Q2 | 228 | 4241.102 | 53.760 | 1.21 (0.98-1.48) | 0.076 | 1.21 (0.98-1.48) | 0.076 | 1.18 (0.96-1.45) | 0.117 |
|  | Q3 | 297 | 4266.235 | 69.620 | 1.40 (1.14-1.71) | 0.001 | 1.40 (1.14-1.70) | 0.001 | 1.36 (1.10-1.67) | 0.004 |
|  | Q4 | 372 | 3945.124 | 94.290 | 1.83 (1.51-2.23) | <0.001 | 1.85 (1.52-2.25) | <0.001 | 1.68 (1.33-2.11) | <0.001 |
|  | *P* for trend |  |  |  |  | <0.001 |  | <0.001 |  | <0.001 |
| TWAvgCRI-I–WHtR | Continuous |  |  |  | 1.05 (1.02-1.07) | <0.001 | 1.04 (1.02-1.06) | <0.001 | 1.03 (1.00-1.06) | 0.073 |
|  | Per 1-SD |  |  |  | 1.06 (1.03-1.09) | <0.001 | 1.05 (1.02-1.08) | <0.001 | 1.04 (1.00-1.08) | 0.073 |
|  | Q1 | 143 | 4298.552 | 33.270 | Reference | — | Reference | — | Reference | — |
|  | Q2 | 243 | 4262.273 | 57.010 | 1.32 (1.07-1.63) | 0.009 | 1.32 (1.07-1.63) | 0.009 | 1.29 (1.05-1.59) | 0.018 |
|  | Q3 | 292 | 4262.078 | 68.510 | 1.42 (1.16-1.75) | <0.001 | 1.42 (1.16-1.75) | <0.001 | 1.36 (1.10-1.69) | 0.004 |
|  | Q4 | 373 | 3953.680 | 94.340 | 1.89 (1.55-2.32) | <0.001 | 1.92 (1.56-2.35) | <0.001 | 1.70 (1.35-2.15) | <0.001 |
|  | *P* for trend |  |  |  |  | <0.001 |  | <0.001 |  | <0.001 |
| TWAvgCRI-II–WHtR | Continuous |  |  |  | 1.16 (1.11-1.21) | <0.001 | 1.15 (1.10-1.19) | <0.001 | 1.13 (1.08-1.19) | <0.001 |
|  | Per 1-SD |  |  |  | 1.09 (1.07-1.12) | <0.001 | 1.08 (1.06-1.11) | <0.001 | 1.08 (1.04-1.11) | <0.001 |
|  | Q1 | 162 | 4418.631 | 36.660 | Reference | — | Reference | — | Reference | — |
|  | Q2 | 230 | 4337.768 | 53.020 | 1.18 (0.96-1.44) | 0.111 | 1.18 (0.97-1.45) | 0.103 | 1.17 (0.96-1.44) | 0.126 |
|  | Q3 | 279 | 4203.258 | 66.380 | 1.35 (1.11-1.65) | 0.003 | 1.36 (1.11-1.65) | 0.003 | 1.34 (1.09-1.64) | 0.006 |
|  | Q4 | 380 | 3816.927 | 99.560 | 2.03 (1.67-2.46) | <0.001 | 2.05 (1.69-2.48) | <0.001 | 1.91 (1.55-2.37) | <0.001 |
|  | *P* for trend |  |  |  |  | <0.001 |  | <0.001 |  | <0.001 |

*Notes:* PY, person-years; Incidence rates_1000PY are expressed per 1,000 PY. Age was modeled using restricted cubic splines with 4 knots. Model 1 is adjusted for age and sex; Model 2 is adjusted for age, sex, smoking status, and alcohol consumption; and Model 3 is adjusted for age, sex, smoking status, alcohol consumption, diabetes, dyslipidemia, hypertension, platelet count, and monocyte count. Multiplicative composite indices are constructed by multiplying each cardiometabolic index and WHtR.

Abbreviation: AC, atherogenic coefficient; AIP, atherogenic index of plasma; CHG, cholesterol, high-density lipoprotein, and glucose index; CI, confidence interval; CRI-I, Castelli’s index-I; CRI-II, Castelli’s index-II; HR, hazard ratio; Non-HDL-C, non-high density lipoprotein cholesterol; RC, remnant cholesterol; RC/HDL-C, remnant cholesterol and high density lipoprotein cholesterol ratio; TWAvg, time-weighted average; TyG, triglyceride-glucose index; WHtR, waist-to-height ratio

Table S14. Time-dependent area under the curve of WHtR-based product indices for predicting carotid plaque

| Model | Year1 | Year2 | Year3 | Year4 | Year5 |
| --- | --- | --- | --- | --- | --- |
| RC–WHtR | 0.751 (0.720–0.782) | 0.751 (0.731–0.770) | 0.763 (0.746–0.779) | 0.731 (0.713–0.748) | 0.723 (0.698–0.749) |
| RC/HDL-C–WHtR | 0.752 (0.724–0.784) | 0.753 (0.737–0.771) | 0.763 (0.746–0.779) | 0.730 (0.712–0.751) | 0.721 (0.698–0.743) |
| Non-HDL-C–WHtR | 0.751 (0.724–0.780) | 0.754 (0.735–0.771) | 0.763 (0.747–0.781) | 0.737 (0.718–0.758) | 0.727 (0.704–0.751) |
| CHG–WHtR | 0.750 (0.721–0.779) | 0.752 (0.735–0.770) | 0.761 (0.745–0.779) | 0.732 (0.712–0.754) | 0.724 (0.700–0.750) |
| TyG–WHtR | 0.750 (0.726–0.781) | 0.750 (0.734–0.767) | 0.760 (0.744–0.778) | 0.729 (0.708–0.751) | 0.722 (0.697–0.750) |
| AIP–WHtR | 0.751 (0.723–0.778) | 0.752 (0.733–0.770) | 0.763 (0.746–0.781) | 0.731 (0.713–0.753) | 0.721 (0.698–0.744) |
| AC–WHtR | 0.751 (0.724–0.778) | 0.752 (0.733–0.771) | 0.763 (0.747–0.781) | 0.732 (0.714–0.754) | 0.724 (0.700–0.751) |
| CRI-I–WHtR | 0.752 (0.723–0.780) | 0.753 (0.734–0.771) | 0.762 (0.747–0.779) | 0.733 (0.714–0.755) | 0.725 (0.701–0.751) |
| CRI-II–WHtR | 0.752 (0.726–0.780) | 0.759 (0.740–0.778) | 0.770 (0.754–0.787) | 0.743 (0.724–0.763) | 0.733 (0.709–0.758) |

*Notes:* Multiplicative composite indices are constructed by multiplying each cardiometabolic index and WHtR.

Abbreviation: AC, atherogenic coefficient; AIP, atherogenic index of plasma; CHG, cholesterol, high-density lipoprotein, and glucose index; CRI-I, Castelli’s index-I; CRI-II, Castelli’s index-II; Non-HDL-C, non-high density lipoprotein cholesterol; RC, remnant cholesterol; RC/HDL-C, remnant cholesterol and high density lipoprotein cholesterol ratio; TyG, triglyceride-glucose index; WHtR, waist-to-height ratio

Table S15. Harrell’s C-index, Uno’s C-index, and integrated Brier score of WHtR-based product indices

| Model | Harrell’s C-index | Uno’s C-index | IBS |
| --- | --- | --- | --- |
| RC–WHtR | 0.733 (0.719–0.746) | 0.669 (0.663–0.679) | 0.099 |
| RC/HDL-C–WHtR | 0.734 (0.721–0.748) | 0.669 (0.663–0.678) | 0.099 |
| Non-HDL-C–WHtR | 0.737 (0.722–0.751) | 0.679 (0.670–0.691) | 0.098 |
| CHG–WHtR | 0.734 (0.722–0.747) | 0.674 (0.664–0.680) | 0.099 |
| TyG–WHtR | 0.733 (0.720–0.748) | 0.674 (0.665–0.680) | 0.099 |
| AIP–WHtR | 0.734 (0.721–0.748) | 0.672 (0.665–0.681) | 0.099 |
| AC–WHtR | 0.734 (0.720–0.749) | 0.669 (0.664–0.682) | 0.099 |
| CRI-I–WHtR | 0.735 (0.721–0.747) | 0.669 (0.664–0.680) | 0.099 |
| CRI-II–WHtR | 0.739 (0.726–0.753) | 0.673 (0.669–0.685) | 0.099 |

*Notes:* Multiplicative composite indices are constructed by multiplying each cardiometabolic index and WHtR.

Abbreviations: AC, atherogenic coefficient; AIP, atherogenic index of plasma; CHG, cholesterol, high-density lipoprotein, and glucose index; CRI-I, Castelli’s index-I; CRI-II, Castelli’s index-II; IBS, integrated Brier score; Non-HDL-C, non-high-density lipoprotein cholesterol; RC, remnant cholesterol; RC/HDL-C, remnant cholesterol to high-density lipoprotein cholesterol ratio; TyG, triglyceride–glucose index; WHtR, waist-to-height ratio

Table S16. Associations of cardiometabolic index–WHtR product terms with incident carotid plaque after additional adjustment for clinical covariates

| Exposure | Levels | Events | PY | IncidenceRate_1000PY | Model 4 | | Model 5 | | Model 6 | |
| --- | --- | --- | --- | --- | --- | --- | --- | --- | --- | --- |
|  |  |  |  |  | HR (95% CI) | *P* | HR (95% CI) | *P* | HR (95% CI) | *P* |
| RC–WHtR | Continuous |  |  |  | 1.00 (0.99-1.00) | 0.497 | 1.00 (0.99-1.00) | 0.460 | 1.00 (0.99-1.00) | 0.472 |
|  | Per 1-SD |  |  |  | 0.98 (0.92-1.04) | 0.497 | 0.98 (0.91-1.04) | 0.460 | 0.98 (0.91-1.04) | 0.472 |
|  | Q1 | 178 | 4355.638 | 40.870 | Reference | — | Reference | — | Reference | — |
|  | Q2 | 254 | 4719.432 | 53.820 | 0.97 (0.80-1.17) | 0.731 | 0.96 (0.79-1.17) | 0.716 | 0.96 (0.79-1.17) | 0.710 |
|  | Q3 | 331 | 5016.556 | 65.980 | 0.91 (0.76-1.10) | 0.347 | 0.91 (0.75-1.10) | 0.324 | 0.91 (0.75-1.10) | 0.320 |
|  | Q4 | 435 | 5175.371 | 84.050 | 0.94 (0.77-1.15) | 0.578 | 0.94 (0.77-1.16) | 0.569 | 0.94 (0.77-1.15) | 0.556 |
|  | *P* for trend |  |  |  |  | 0.528 |  | 0.509 |  | 0.497 |
| RC/HDL-C–WHtR | Continuous |  |  |  | 0.96 (0.82-1.11) | 0.557 | 0.94 (0.80-1.11) | 0.466 | 0.94 (0.81-1.11) | 0.478 |
|  | Per 1-SD |  |  |  | 0.98 (0.90-1.06) | 0.557 | 0.97 (0.89-1.05) | 0.466 | 0.97 (0.89-1.06) | 0.478 |
|  | Q1 | 165 | 4397.494 | 37.520 | Reference | — | Reference | — | Reference | — |
|  | Q2 | 285 | 4845.080 | 58.820 | 1.09 (0.90-1.33) | 0.364 | 1.07 (0.88-1.31) | 0.473 | 1.07 (0.88-1.30) | 0.478 |
|  | Q3 | 334 | 4966.549 | 67.250 | 0.95 (0.78-1.16) | 0.641 | 0.93 (0.76-1.14) | 0.481 | 0.93 (0.76-1.14) | 0.480 |
|  | Q4 | 414 | 5057.874 | 81.850 | 0.93 (0.75-1.16) | 0.532 | 0.89 (0.71-1.12) | 0.315 | 0.89 (0.71-1.12) | 0.310 |
|  | *P* for trend |  |  |  |  | 0.249 |  | 0.133 |  | 0.131 |
| Non-HDL-C–WHtR | Continuous |  |  |  | 1.44 (1.29-1.62) | <0.001 | 1.45 (1.29-1.63) | <0.001 | 1.45 (1.29-1.63) | <0.001 |
|  | Per 1-SD |  |  |  | 1.23 (1.15-1.31) | <0.001 | 1.23 (1.15-1.31) | <0.001 | 1.23 (1.15-1.31) | <0.001 |
|  | Q1 | 162 | 4947.165 | 32.750 | Reference | — | Reference | — | Reference | — |
|  | Q2 | 244 | 4961.411 | 49.180 | 1.14 (0.94-1.40) | 0.191 | 1.14 (0.93-1.39) | 0.208 | 1.14 (0.93-1.40) | 0.199 |
|  | Q3 | 350 | 4848.806 | 72.180 | 1.42 (1.17-1.72) | <0.001 | 1.41 (1.16-1.72) | <0.001 | 1.42 (1.16-1.72) | <0.001 |
|  | Q4 | 442 | 4509.616 | 98.010 | 1.77 (1.45-2.16) | <0.001 | 1.77 (1.44-2.17) | <0.001 | 1.78 (1.45-2.18) | <0.001 |
|  | *P* for trend |  |  |  |  | <0.001 |  | <0.001 |  | <0.001 |
| CHG–WHtR | Continuous |  |  |  | 1.37 (1.15-1.63) | <0.001 | 1.33 (1.10-1.60) | 0.003 | 1.33 (1.10-1.60) | 0.003 |
|  | Per 1-SD |  |  |  | 1.14 (1.06-1.23) | <0.001 | 1.13 (1.04-1.22) | 0.003 | 1.13 (1.04-1.22) | 0.003 |
|  | Q1 | 132 | 4783.908 | 27.590 | Reference | — | Reference | — | Reference | — |
|  | Q2 | 271 | 5016.098 | 54.030 | 1.23 (0.99-1.53) | 0.056 | 1.23 (0.99-1.52) | 0.064 | 1.22 (0.99-1.52) | 0.065 |
|  | Q3 | 342 | 4815.265 | 71.020 | 1.30 (1.05-1.62) | 0.018 | 1.27 (1.02-1.58) | 0.033 | 1.27 (1.02-1.58) | 0.034 |
|  | Q4 | 453 | 4651.727 | 97.380 | 1.48 (1.18-1.86) | <0.001 | 1.43 (1.13-1.81) | 0.003 | 1.43 (1.13-1.81) | 0.003 |
|  | *P* for trend |  |  |  |  | 0.001 |  | 0.005 |  | 0.005 |
| TyG–WHtR | Continuous |  |  |  | 1.14 (1.03-1.28) | 0.016 | 1.12 (1.00-1.26) | 0.050 | 1.12 (1.00-1.26) | 0.054 |
|  | Per 1-SD |  |  |  | 1.10 (1.02-1.18) | 0.016 | 1.08 (1.00-1.17) | 0.050 | 1.08 (1.00-1.17) | 0.054 |
|  | Q1 | 121 | 4813.550 | 25.140 | Reference | — | Reference | — | Reference | — |
|  | Q2 | 271 | 4888.054 | 55.440 | 1.43 (1.15-1.79) | 0.001 | 1.42 (1.14-1.77) | 0.002 | 1.42 (1.14-1.77) | 0.002 |
|  | Q3 | 365 | 4846.662 | 75.310 | 1.48 (1.19-1.85) | <0.001 | 1.45 (1.16-1.81) | 0.001 | 1.45 (1.16-1.81) | 0.001 |
|  | Q4 | 441 | 4718.731 | 93.460 | 1.55 (1.23-1.96) | <0.001 | 1.51 (1.19-1.93) | <0.001 | 1.51 (1.18-1.92) | <0.001 |
|  | *P* for trend |  |  |  |  | 0.002 |  | 0.007 |  | 0.007 |
| AIP–WHtR | Continuous |  |  |  | 1.52 (0.92-2.50) | 0.101 | 1.46 (0.87-2.45) | 0.150 | 1.46 (0.87-2.45) | 0.151 |
|  | Per 1-SD |  |  |  | 1.07 (0.99-1.15) | 0.101 | 1.06 (0.98-1.15) | 0.150 | 1.06 (0.98-1.15) | 0.151 |
|  | Q1 | 191 | 4932.912 | 38.720 | Reference | — | Reference | — | Reference | — |
|  | Q2 | 269 | 4797.621 | 56.070 | 1.25 (1.04-1.51) | 0.018 | 1.25 (1.04-1.51) | 0.018 | 1.25 (1.04-1.51) | 0.020 |
|  | Q3 | 332 | 4795.549 | 69.230 | 1.26 (1.04-1.51) | 0.016 | 1.24 (1.03-1.50) | 0.024 | 1.24 (1.03-1.50) | 0.025 |
|  | Q4 | 406 | 4740.915 | 85.640 | 1.25 (1.00-1.56) | 0.051 | 1.23 (0.98-1.55) | 0.072 | 1.23 (0.98-1.54) | 0.075 |
|  | *P* for trend |  |  |  |  | 0.053 |  | 0.078 |  | 0.082 |
| AC–WHtR | Continuous |  |  |  | 1.09 (1.04-1.14) | <0.001 | 1.08 (1.03-1.14) | 0.001 | 1.08 (1.03-1.14) | <0.001 |
|  | Per 1-SD |  |  |  | 1.08 (1.03-1.12) | <0.001 | 1.07 (1.03-1.12) | 0.001 | 1.07 (1.03-1.12) | <0.001 |
|  | Q1 | 163 | 4911.139 | 33.190 | Reference | — | Reference | — | Reference | — |
|  | Q2 | 275 | 4934.674 | 55.730 | 1.26 (1.03-1.53) | 0.024 | 1.25 (1.03-1.52) | 0.027 | 1.25 (1.03-1.53) | 0.026 |
|  | Q3 | 335 | 4883.086 | 68.600 | 1.36 (1.12-1.67) | 0.002 | 1.36 (1.11-1.67) | 0.003 | 1.36 (1.11-1.67) | 0.003 |
|  | Q4 | 425 | 4538.099 | 93.650 | 1.68 (1.35-2.10) | <0.001 | 1.64 (1.31-2.06) | <0.001 | 1.65 (1.32-2.07) | <0.001 |
|  | *P* for trend |  |  |  |  | <0.001 |  | <0.001 |  | <0.001 |
| CRI-I–WHtR | Continuous |  |  |  | 1.09 (1.04-1.14) | <0.001 | 1.08 (1.03-1.14) | 0.001 | 1.08 (1.03-1.14) | <0.001 |
|  | Per 1-SD |  |  |  | 1.08 (1.04-1.12) | <0.001 | 1.08 (1.03-1.12) | 0.001 | 1.08 (1.03-1.12) | <0.001 |
|  | Q1 | 161 | 4873.872 | 33.030 | Reference | — | Reference | — | Reference | — |
|  | Q2 | 270 | 4947.911 | 54.570 | 1.20 (0.98-1.46) | 0.080 | 1.18 (0.97-1.45) | 0.098 | 1.18 (0.97-1.45) | 0.099 |
|  | Q3 | 332 | 4905.278 | 67.680 | 1.29 (1.05-1.58) | 0.015 | 1.28 (1.04-1.58) | 0.018 | 1.28 (1.04-1.58) | 0.018 |
|  | Q4 | 435 | 4539.936 | 95.820 | 1.68 (1.35-2.09) | <0.001 | 1.63 (1.30-2.05) | <0.001 | 1.64 (1.31-2.06) | <0.001 |
|  | *P* for trend |  |  |  |  | <0.001 |  | <0.001 |  | <0.001 |
| CRI-II–WHtR | Continuous |  |  |  | 1.46 (1.31-1.63) | <0.001 | 1.43 (1.28-1.61) | <0.001 | 1.44 (1.28-1.61) | <0.001 |
|  | Per 1-SD |  |  |  | 1.21 (1.14-1.27) | <0.001 | 1.20 (1.13-1.27) | <0.001 | 1.20 (1.13-1.27) | <0.001 |
|  | Q1 | 165 | 5036.563 | 32.760 | Reference | — | Reference | — | Reference | — |
|  | Q2 | 290 | 5022.393 | 57.740 | 1.37 (1.13-1.67) | 0.001 | 1.38 (1.13-1.68) | 0.001 | 1.38 (1.13-1.67) | 0.001 |
|  | Q3 | 313 | 4842.695 | 64.630 | 1.44 (1.18-1.75) | <0.001 | 1.44 (1.18-1.76) | <0.001 | 1.44 (1.18-1.76) | <0.001 |
|  | Q4 | 430 | 4365.346 | 98.500 | 2.01 (1.63-2.46) | <0.001 | 1.96 (1.59-2.42) | <0.001 | 1.97 (1.60-2.43) | <0.001 |
|  | *P* for trend |  |  |  |  | <0.001 |  | <0.001 |  | <0.001 |

*Notes:* PY, person-years; Incidence rates_1000PY are expressed per 1,000 PY. Age was modeled using restricted cubic splines with 4 knots. Model 4 is adjusted for age, sex, smoking, alcohol consumption, diabetes, dyslipidemia, hypertension, platelet count, monocyte count, and medication use; Model 5 is adjusted for age, sex, smoking status, alcohol consumption, diabetes, dyslipidemia, hypertension, platelet count, monocyte count, ALT, albumin, serum uric acid, and serum creatinine; and Model 6 is adjusted for age, sex, smoking status, alcohol consumption, diabetes, dyslipidemia, hypertension, platelet count, monocyte count, medication use, ALT, albumin, serum uric acid, and serum creatinine. Multiplicative composite indices are constructed by multiplying each cardiometabolic index and WHtR.

Abbreviation: AC, atherogenic coefficient; AIP, atherogenic index of plasma; ALT, alanine aminotransferase; CHG, cholesterol, high-density lipoprotein, and glucose index; CI, confidence interval; CRI-I, Castelli’s index-I; CRI-II, Castelli’s index-II; HR, hazard ratio; Non-HDL-C, non-high density lipoprotein cholesterol; RC, remnant cholesterol; RC/HDL-C, remnant cholesterol and high density lipoprotein cholesterol ratio; TWAvg, time-weighted average; TyG, triglyceride-glucose index; WHtR, waist-to-height ratio

Table S17. Associations of cardiometabolic index–WHtR product terms with incident carotid plaque after excluding events within the first 2 years

| Exposure | Levels | Events | PY | Incidence Rate_1000PY | Model 1 | | Model 2 | | Model 3 | |
| --- | --- | --- | --- | --- | --- | --- | --- | --- | --- | --- |
|  |  |  |  |  | HR (95%CI) | *P* | HR (95%CI) | *P* | HR (95%CI) | *P* |
| RC–WHtR | Continuous |  |  |  | 1.01 (1.00-1.02) | 0.034 | 1.01 (1.00-1.02) | 0.034 | 1.00 (0.99-1.01) | 0.667 |
|  | Per 1-SD |  |  |  | 1.08 (1.01-1.16) | 0.034 | 1.08 (1.01-1.16) | 0.034 | 0.98 (0.90-1.07) | 0.667 |
|  | Q1 | 78 | 3527.514 | 22.110 | Reference | — | Reference | — | Reference | — |
|  | Q2 | 125 | 3838.127 | 32.570 | 1.06 (0.80-1.41) | 0.684 | 1.06 (0.80-1.41) | 0.676 | 1.00 (0.75-1.33) | 0.993 |
|  | Q3 | 145 | 4027.194 | 36.010 | 0.94 (0.71-1.24) | 0.659 | 0.94 (0.71-1.24) | 0.662 | 0.85 (0.64-1.13) | 0.264 |
|  | Q4 | 223 | 4134.321 | 53.940 | 1.27 (0.98-1.65) | 0.074 | 1.27 (0.98-1.65) | 0.073 | 0.99 (0.73-1.33) | 0.928 |
|  | *P* for trend |  |  |  |  | 0.056 |  | 0.056 |  | 0.735 |
| RC/HDL-C–WHtR | Continuous |  |  |  | 1.11 (0.98-1.25) | 0.111 | 1.10 (0.98-1.25) | 0.116 | 0.96 (0.77-1.19) | 0.680 |
|  | Per 1-SD |  |  |  | 1.06 (0.99-1.14) | 0.111 | 1.06 (0.99-1.14) | 0.116 | 0.97 (0.85-1.11) | 0.680 |
|  | Q1 | 69 | 3576.567 | 19.290 | Reference | — | Reference | — | Reference | — |
|  | Q2 | 141 | 3903.602 | 36.120 | 1.28 (0.96-1.71) | 0.097 | 1.28 (0.95-1.71) | 0.099 | 1.20 (0.90-1.61) | 0.223 |
|  | Q3 | 158 | 3970.612 | 39.790 | 1.16 (0.87-1.55) | 0.310 | 1.16 (0.87-1.55) | 0.317 | 1.01 (0.74-1.36) | 0.965 |
|  | Q4 | 203 | 4076.375 | 49.800 | 1.37 (1.03-1.81) | 0.030 | 1.36 (1.03-1.81) | 0.032 | 0.94 (0.67-1.31) | 0.703 |
|  | *P* for trend |  |  |  |  | 0.073 |  | 0.076 |  | 0.324 |
| Non-HDL-C–WHtR | Continuous |  |  |  | 1.60 (1.39-1.85) | <0.001 | 1.60 (1.39-1.85) | <0.001 | 1.48 (1.25-1.75) | <0.001 |
|  | Per 1-SD |  |  |  | 1.29 (1.19-1.39) | <0.001 | 1.29 (1.19-1.39) | <0.001 | 1.23 (1.13-1.35) | <0.001 |
|  | Q1 | 78 | 3883.791 | 20.080 | Reference | — | Reference | — | Reference | — |
|  | Q2 | 114 | 4002.690 | 28.480 | 1.05 (0.78-1.40) | 0.765 | 1.04 (0.78-1.39) | 0.785 | 1.04 (0.77-1.39) | 0.810 |
|  | Q3 | 158 | 3867.972 | 40.850 | 1.39 (1.05-1.83) | 0.020 | 1.39 (1.05-1.83) | 0.021 | 1.34 (1.01-1.78) | 0.044 |
|  | Q4 | 221 | 3772.703 | 58.580 | 1.93 (1.48-2.52) | <0.001 | 1.93 (1.48-2.52) | <0.001 | 1.79 (1.34-2.40) | <0.001 |
|  | *P* for trend |  |  |  |  | <0.001 |  | <0.001 |  | <0.001 |
| CHG–WHtR | Continuous |  |  |  | 1.79 (1.44-2.23) | <0.001 | 1.79 (1.43-2.23) | <0.001 | 1.42 (1.09-1.85) | 0.009 |
|  | Per 1-SD |  |  |  | 1.27 (1.16-1.39) | <0.001 | 1.27 (1.16-1.39) | <0.001 | 1.16 (1.04-1.29) | 0.009 |
|  | Q1 | 62 | 3824.169 | 16.210 | Reference | — | Reference | — | Reference | — |
|  | Q2 | 128 | 3924.260 | 32.620 | 1.35 (0.99-1.84) | 0.061 | 1.34 (0.99-1.84) | 0.062 | 1.30 (0.95-1.78) | 0.099 |
|  | Q3 | 160 | 3934.584 | 40.670 | 1.42 (1.04-1.93) | 0.027 | 1.41 (1.04-1.93) | 0.029 | 1.31 (0.96-1.81) | 0.091 |
|  | Q4 | 221 | 3844.143 | 57.490 | 1.96 (1.44-2.66) | <0.001 | 1.96 (1.44-2.65) | <0.001 | 1.58 (1.13-2.20) | 0.007 |
|  | *P* for trend |  |  |  |  | <0.001 |  | <0.001 |  | 0.011 |
| TyG–WHtR | Continuous |  |  |  | 1.38 (1.20-1.58) | <0.001 | 1.38 (1.20-1.58) | <0.001 | 1.17 (0.99-1.38) | 0.059 |
|  | Per 1-SD |  |  |  | 1.24 (1.13-1.36) | <0.001 | 1.24 (1.13-1.36) | <0.001 | 1.11 (1.00-1.24) | 0.059 |
|  | Q1 | 62 | 3863.500 | 16.050 | Reference | — | Reference | — | Reference | — |
|  | Q2 | 124 | 3905.213 | 31.750 | 1.43 (1.05-1.95) | 0.024 | 1.43 (1.05-1.95) | 0.025 | 1.39 (1.02-1.90) | 0.039 |
|  | Q3 | 175 | 3910.219 | 44.750 | 1.64 (1.21-2.22) | 0.001 | 1.63 (1.20-2.21) | 0.002 | 1.51 (1.11-2.06) | 0.009 |
|  | Q4 | 210 | 3848.224 | 54.570 | 2.00 (1.48-2.70) | <0.001 | 2.00 (1.48-2.70) | <0.001 | 1.60 (1.14-2.23) | 0.006 |
|  | *P* for trend |  |  |  |  | <0.001 |  | <0.001 |  | 0.011 |
| AIP–WHtR | Continuous |  |  |  | 2.66 (1.55-4.58) | <0.001 | 2.65 (1.54-4.57) | <0.001 | 1.26 (0.61-2.63) | 0.530 |
|  | Per 1-SD |  |  |  | 1.16 (1.07-1.26) | <0.001 | 1.16 (1.07-1.26) | <0.001 | 1.04 (0.93-1.16) | 0.530 |
|  | Q1 | 101 | 3988.447 | 25.320 | Reference | — | Reference | — | Reference | — |
|  | Q2 | 129 | 3837.453 | 33.620 | 1.29 (0.99-1.67) | 0.059 | 1.29 (0.99-1.67) | 0.060 | 1.25 (0.96-1.63) | 0.090 |
|  | Q3 | 151 | 3883.563 | 38.880 | 1.24 (0.96-1.60) | 0.098 | 1.24 (0.96-1.60) | 0.104 | 1.14 (0.87-1.49) | 0.334 |
|  | Q4 | 190 | 3817.692 | 49.770 | 1.57 (1.22-2.01) | <0.001 | 1.56 (1.22-2.01) | <0.001 | 1.16 (0.84-1.60) | 0.362 |
|  | *P* for trend |  |  |  |  | <0.001 |  | 0.001 |  | 0.457 |
| AC–WHtR | Continuous |  |  |  | 1.15 (1.09-1.21) | <0.001 | 1.15 (1.09-1.21) | <0.001 | 1.10 (1.02-1.19) | 0.010 |
|  | Per 1-SD |  |  |  | 1.14 (1.09-1.19) | <0.001 | 1.14 (1.08-1.19) | <0.001 | 1.10 (1.02-1.18) | 0.010 |
|  | Q1 | 78 | 3911.773 | 19.940 | Reference | — | Reference | — | Reference | — |
|  | Q2 | 132 | 3911.097 | 33.750 | 1.32 (1.00-1.76) | 0.053 | 1.32 (1.00-1.76) | 0.054 | 1.28 (0.96-1.71) | 0.089 |
|  | Q3 | 152 | 3920.247 | 38.770 | 1.39 (1.05-1.84) | 0.022 | 1.39 (1.05-1.84) | 0.023 | 1.32 (0.98-1.77) | 0.064 |
|  | Q4 | 209 | 3784.038 | 55.230 | 1.93 (1.47-2.54) | <0.001 | 1.93 (1.46-2.53) | <0.001 | 1.65 (1.20-2.26) | 0.002 |
|  | *P* for trend |  |  |  |  | <0.001 |  | <0.001 |  | 0.003 |
| CRI-I–WHtR | Continuous |  |  |  | 1.15 (1.09-1.21) | <0.001 | 1.15 (1.09-1.21) | <0.001 | 1.10 (1.02-1.19) | 0.009 |
|  | Per 1-SD |  |  |  | 1.14 (1.09-1.20) | <0.001 | 1.14 (1.09-1.20) | <0.001 | 1.10 (1.02-1.18) | 0.009 |
|  | Q1 | 79 | 3870.861 | 20.410 | Reference | — | Reference | — | Reference | — |
|  | Q2 | 125 | 3938.378 | 31.740 | 1.15 (0.86-1.53) | 0.346 | 1.14 (0.86-1.52) | 0.355 | 1.10 (0.82-1.46) | 0.538 |
|  | Q3 | 164 | 3942.849 | 41.590 | 1.33 (1.00-1.75) | 0.048 | 1.32 (1.00-1.75) | 0.051 | 1.25 (0.93-1.67) | 0.137 |
|  | Q4 | 203 | 3775.068 | 53.770 | 1.76 (1.33-2.32) | <0.001 | 1.75 (1.33-2.31) | <0.001 | 1.46 (1.06-2.01) | 0.021 |
|  | *P* for trend |  |  |  |  | <0.001 |  | <0.001 |  | 0.012 |
| CRI-II–WHtR | Continuous |  |  |  | 1.67 (1.44-1.93) | <0.001 | 1.67 (1.44-1.93) | <0.001 | 1.53 (1.28-1.83) | <0.001 |
|  | Per 1-SD |  |  |  | 1.29 (1.20-1.38) | <0.001 | 1.29 (1.20-1.38) | <0.001 | 1.23 (1.13-1.35) | <0.001 |
|  | Q1 | 82 | 3995.760 | 20.520 | Reference | — | Reference | — | Reference | — |
|  | Q2 | 133 | 3968.562 | 33.510 | 1.33 (1.00-1.75) | 0.046 | 1.33 (1.00-1.75) | 0.047 | 1.30 (0.98-1.72) | 0.065 |
|  | Q3 | 152 | 3900.063 | 38.970 | 1.44 (1.09-1.89) | 0.010 | 1.44 (1.09-1.89) | 0.010 | 1.43 (1.07-1.90) | 0.014 |
|  | Q4 | 204 | 3662.771 | 55.700 | 2.15 (1.65-2.82) | <0.001 | 2.15 (1.65-2.81) | <0.001 | 1.95 (1.45-2.61) | <0.001 |
|  | *P* for trend |  |  |  |  | <0.001 |  | <0.001 |  | <0.001 |

*Notes:* PY, person-years; Incidence rates_1000PY are expressed per 1,000 PY. Age was modeled using restricted cubic splines with 4 knots. Model 1 is adjusted for age and sex; Model 2 is adjusted for age, sex, smoking status, and alcohol consumption; and Model 3 is adjusted for age, sex, smoking status, alcohol consumption, diabetes, dyslipidemia, hypertension, platelet count, and monocyte count. Multiplicative composite indices are constructed by multiplying each cardiometabolic index and WHtR

Abbreviation: AC, atherogenic coefficient; AIP, atherogenic index of plasma; CHG, cholesterol, high-density lipoprotein, and glucose index; CI, confidence interval; CRI-I, Castelli’s index-I; CRI-II, Castelli’s index-II; HR, hazard ratio; Non-HDL-C, non-high density lipoprotein cholesterol; RC, remnant cholesterol; RC/HDL-C, remnant cholesterol and high density lipoprotein cholesterol ratio; TyG, triglyceride-glucose index; WHtR, waist-to-height ratio

Table S18. Associations of cardiometabolic index–WHtR product terms with incident carotid plaque in complete-case analysis

| Exposure | Levels | Events | PY | Incidence Rate_1000PY | Model 1 |  | Model 2 |  | Model 3 |  |
| --- | --- | --- | --- | --- | --- | --- | --- | --- | --- | --- |
|  |  |  |  |  | HR (95%CI) | *P* | HR (95%CI) | *P* | HR (95%CI) | *P* |
| RC–WHtR | Continuous |  |  |  | 1.01 (1.00-1.01) | 0.005 | 1.01 (1.00-1.01) | 0.005 | 1.00 (0.99-1.00) | 0.589 |
|  | Per 1-SD |  |  |  | 1.08 (1.02-1.14) | 0.005 | 1.08 (1.02-1.14) | 0.005 | 0.98 (0.92-1.05) | 0.589 |
|  | Q1 | 167 | 3941.732 | 42.367 | Reference | — | Reference | — | Reference | — |
|  | Q2 | 228 | 4243.622 | 53.728 | 0.99 (0.81-1.21) | 0.913 | 0.99 (0.81-1.21) | 0.908 | 0.94 (0.77-1.15) | 0.535 |
|  | Q3 | 299 | 4550.968 | 65.700 | 1.02 (0.84-1.23) | 0.858 | 1.02 (0.84-1.23) | 0.866 | 0.92 (0.75-1.12) | 0.385 |
|  | Q4 | 405 | 4533.562 | 89.334 | 1.30 (1.08-1.56) | 0.005 | 1.30 (1.08-1.56) | 0.005 | 1.02 (0.83-1.26) | 0.817 |
|  | *P* for trend |  |  |  |  | <0.001 |  | <0.001 |  | 0.722 |
| RC/HDL-C–WHtR | Continuous |  |  |  | 1.07 (0.99-1.17) | 0.103 | 1.07 (0.99-1.17) | 0.102 | 0.93 (0.79-1.10) | 0.383 |
|  | Per 1-SD |  |  |  | 1.04 (0.99-1.09) | 0.103 | 1.04 (0.99-1.09) | 0.102 | 0.96 (0.87-1.05) | 0.383 |
|  | Q1 | 157 | 3992.943 | 39.319 | Reference | — | Reference | — | Reference | — |
|  | Q2 | 251 | 4354.566 | 57.641 | 1.12 (0.91-1.37) | 0.276 | 1.12 (0.91-1.37) | 0.274 | 1.07 (0.87-1.31) | 0.531 |
|  | Q3 | 309 | 4459.180 | 69.295 | 1.13 (0.93-1.37) | 0.229 | 1.13 (0.93-1.37) | 0.229 | 0.98 (0.80-1.20) | 0.835 |
|  | Q4 | 382 | 4463.194 | 85.589 | 1.36 (1.12-1.65) | 0.002 | 1.36 (1.12-1.65) | 0.002 | 0.99 (0.79-1.24) | 0.935 |
|  | *P* for trend |  |  |  |  | 0.001 |  | <0.001 |  | 0.706 |
| Non-HDL-C–WHtR | Continuous |  |  |  | 1.61 (1.46-1.79) | <0.001 | 1.61 (1.46-1.79) | <0.001 | 1.49 (1.33-1.68) | <0.001 |
|  | Per 1-SD |  |  |  | 1.30 (1.23-1.38) | <0.001 | 1.30 (1.23-1.38) | <0.001 | 1.25 (1.17-1.33) | <0.001 |
|  | Q1 | 148 | 4536.479 | 32.624 | Reference | — | Reference | — | Reference | — |
|  | Q2 | 225 | 4410.944 | 51.009 | 1.23 (1.00-1.52) | 0.051 | 1.23 (1.00-1.52) | 0.052 | 1.22 (0.99-1.50) | 0.067 |
|  | Q3 | 326 | 4338.443 | 75.142 | 1.60 (1.32-1.95) | <0.001 | 1.60 (1.32-1.95) | <0.001 | 1.53 (1.25-1.87) | <0.001 |
|  | Q4 | 400 | 3984.019 | 100.401 | 2.08 (1.71-2.52) | <0.001 | 2.08 (1.71-2.51) | <0.001 | 1.88 (1.53-2.32) | <0.001 |
|  | *P* for trend |  |  |  |  | <0.001 |  | <0.001 |  | <0.001 |
| CHG–WHtR | Continuous |  |  |  | 1.72 (1.48-2.00) | <0.001 | 1.72 (1.48-2.00) | <0.001 | 1.37 (1.14-1.64) | <0.001 |
|  | Per 1-SD |  |  |  | 1.25 (1.18-1.34) | <0.001 | 1.25 (1.18-1.34) | <0.001 | 1.14 (1.05-1.23) | <0.001 |
|  | Q1 | 123 | 4394.009 | 27.993 | Reference | — | Reference | — | Reference | — |
|  | Q2 | 251 | 4476.560 | 56.070 | 1.38 (1.11-1.72) | 0.004 | 1.38 (1.11-1.72) | 0.004 | 1.33 (1.06-1.66) | 0.012 |
|  | Q3 | 319 | 4282.176 | 74.495 | 1.54 (1.24-1.92) | <0.001 | 1.54 (1.24-1.92) | <0.001 | 1.39 (1.11-1.74) | 0.004 |
|  | Q4 | 406 | 4117.139 | 98.612 | 1.94 (1.56-2.41) | <0.001 | 1.94 (1.56-2.41) | <0.001 | 1.53 (1.21-1.94) | <0.001 |
|  | *P* for trend |  |  |  |  | <0.001 |  | <0.001 |  | 0.001 |
| TyG–WHtR | Continuous |  |  |  | 1.35 (1.23-1.48) | <0.001 | 1.35 (1.23-1.48) | <0.001 | 1.15 (1.03-1.29) | 0.015 |
|  | Per 1-SD |  |  |  | 1.23 (1.15-1.31) | <0.001 | 1.23 (1.15-1.31) | <0.001 | 1.10 (1.02-1.19) | 0.015 |
|  | Q1 | 115 | 4406.616 | 26.097 | Reference | — | Reference | — | Reference | — |
|  | Q2 | 251 | 4394.723 | 57.114 | 1.51 (1.21-1.90) | <0.001 | 1.52 (1.21-1.90) | <0.001 | 1.44 (1.15-1.81) | 0.001 |
|  | Q3 | 332 | 4308.725 | 77.053 | 1.72 (1.37-2.15) | <0.001 | 1.72 (1.38-2.15) | <0.001 | 1.55 (1.24-1.95) | <0.001 |
|  | Q4 | 401 | 4159.821 | 96.398 | 2.05 (1.65-2.56) | <0.001 | 2.06 (1.65-2.56) | <0.001 | 1.60 (1.26-2.04) | <0.001 |
|  | *P* for trend |  |  |  |  | <0.001 |  | <0.001 |  | <0.001 |
| AIP–WHtR | Continuous |  |  |  | 2.91 (1.98-4.28) | <0.001 | 2.92 (1.99-4.29) | <0.001 | 1.58 (0.94-2.65) | 0.083 |
|  | Per 1-SD |  |  |  | 1.18 (1.11-1.25) | <0.001 | 1.18 (1.11-1.25) | <0.001 | 1.07 (0.99-1.16) | 0.083 |
|  | Q1 | 170 | 4465.545 | 38.069 | Reference | — | Reference | — | Reference | — |
|  | Q2 | 251 | 4332.826 | 57.930 | 1.39 (1.15-1.69) | <0.001 | 1.40 (1.15-1.70) | <0.001 | 1.36 (1.12-1.65) | 0.002 |
|  | Q3 | 312 | 4258.142 | 73.271 | 1.50 (1.24-1.81) | <0.001 | 1.50 (1.24-1.82) | <0.001 | 1.37 (1.12-1.66) | 0.002 |
|  | Q4 | 366 | 4213.371 | 86.866 | 1.69 (1.40-2.04) | <0.001 | 1.70 (1.41-2.05) | <0.001 | 1.27 (1.01-1.61) | 0.044 |
|  | *P* for trend |  |  |  |  | <0.001 |  | <0.001 |  | 0.036 |
| AC–WHtR | Continuous |  |  |  | 1.11 (1.08-1.15) | <0.001 | 1.11 (1.08-1.15) | <0.001 | 1.08 (1.03-1.13) | <0.001 |
|  | Per 1-SD |  |  |  | 1.10 (1.07-1.13) | <0.001 | 1.10 (1.07-1.13) | <0.001 | 1.07 (1.03-1.12) | <0.001 |
|  | Q1 | 162 | 4484.887 | 36.121 | Reference | — | Reference | — | Reference | — |
|  | Q2 | 236 | 4410.901 | 53.504 | 1.21 (0.99-1.48) | 0.069 | 1.21 (0.99-1.48) | 0.067 | 1.18 (0.96-1.45) | 0.110 |
|  | Q3 | 314 | 4375.673 | 71.760 | 1.45 (1.19-1.76) | <0.001 | 1.45 (1.19-1.76) | <0.001 | 1.37 (1.12-1.69) | 0.002 |
|  | Q4 | 387 | 3998.422 | 96.788 | 1.92 (1.58-2.33) | <0.001 | 1.93 (1.59-2.33) | <0.001 | 1.71 (1.36-2.14) | <0.001 |
|  | *P* for trend |  |  |  |  | <0.001 |  | <0.001 |  | <0.001 |
| CRI-I–WHtR | Continuous |  |  |  | 1.11 (1.08-1.15) | <0.001 | 1.11 (1.08-1.15) | <0.001 | 1.08 (1.03-1.13) | <0.001 |
|  | Per 1-SD |  |  |  | 1.11 (1.07-1.14) | <0.001 | 1.11 (1.07-1.14) | <0.001 | 1.08 (1.03-1.12) | <0.001 |
|  | Q1 | 157 | 4444.077 | 35.328 | Reference | — | Reference | — | Reference | — |
|  | Q2 | 243 | 4444.636 | 54.673 | 1.20 (0.98-1.48) | 0.073 | 1.21 (0.99-1.48) | 0.069 | 1.16 (0.95-1.43) | 0.148 |
|  | Q3 | 309 | 4363.413 | 70.816 | 1.38 (1.13-1.69) | 0.001 | 1.39 (1.14-1.69) | 0.001 | 1.30 (1.05-1.60) | 0.014 |
|  | Q4 | 390 | 4017.757 | 97.069 | 1.88 (1.55-2.29) | <0.001 | 1.89 (1.55-2.30) | <0.001 | 1.62 (1.29-2.04) | <0.001 |
|  | *P* for trend |  |  |  |  | <0.001 |  | <0.001 |  | <0.001 |
| CRI-II–WHtR | Continuous |  |  |  | 1.55 (1.42-1.69) | <0.001 | 1.55 (1.42-1.70) | <0.001 | 1.45 (1.30-1.62) | <0.001 |
|  | Per 1-SD |  |  |  | 1.25 (1.19-1.30) | <0.001 | 1.25 (1.19-1.30) | <0.001 | 1.21 (1.14-1.28) | <0.001 |
|  | Q1 | 164 | 4596.915 | 35.676 | Reference | — | Reference | — | Reference | — |
|  | Q2 | 257 | 4508.695 | 57.001 | 1.30 (1.07-1.59) | 0.008 | 1.31 (1.07-1.59) | 0.008 | 1.27 (1.04-1.55) | 0.018 |
|  | Q3 | 288 | 4314.711 | 66.748 | 1.44 (1.19-1.76) | <0.001 | 1.45 (1.19-1.76) | <0.001 | 1.40 (1.15-1.71) | 0.001 |
|  | Q4 | 390 | 3849.563 | 101.310 | 2.12 (1.75-2.56) | <0.001 | 2.13 (1.76-2.57) | <0.001 | 1.91 (1.54-2.35) | <0.001 |
|  | *P* for trend |  |  |  |  | <0.001 |  | <0.001 |  | <0.001 |

*Notes:* PY, person-years; Incidence rates_1000PY are expressed per 1,000 PY. Age was modeled using restricted cubic splines with 4 knots. Model 1 is adjusted for age and sex; Model 2 is adjusted for age, sex, smoking status, and alcohol consumption; and Model 3 is adjusted for age, sex, smoking status, alcohol consumption, diabetes, dyslipidemia, hypertension, platelet count, and monocyte count. Multiplicative composite indices are constructed by multiplying each cardiometabolic index and WHtR

Abbreviation: AC, atherogenic coefficient; AIP, atherogenic index of plasma; CHG, cholesterol, high-density lipoprotein, and glucose index; CI, confidence interval; CRI-I, Castelli’s index-I; CRI-II, Castelli’s index-II; HR, hazard ratio; Non-HDL-C, non-high density lipoprotein cholesterol; RC, remnant cholesterol; RC/HDL-C, remnant cholesterol and high density lipoprotein cholesterol ratio; TyG, triglyceride-glucose index; WHtR, waist-to-height ratio


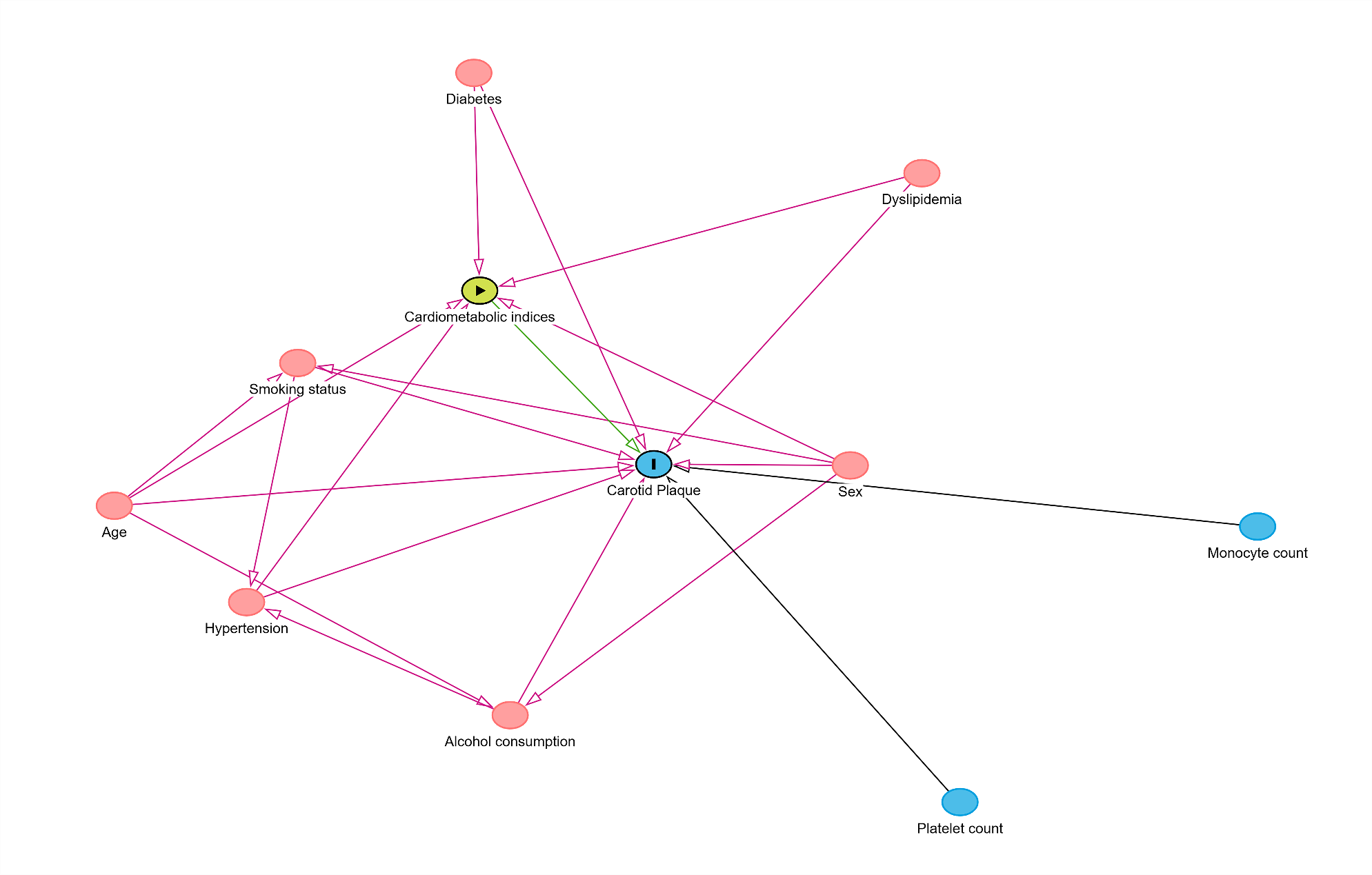


Figure S1. Directed acyclic graph illustrating the assumed relationships between cardiometabolic indices and carotid plaque.

*Note:* The green node with a black triangle represents the exposure, i.e., cardiometabolic indices. The blue node with a black vertical bar represents the outcome, carotid plaque. Light green nodes represent ancestors of the exposure, blue nodes represent ancestors of the outcome, and pink nodes represent common ancestors of both the exposure and the outcome. Directed arrows denote assumed direct relationships between variables. Green arrows indicate paths from the exposure to the outcome. Blue arrows indicate paths from ancestors of the outcome to the outcome. Pink arrows indicate paths involving common ancestors of both the exposure and outcome, representing potential confounding structures


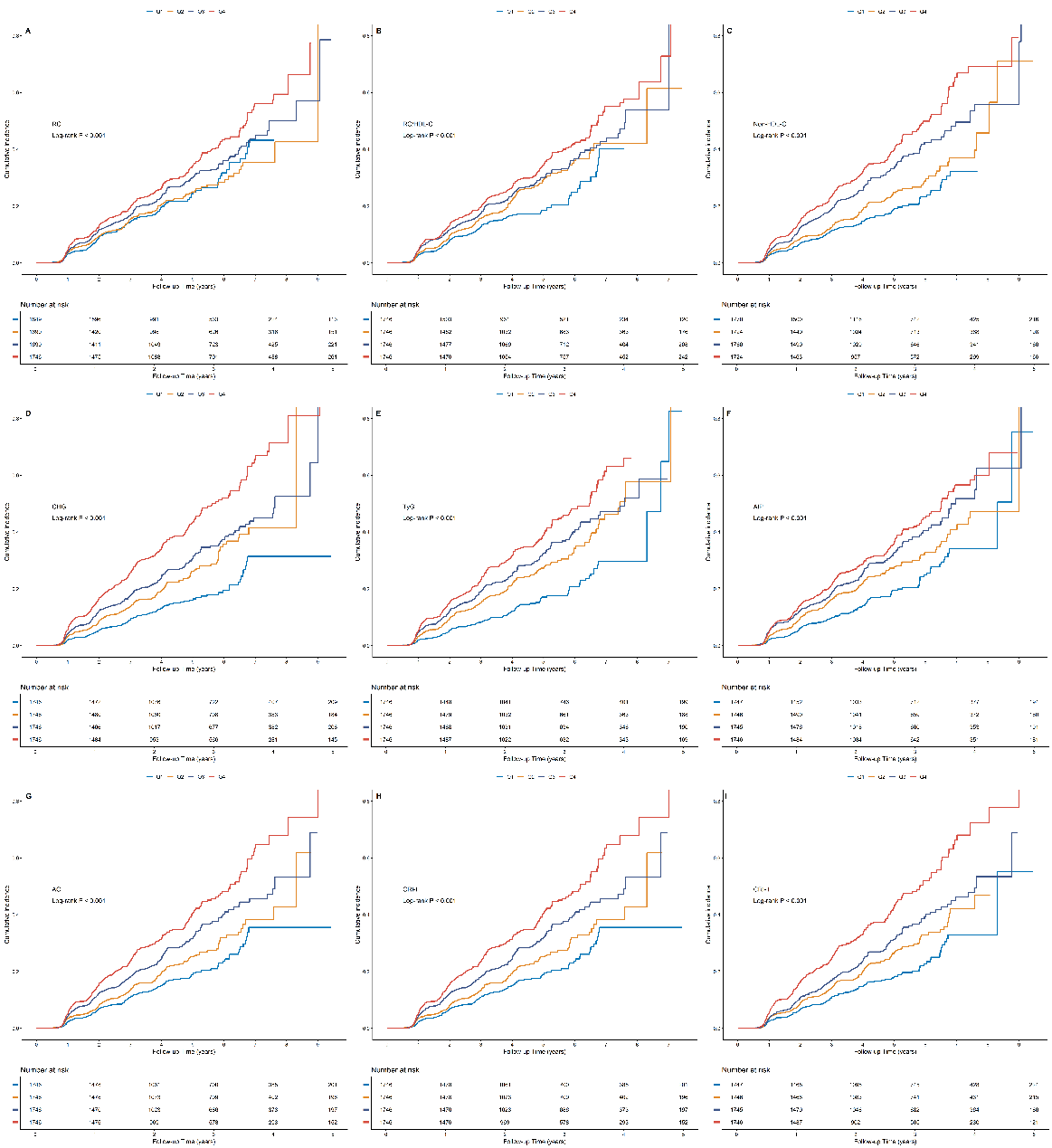


Figure S2. Kaplan–Meier plots of cumulative incidence of carotid plaque: (A) RC; (B) RC/HDL-C; (C) Non-HDL-C; (D) CHG; (E) TyG; (F) AIP; (G) AC; (H) CRI-I; (I) CRI-II.

Abbreviation: AC, atherogenic coefficient; AIP, atherogenic index of plasma; CHG, cholesterol, high-density lipoprotein, and glucose index; CRI-I, Castelli’s index-I; CRI-II, Castelli’s index-II; Non-HDL-C, non-high density lipoprotein cholesterol; RC, remnant cholesterol; RC/HDL-C, remnant cholesterol and high density lipoprotein cholesterol ratio; TyG, triglyceride-glucose index


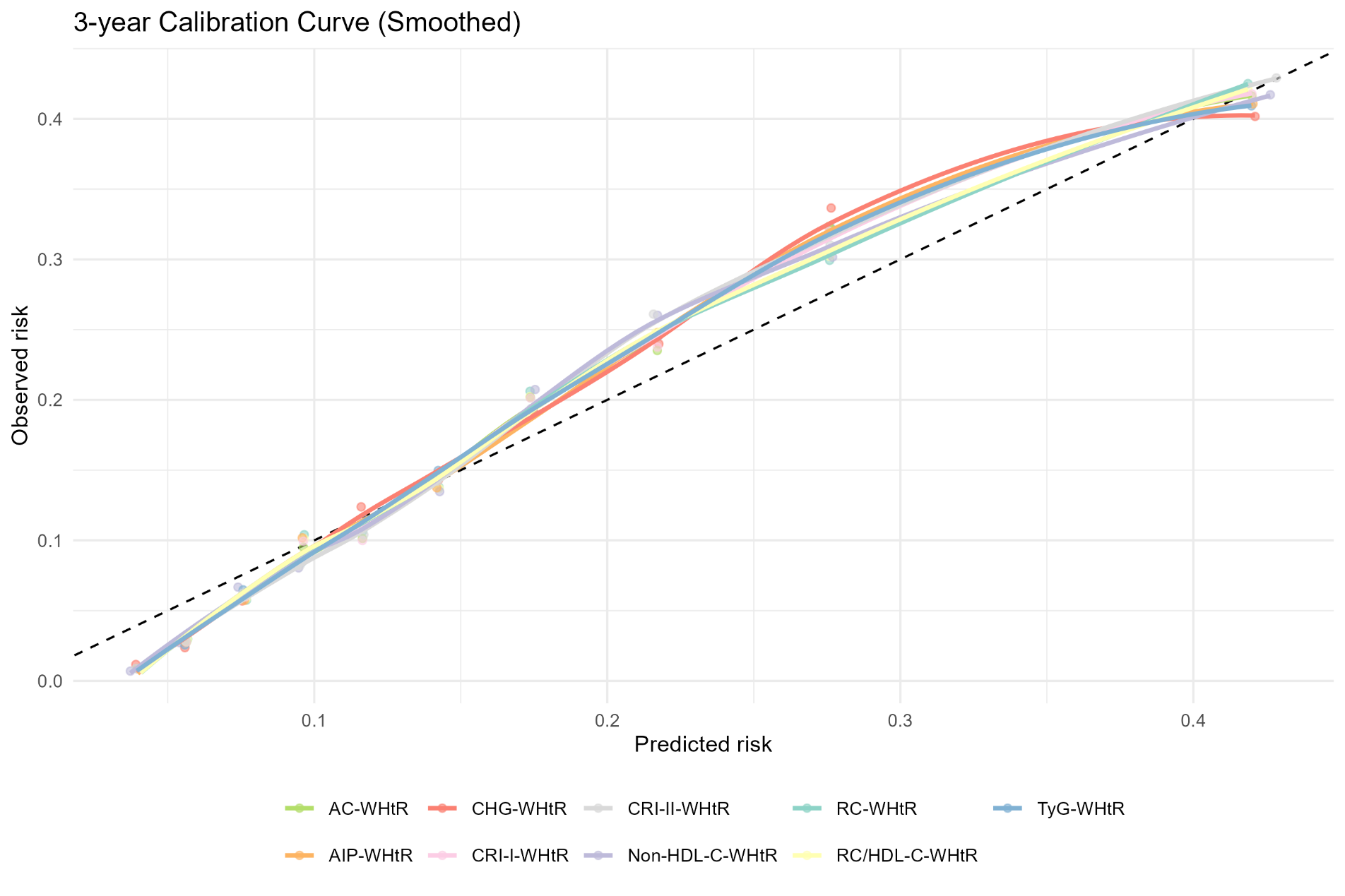


Figure S3. Calibration curves of WHtR-based product indices for 3-year carotid plaque risk prediction.

*Note:* Multiplicative composite indices are constructed by multiplying each cardiometabolic index and WHtR.

Abbreviation: AC, atherogenic coefficient; AIP, atherogenic index of plasma; CHG, cholesterol, high-density lipoprotein, and glucose index; CRI-I, Castelli’s index-I; CRI-II, Castelli’s index-II; Non-HDL-C, non-high density lipoprotein cholesterol; RC, remnant cholesterol; RC/HDL-C, remnant cholesterol and high density lipoprotein cholesterol ratio; TyG, triglyceride-glucose index; WHtR, waist-to-height ratio


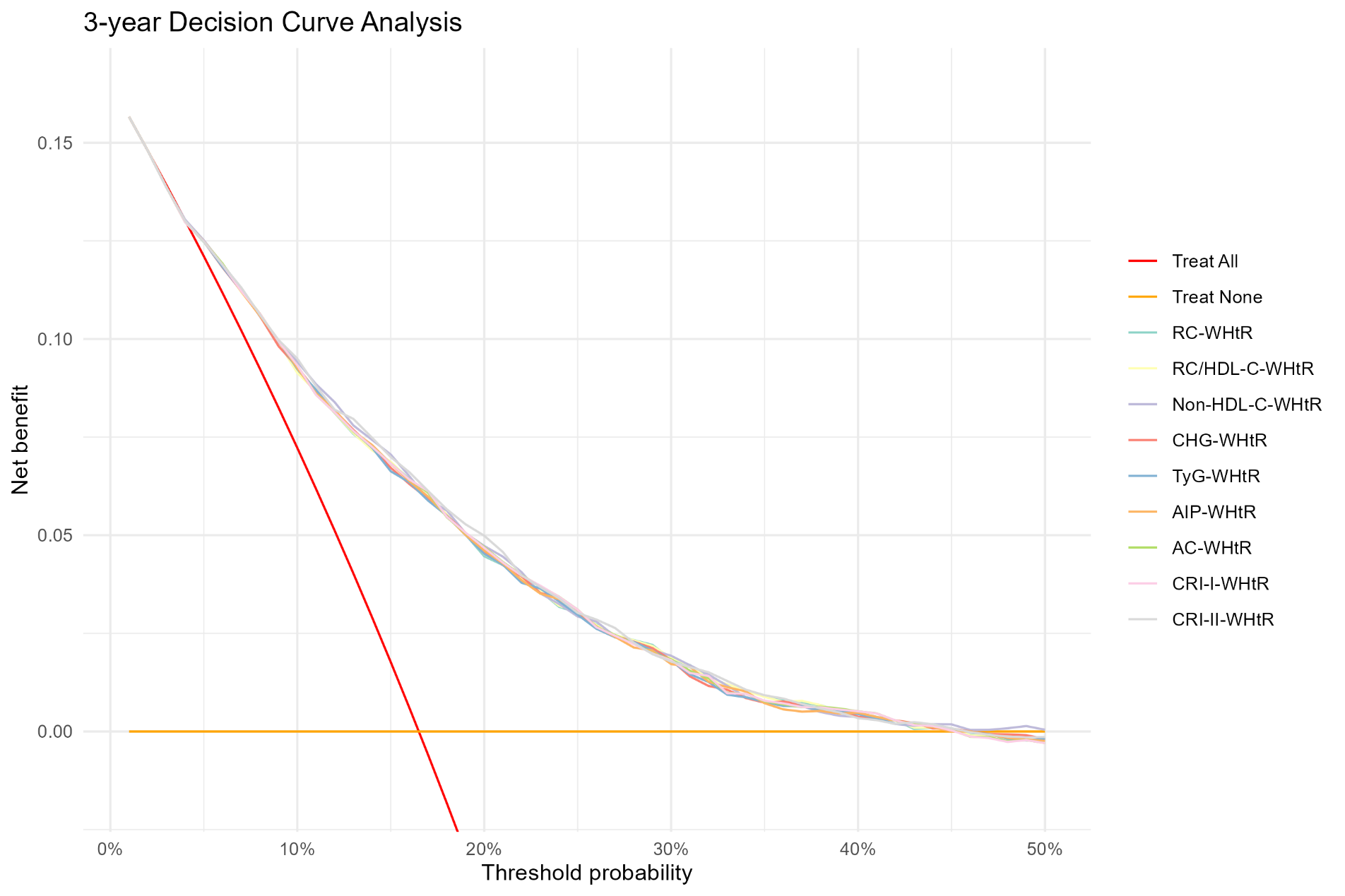


Figure S4. Decision curve analysis of WHtR-based product indices for 3-year carotid plaque risk prediction.

*Note:* Multiplicative composite indices are constructed by multiplying each cardiometabolic index and WHtR.

Abbreviation: AC, atherogenic coefficient; AIP, atherogenic index of plasma; CHG, cholesterol, high-density lipoprotein, and glucose index; CRI-I, Castelli’s index-I; CRI-II, Castelli’s index-II; Non-HDL-C, non-high density lipoprotein cholesterol; RC, remnant cholesterol; RC/HDL-C, remnant cholesterol and high density lipoprotein cholesterol ratio; TyG, triglyceride-glucose index; WHtR, waist-to-height ratio


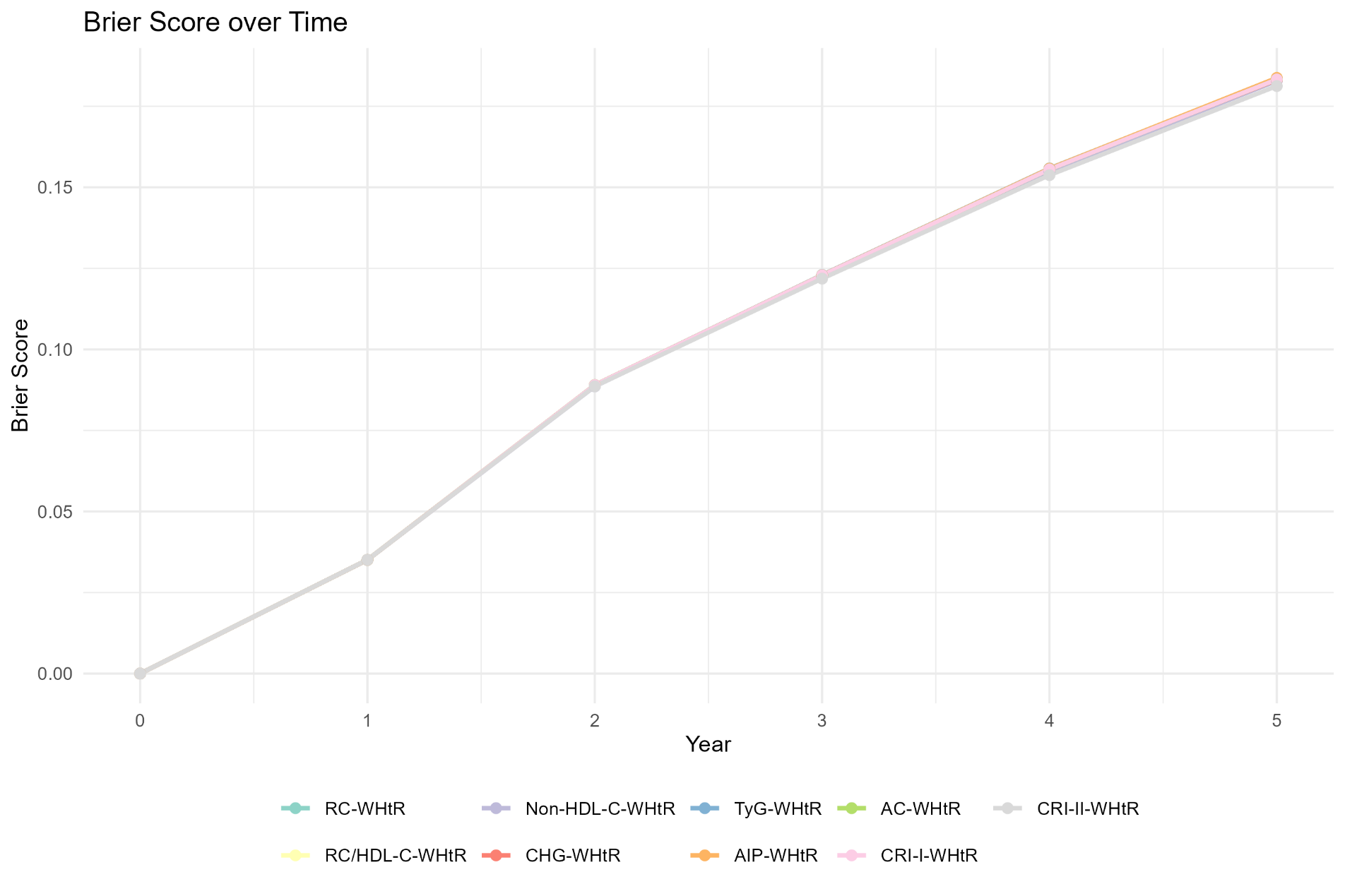


Figure S5. Time-dependent Brier score curves of WHtR-based product indices.

*Note:* Multiplicative composite indices are constructed by multiplying each cardiometabolic index and WHtR.

Abbreviation: AC, atherogenic coefficient; AIP, atherogenic index of plasma; CHG, cholesterol, high-density lipoprotein, and glucose index; CRI-I, Castelli’s index-I; CRI-II, Castelli’s index-II; Non-HDL-C, non-high density lipoprotein cholesterol; RC, remnant cholesterol; RC/HDL-C, remnant cholesterol and high density lipoprotein cholesterol ratio; TyG, triglyceride-glucose index; WHtR, waist-to-height ratio


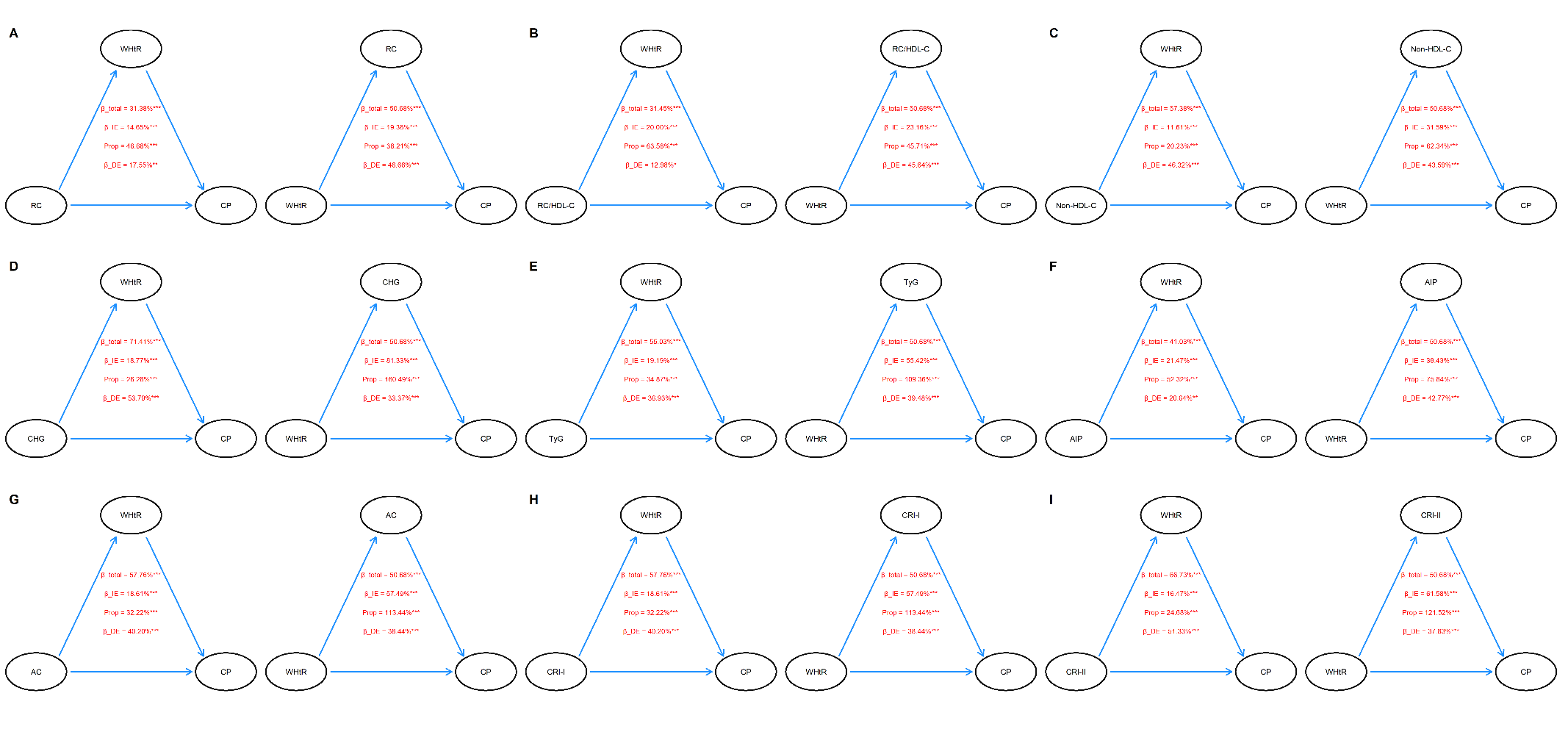


Figure S6. Exploratory analysis of WHtR in relation to cardiometabolic indices and incident carotid plaque.

*Note:* The cardiometabolic indices were categorized into two groups based on the 75% cut-off. *P < 0.05, **P < 0.01, ***P < 0.001.

Abbreviation: AC, atherogenic coefficient; AIP, atherogenic index of plasma; CHG, cholesterol, high-density lipoprotein, and glucose index; CRI-I, Castelli’s index-I; CRI-II, Castelli’s index-II; Non-HDL-C, non-high density lipoprotein cholesterol; RC, remnant cholesterol; RC/HDL-C, remnant cholesterol and high density lipoprotein cholesterol ratio; TyG, triglyceride-glucose index; WHtR, waist-to-height ratio
